# Data-driven cluster analysis identifies three clinical phenotypes in hemodialysis patients

**DOI:** 10.1080/0886022X.2025.2588961

**Published:** 2025-11-20

**Authors:** Canyu Chen, Yifei Lu, Junxiang Qiu, Honglin Xiong, Liang Zhou, Tao Wu

**Affiliations:** ^a^ School of Public Health, Shanghai Jiao Tong University School of Medicine, Shanghai, China; ^b^ Collaborative Innovation Center for Biomedicine, Shanghai University of Medicine & Health Sciences, Shanghai, China; ^c^ Faculty of Medicine, University of Banja Luka, Banja Luka, Bosnia and Herzegovina

**Keywords:** Hemodialysis, machine learning, phenotypes, precision medicine, biomarker patterns

## Abstract

Clinical heterogeneity among hemodialysis patients necessitates precision medicine approaches transcending conventional single-parameter management. Through machine learning analysis of 1,207 maintenance hemodialysis patients, we developed a novel two-tier phenotyping framework integrating unsupervised K-means clustering across 22 clinical indicators with supervised classification using six universally available biomarkers. Five mechanistically informed composite indicators were constructed, including the Middle-Small Molecule Clearance Index (β_2_-microglobulin reduction ratio × *Kt*/*V*) and ferritin–hemoglobin ratio, achieving superior discriminatory capacity over traditional approaches. Three distinct metabolic phenotypes emerged with exceptional stability (Adjusted Rand Index = 0.9181): high retention-inflammatory (19.5%) characterized by dialysis inadequacy and functional iron deficiency, optimal clearance (24.3%) demonstrating superior toxin removal, and intermediate-stable (56.0%) maintaining balanced parameters. The simplified six-parameter model achieved clinically acceptable performance (AUC: 0.893–0.919, accuracy >88%) enabling automated EMR integration. This cross-sectional phenotype discovery represents the foundational step toward precision nephrology, establishing classification frameworks essential for subsequent longitudinal validation studies. The methodology facilitates phenotype-guided interventions: intensified dialysis for high retention-inflammatory patients, clearance optimization for optimal clearance patients, and proactive monitoring for intermediate-stable patients, advancing hemodialysis toward algorithm-driven individualized care with potential to optimize clinical outcomes and resource utilization, pending prospective validation of phenotype-outcome associations.

## Introduction

End-stage renal disease (ESRD) imposes a profound global health burden, affecting over 2 million individuals worldwide, with more than 808,000 in the United States alone, of whom approximately 68% rely on dialysis, primarily hemodialysis [1]. Despite technological advancements, hemodialysis patients face a five-year survival rate of only 42%, with cardiovascular complications contributing to nearly half of all deaths [2]. The economic toll is equally staggering, with Medicare spending on ESRD treatment exceeding $50 billion annually in recent years, representing a substantial portion of the Medicare budget despite ESRD patients comprising just 1% of its population [3]. These alarming statistics underscore the urgent need for innovative strategies to enhance patient outcomes, improve quality of life, and optimize healthcare resource utilization in the face of rising costs and persistent health disparities [4].

Hemodialysis patients exhibit marked clinical heterogeneity, manifesting in diverse complications such as mineral metabolism disorders, anemia, inflammation, and variable dialysis efficiency [5]. Variations in biomarkers like C-reactive protein, hemoglobin, or *Kt*/*V* significantly influence therapeutic responses and clinical outcomes, yet standardized treatment protocols often fail to address this diversity [6]. This mismatch contributes to suboptimal care, highlighting the critical need for precision medicine approaches that tailor interventions to individual patient profiles [7]. Cluster analysis, a data-driven method, has shown promise in identifying patient phenotypes based on multiple clinical indicators, as demonstrated in fields like diabetes, where it has revealed distinct disease trajectories and treatment needs [8].

Prior studies applying cluster analysis in nephrology have provided valuable insights but are limited by their scope and scale. For example, Yu et al. used two-step clustering to explore symptom demands in 167 hemodialysis patients, focusing solely on patient-reported outcomes [9]. Liao et al. analyzed healthcare claims data to identify cost patterns in ESRD patients but did not link these to clinical profiles [10]. Li et al. classified fatigue levels in 167 hemodialysis patients, omitting broader clinical indicators [11], while Komaru et al. employed hierarchical clustering on 46 variables from just 101 patients to predict mortality, limiting generalizability due to the small sample size [12]. These studies, while informative, often relied on restricted indicator sets or small cohorts, failing to capture the multifaceted nature of hemodialysis patient heterogeneity.

Recent large-scale phenotyping studies have demonstrated important advances through registry and EMR approaches. Kanda et al. applied ensemble machine learning to Japan’s national dialysis registry for mortality prediction [13]. Shang et al. developed automated CKD staging algorithms using rule-based and machine learning methods on 1.3 million EMR records [14]. However, these population-scale studies employ static classification using conventional parameters, leaving critical gaps in dynamic patient monitoring and clinical translation.

Our study addresses these methodological gaps through three key innovations: (1) a dynamic state classification designed for repeated clinical application during routine care, enabling therapeutic monitoring through phenotype transition assessment; (2) a two-tier phenotyping framework that uniquely integrates comprehensive research analytics with practical clinical deployment; (3) mechanistically informed composite indicators that mathematically integrate pathophysiological domains. These advances transform population insights into actionable clinical decisions.

This study addresses these gaps by applying K-means clustering to a comprehensive panel of 22 clinical indicators – spanning inflammation, nutrition, anemia, mineral metabolism, dialysis efficiency, and electrolyte changes – in a large cohort of 1,207 hemodialysis patients. By identifying distinct phenotypes and elucidating their clinical significance, we aim to provide a robust framework for personalized treatment strategies that can optimize dialysis care. We hypothesize that unique phenotypes exist, each defined by specific clinical profiles that can guide tailored interventions, thereby advancing precision medicine in nephrology and improving patient outcomes. While this cross-sectional study cannot establish outcome associations, it provides the essential classification framework for future prospective validation studies.

## Methods

### Research design and study participants

This cross-sectional observational study was conducted from 1 January 2020 to 31 December 2021, at a tertiary hospital in Shanghai, China. The study protocol strictly adhered to the Strengthening the Reporting of Observational Studies in Epidemiology (STROBE) guidelines [15]. Verbal informed consent for research use of de-identified clinical data was obtained from all patients during routine care. Additional consent was waived for this retrospective analysis per institutional review board approval (CMEC-2023-KT-05). The study sample size (*n* = 1,207) was sufficient to detect a medium effect (*d* = 0.5) with 95% statistical power at an alpha level of 0.05.

Our analytical cohort comprised 1,271 maintenance hemodialysis patients systematically extracted from institutional electronic health records following application of standard clinical exclusion criteria including pediatric patients, individuals with inadequate dialysis vintage, active malignancies, acute clinical instability, and excessive missing data. Subsequent machine learning preprocessing employed Isolation Forest algorithm for outlier detection, identifying 64 patients (5.04% contamination rate) with anomalous biomarker profiles that could potentially compromise clustering validity. This systematic exclusion yielded our final analytical cohort of 1,207 patients, ensuring data integrity essential for robust unsupervised machine learning analysis.

### Data collection

Clinical data were extracted from electronic medical records using standardized protocols across five domains: inflammation/nutrition (white blood cell count, C-reactive protein (CRP), albumin), anemia (hemoglobin, ferritin, TSAT), mineral metabolism (calcium, phosphorus, iPTH, alkaline phosphatase), dialysis efficiency (*Kt*/*V*, pre-dialysis β_2_-microglobulin, β_2_-microglobulin reduction ratio, pre-dialysis BUN), and electrolyte fluctuation (pre-/post-dialysis HCO_3_^−^, K^+^, Na^+^ changes).

Dialysis prescription parameters including blood flow rate, dialysate flow rate, treatment time, and ultrafiltration volume were systematically extracted for 987 patients (81.8%) to enable treatment confounding assessment, though medication data (ESA, iron supplementation, vitamin D) were not available in the retrospective dataset.

Blood samples were collected according to a standardized protocol. Pre-dialysis samples were obtained immediately before hemodialysis initiation, while post-dialysis samples were collected using a standardized slow-flow method to minimize recirculation effects. Laboratory measurements included standard CRP assays, which are clinically appropriate for hemodialysis patients with elevated inflammatory markers.

Blood samples were obtained according to standardized single-timepoint protocols to ensure methodological consistency. While this approach may introduce classification uncertainty due to inherent weekly fluctuations in inflammatory markers and iron metabolism indices, composite indicator construction and robust standardization protocols minimize temporal bias effects. The phenotyping framework enables repeated clinical application for dynamic phenotype monitoring.

### Statistical analysis

#### Data preprocessing

Missing values (comprising <4% of the total dataset, 51 missing data points) were handled using median imputation. This approach was chosen over multiple imputation based on three considerations: the missing rate was below the 5% threshold, the missing pattern exhibited completely random distribution (missing completely at random, MCAR) characteristics, and median imputation offers greater robustness to outliers compared to mean imputation.

We applied Yeo-Johnson power transformation to skewed variables to improve normality [16]. For each variable, transformation parameter *λ* was determined through maximum-likelihood estimation to achieve optimal normal distribution approximation. Subsequently, robust scaling was performed by subtracting the median and dividing by the interquartile range (IQR), a method selected to reduce outlier influence on standardization.

To capture the multidimensional pathophysiology of hemodialysis patients, we constructed five composite indicators based on established clinical evidence and mathematical principles:

*Calcium–phosphorus product*: Calculated as serum calcium (mg/dL) × serum phosphorus (mg/dL), yielding mg^2^/dL^2^. This product quantifies the thermodynamic driving force for vascular calcification based on the solubility product constant (Ksp) of calcium–phosphate crystals. When exceeding 55 mg^2^/dL^2^, spontaneous precipitation occurs through vascular smooth muscle cell transformation to osteoblast-like phenotypes via Cbfa1/Runx2 signaling [17]. Meta-analysis of 327,644 patients demonstrates 18% increased mortality per 1 mg/dL phosphorus elevation [18].

*Inflammation–nutrition ratio*: Computed as CRP (mg/L)/albumin (g/L), this ratio integrates opposing acute-phase responses to discriminate malnutrition–inflammation complex syndrome (MICS). The mathematical construction leverages cytokine-mediated (IL-1β, IL-6, TNF-α) suppression of albumin synthesis concurrent with CRP stimulation through NF-κB pathways. Recent validation in 6,679 hemodialysis patients established superior prognostic performance (HR 1.54–2.89) compared to individual markers [19,20].

*Ferritin–hemoglobin ratio*: Calculated as ferritin (ng/mL)/hemoglobin (g/dL), quantifying functional iron deficiency where inflammation-induced hepcidin elevation causes iron sequestration despite adequate stores. This ratio captures the clinical paradox of elevated ferritin with poor erythropoietin response, affecting up to 85% of hemodialysis patients through hepcidin-mediated ferroportin downregulation [21].

*Middle-Small Molecule Clearance Index*: Constructed as β_2_-microglobulin reduction ratio (%) × *Kt*/*V*, providing comprehensive dialysis adequacy assessment. The multiplication integrates small molecule clearance (*Kt*/*V*) with middle molecule removal (β_2_-microglobulin, 11,800 Da), addressing the limitation that urea kinetics correlate poorly with cardiovascular uremic toxins. Meta-analysis demonstrates 3% mortality increase per 1 mg/L β_2_-microglobulin elevation, independent of *Kt*/*V* [22,23].

*Electrolyte Disturbance Index*: Sum of absolute electrolyte changes (|ΔNa| + |ΔK| + |ΔHCO_3_|), quantifying total intradialytic flux. This construction recognizes that rapid shifts trigger arrhythmogenic mechanisms through baroreceptor activation and membrane potential alterations, with validated correlation to ventricular ectopy frequency (*r* = 0.68) [24].

The multiplicative construction of composite indicators follows established physiological principles and clinical precedents in nephrology. Multiplicative formulations are theoretically appropriate when biomarkers interact through cooperative pathways, as demonstrated by the FDA-approved [TIMP-2] × [IGFBP7] composite biomarker for acute kidney injury assessment [25]. Theoretical discriminative accuracy analyses consistently demonstrate superior performance of multiplicative models compared to additive approaches in mathematical modeling scenarios [26]. The multiplicative approach reflects physiological integration consistent with kidney function assessment requiring complex mathematical relationships [27].

Near-zero variance features were removed, and multicollinearity was controlled through variance inflation factor assessment.

#### Cluster analysis and outlier detection

Outlier detection utilized the Isolation Forest algorithm with a contamination parameter of 0.05, determined through preliminary assessment of data distribution and aligned with clinical data quality control requirements. The algorithm used default parameters, including 100 trees, identifying and excluding 64 anomalous data points (5.04% of the initial cohort).

Cluster analysis utilized the K-means algorithm, selected for three core advantages: computational efficiency suitable for large medical datasets, cluster centers directly corresponding to clinical indicators enhancing interpretability, and extensive validation in medical patient stratification. To mitigate K-means sensitivity to initial centroid positions, we implemented the K-means++ initialization method [28], which improves convergence likelihood by selecting distant initial centroids.

K-means clustering identifies patient subgroups by partitioning observations into clusters that minimize within-group biomarker variance while maximizing between-group separation [28]. The algorithm assumes patients with similar pathophysiological states exhibit comparable biomarker profiles, forming distinct clusters in multidimensional parameter space.

The algorithm employs iterative optimization beginning with initial cluster center placement using K-means++ methodology to ensure stable convergence. Subsequently, each patient is assigned to the nearest cluster center based on Euclidean distance calculations across standardized biomarkers, followed by recalculation of cluster centers as arithmetic means of assigned patients. This assignment-update cycle continues until cluster membership stabilizes, indicating optimal patient groupings that minimize within-cluster biomarker variability [28].

Resulting cluster centroids represent mean biomarker profiles characteristic of each metabolic state, providing clinically interpretable phenotypes that reflect actual population distributions rather than arbitrary cutoffs. This data-driven approach enables identification of complex biomarker interactions potentially obscured by single-parameter classifications.

**Figure 1.**
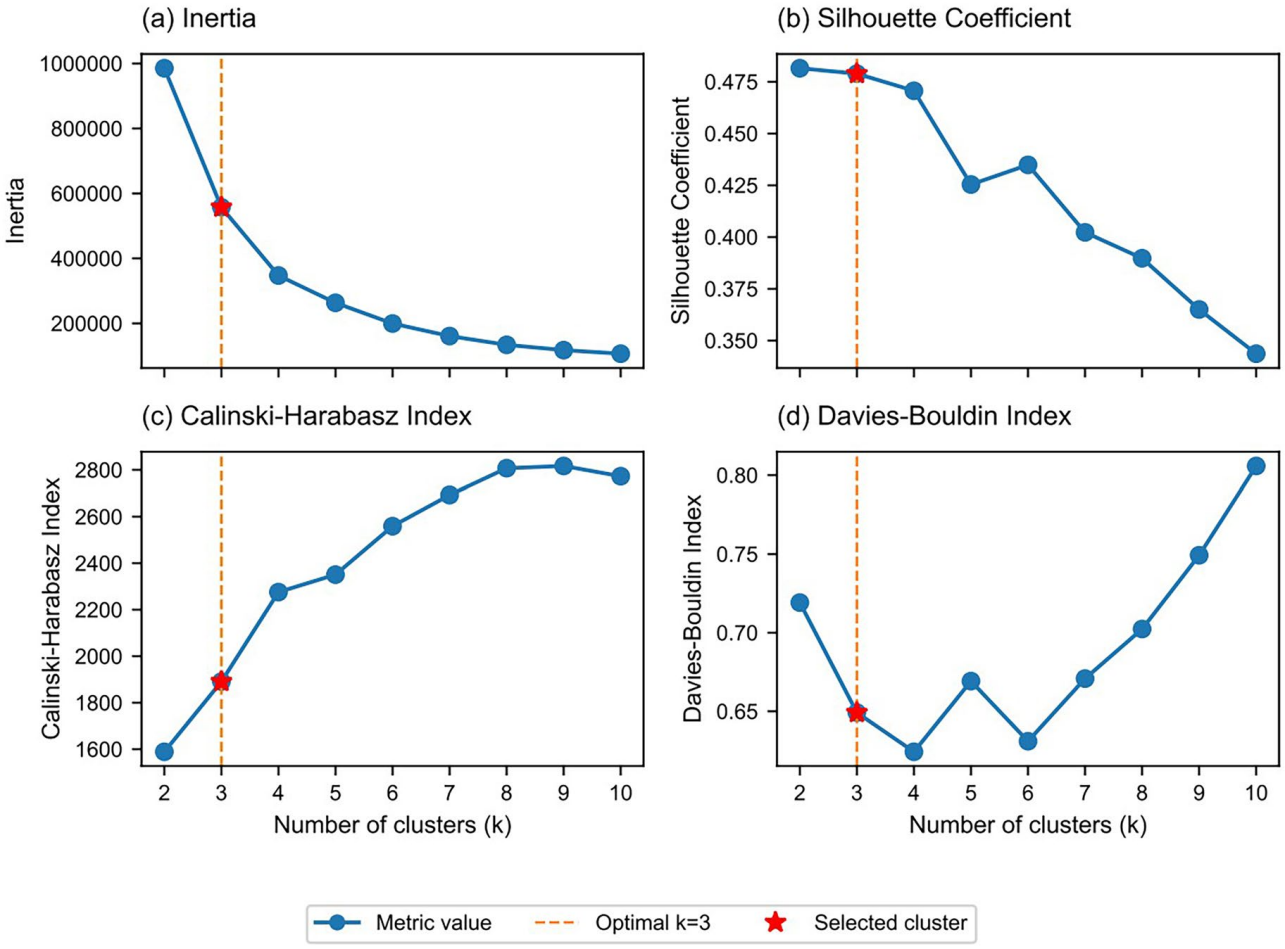
Evaluation metrics for optimal cluster number selection. (a) Inertia represents the sum of squared distances within clusters; (b) silhouette coefficient measures the intra-cluster similarity and inter-cluster dissimilarity; (c) Calinski–Harabasz index evaluates the ratio of between-cluster to within-cluster dispersion; (d) Davies–Bouldin index assesses the similarity between clusters. The vertical dashed line at *k* = 3 indicates the optimal number of clusters.

To determine the optimal number of clusters, we analyzed configurations from *k* = 2 to *k* = 10 using multiple evaluation metrics (Figure 1): inertia values (intra-cluster compactness), silhouette coefficient (sample assignment clarity), Calinski–Harabasz index (between-cluster to within-cluster variance ratio), and Davies–Bouldin index (cluster separation). Empirical analysis supported *k* = 3 as optimal: inertia decreased significantly from *k* = 2 to *k* = 3 (reduction of 428,114.6) with subsequent diminishing reductions, while silhouette coefficient at *k* = 3 (0.4789) maintained a relatively high level.

The selection of *k* = 3 was based on three considerations: parsimony to avoid overfitting, sufficient phenotype sample sizes (smallest phenotype: 236 patients, 19.5%), and alignment with clinical risk stratification practices (low, medium, high risk) [29], enhancing clinical applicability.

To verify algorithm selection appropriateness, we compared three clustering algorithms under *k* = 3 conditions (Table 1). Although hierarchical clustering showed advantages in certain metrics, K-means exhibited superior Calinski–Harabasz index performance (1,890.91 vs. 1,604.80), indicating better cluster separation. The final model achieved silhouette coefficient 0.4789, Calinski–Harabasz index 1,890.91, and Davies–Bouldin index 0.6492.

**Table 1.** Performance comparison of clustering algorithms at *k* = 3.

	Silhouette coefficient	Calinski–Harabasz index	Davies–Bouldin index
K-means	0.4789	1,890.91	0.6492
Agglomerative	0.4805	1,604.80	0.5794
Gaussian mixture	−0.0224	104.46	3.5778

For result validation, we implemented a rigorous internal validation strategy using bootstrap resampling with 500 iterations, recalculating the clustering algorithm and computing indices in each iteration. Results indicated high stability (Average Rand Index: 0.8871; Average Adjusted Rand Index: 0.7672). Additionally, 10-fold cross-validation yielded an Adjusted Rand Index of 0.9181, further confirming classification robustness.

Clustering results were visualized through *t*-distributed stochastic neighbor embedding, selected for its superior performance in preserving high-dimensional data topology [30], displaying distribution patterns and boundary characteristics of the three phenotypes.

#### Simplified clinical implementation model

To address EMR data variability constraints, we developed a six-parameter classification framework utilizing routine hemodialysis monitoring data: *Kt*/*V*, β_2_-microglobulin reduction rate, hemoglobin, ferritin, ferritin–hemoglobin ratio, and Middle Molecule Clearance Index. The supervised learning algorithms were trained using phenotype labels derived from the comprehensive parameter K-means clustering analysis as ground truth classification.

Three supervised learning algorithms were implemented: Random Forest XGBoost and SVM. Dataset partitioning employed 70:30 stratified sampling (training: *n* = 844, testing: *n* = 363). Cross-validation utilized 10-fold methodology with Rand Index stability assessment.

Performance evaluation included accuracy, precision, recall, *F*1-score, and area under the receiver operating characteristic curve (AUC). Additionally, to address clinical implementation requirements, a transparent scoring framework Hemodialysis Phenotype Clinical Score (HPCS) was developed using weighted parameter contributions to provide calculable classification thresholds as an alternative to machine learning predictions. Concordance between simplified model predictions and original clustering classifications was assessed through Rand Index calculations.

#### Phenotypes characteristic analysis

To characterize identified phenotypes, we employed one-way analysis of variance (ANOVA) with Bonferroni’s correction for normally distributed variables, while Kruskal–Wallis tests were applied for non-parametric data. Effect sizes were calculated using eta squared (*η*^2^), with values <0.06, 0.06–0.14, and >0.14 representing small, medium, and large effects, respectively [31]. Additionally, to enhance feature importance interpretability, SHAP (SHapley Additive exPlanations) analysis was performed on a random forest classifier trained to distinguish the identified phenotypes, providing quantitative assessment of each clinical indicator’s contribution to phenotype prediction. Dominant characteristics of each phenotypes were identified by calculating standardized performance (*Z*-scores) of indicator groups. Statistical significance was set at *p* < 0.05.

Clinical significance assessment utilized Kidney Disease Outcomes Quality Initiative (KDOQI) guidelines as interpretive benchmarks. Developed by the National Kidney Foundation, KDOQI recommendations establish evidence-based therapeutic targets for dialysis adequacy (*Kt*/*V* ≥ 1.4), anemia management (hemoglobin 10.0–12.0 g/dL, transferrin saturation ≥20%), and mineral metabolism control (serum calcium 8.4–10.2 mg/dL, phosphorus 3.5–5.5 mg/dL, intact parathyroid hormone 150–300 pg/mL) [32]. These thresholds provided standardized frameworks for evaluating the clinical relevance of machine learning-derived phenotypic classifications.

Analyses utilized Python 3.12 with scikit-learn ecosystem, maintaining documented workflows for complete reproducibility.

To assess temporal phenotype stability, we analyzed 349 patients (28.9%) with 3-month follow-up data using Cohen’s kappa statistic for concordance evaluation.

Treatment confounding was evaluated using prescription data from 987 patients (81.8%). One-way ANOVA compared dialysis parameters (blood flow rate, dialysate flow rate, treatment time, ultrafiltration volume) across phenotypes. Multiple linear regression quantified prescription-explained variance in key discriminatory biomarkers. Residual analysis after treatment adjustment assessed phenotype independence from prescription effects.

This investigation implements a dynamic state classification methodology designed for repeated clinical application during routine hemodialysis care. The machine learning framework identifies current metabolic states based on contemporaneous biomarker combinations rather than static patient characteristics, enabling real-time clinical decision support. State transitions represent clinically valuable information regarding treatment response and disease progression, paralleling established dynamic assessment systems in critical care medicine.

## Results

### Baseline characteristics of the study population

The final cohort comprised 1,207 maintenance hemodialysis patients (56.8% male; mean age, 58.7 ± 14.3 years; median dialysis vintage, 4.2 years [IQR: 2.1–7.8]). Key baseline parameters demonstrated substantial clinical heterogeneity: median CRP 2.31 mg/L (IQR: 0.97–5.67), mean hemoglobin 109.11 ± 15.20 g/L, mean *Kt*/*V* 1.56 ± 0.38, and median ferritin 167.00 ng/mL (IQR: 38.15–409.45), reflecting the diverse metabolic states characteristic of maintenance hemodialysis populations. Baseline clinical characteristics are summarized in Table 2.

**Table 2.** Baseline clinical characteristics of hemodialysis patients (*n* = 1,207).

Characteristic	Value
Demographic features	
Age (years)	58.7 ± 14.3
Sex (male/female)	686/521 (56.8%/43.2%)
Dialysis vintage (years)	4.2 (2.1–7.8)
Inflammation/nutrition indicators	
White blood cell count (×10^9^/L)	5.90 (4.86–7.04)
C-reactive protein (mg/L)	2.31 (0.97–5.67)
Serum albumin (g/L)	38.64 ± 3.55
Inflammation–nutrition ratio	0.06 (0.02–0.15)
Anemia-related indicators	
Hemoglobin (g/L)	109.11 ± 15.20
Ferritin (ng/mL)	167.00 (38.15–409.45)
Transferrin saturation (%)	24.87 (18.89–32.91)
Ferritin–hemoglobin ratio	1.59 (0.34–3.93)
Mineral metabolism indicators	
Serum calcium (mmol/L)	2.29 ± 0.25
Serum phosphorus (mmol/L)	1.81 ± 0.55
Intact parathyroid hormone (pg/mL)	294.80 (146.55–490.50)
Alkaline phosphatase (U/L)	85.00 (68.00–108.00)
Calcium-phosphorus product (mmol^2^/L^2^)	4.16 ± 1.36
Dialysis efficiency indicators	
*Kt*/*V*	1.56 ± 0.38
Pre-dialysis β_2_-microglobulin (mg/L)	34.60 (29.70–42.18)
β_2_-microglobulin reduction rate (%)	77.51 (70.32–81.93)
Pre-dialysis blood urea nitrogen (mmol/L)	27.19 ± 7.01
Middle-Small Molecule Clearance Index	116.75 ± 40.97
Electrolyte fluctuation indicators	
Bicarbonate change (mmol/L)	4.90 (3.49–6.50)
Potassium change (mmol/L)	1.40 (0.92–1.80)
Sodium change (mmol/L)	3.00 (1.00–5.00)
Electrolyte Disturbance Index	9.00 (7.00–12.20)

Data are presented as mean ± SD or median (IQR).

### Clinical metabolic state identification through dynamic classification analysis

**Figure 2.**
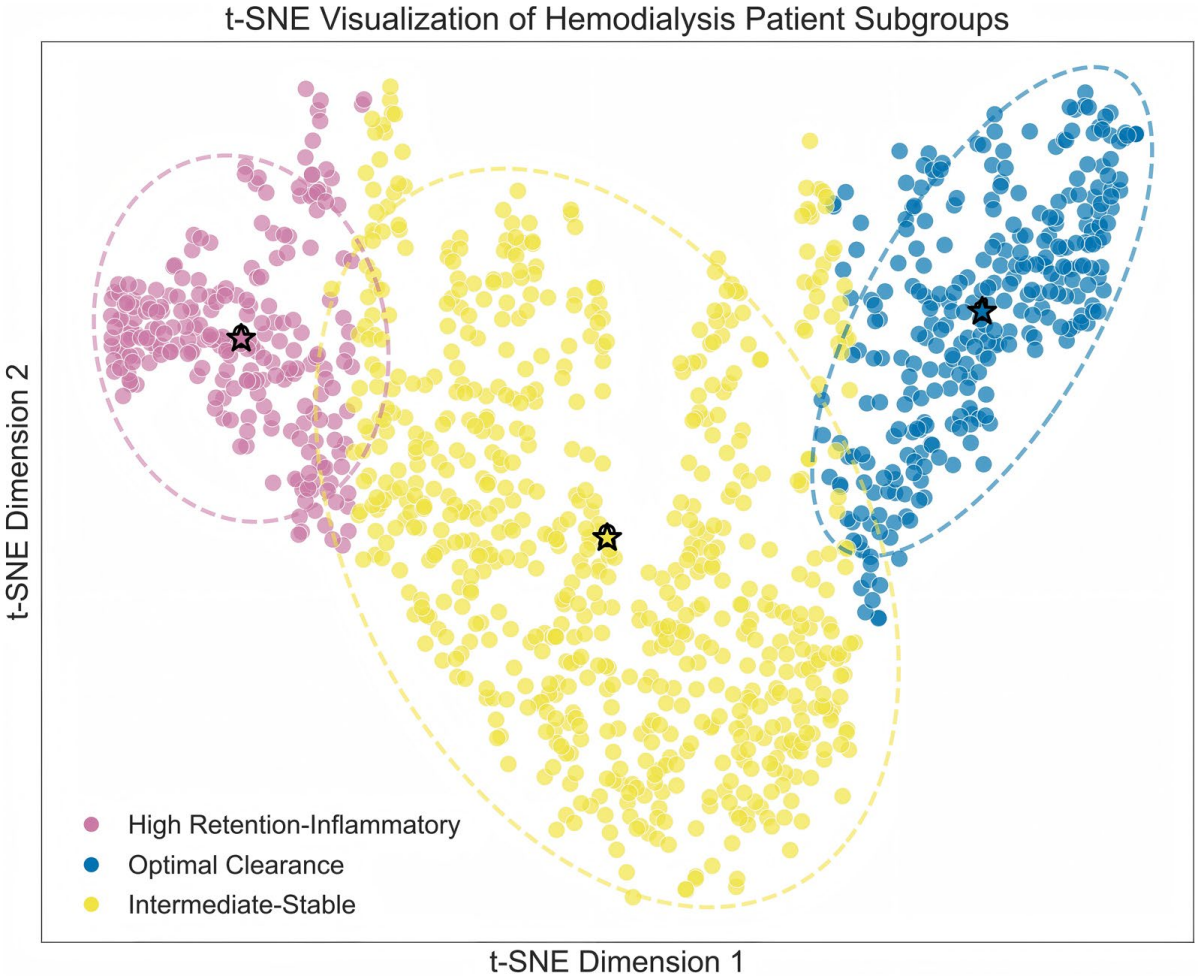
t-SNE visualization of hemodialysis patient phenotypes. High retention-inflammatory (pink, *n* = 236, 19.5%), optimal clearance (blue, *n* = 294, 24.3%), and intermediate-stable (yellow, *n* = 677, 56.0%). Spatial separation demonstrates distinct clinical profiles based on standardized indicators. Dashed circles indicate cluster boundaries; stars denote cluster centroids.

K-means clustering of 22 clinical indicators identified three distinct hemodialysis patient phenotypes (Figure 2): high retention-inflammatory (*n* = 236, 19.5%), optimal clearance (*n* = 294, 24.3%), and intermediate-stable (*n* = 677, 56.0%).

These phenotypes reflect distinct pathophysiological profiles. The high retention-inflammatory phenotype demonstrates pronounced uremic toxin accumulation (*Kt*/*V* 1.18 ± 0.31) with elevated inflammation. The optimal clearance phenotype exhibits superior dialysis efficiency (*Kt*/*V* 2.00 ± 0.25) and reduced inflammatory markers. The intermediate-stable phenotype, representing the majority of patients, maintains balanced clinical parameters.

t-SNE dimensionality reduction visualization (Figure 2) reveals the distinct distribution patterns of these three phenotypes within the high-dimensional feature space. The high retention-inflammatory and optimal clearance phenotypes demonstrate maximal spatial separation, reflecting fundamental differences in their clinical profiles. The intermediate-stable phenotype, occupying the central region, exhibits transitional boundaries with both other phenotypes, consistent with its role as an intermediate clinical state.

This classification framework captures the inherent heterogeneity in hemodialysis patients, providing a foundation for precision medicine approaches in dialysis care. The identified clinical states represent distinct metabolic configurations based on contemporaneous biomarker patterns, with state membership reflecting current physiological status that may evolve in response to therapeutic interventions or disease progression.

### Simplified model validation

The six-parameter framework achieved clinically acceptable performance across algorithms: Random Forest (AUC = 0.919, Accuracy = 88.4%), XGBoost (AUC = 0.908, Accuracy = 88.2%), and SVM (AUC = 0.893, Accuracy = 89.0%). Cross-validation demonstrated high stability (Rand Index 0.902–0.919), confirming robust concordance with the original 22-parameter clustering classifications. Comprehensive performance validation data, including algorithm comparison matrices are detailed in Supplementary Materials S24–S30.

SHAP analysis confirmed *Kt*/*V* and β_2_-microglobulin reduction rate as primary discriminators across phenotypes, while the ferritin–hemoglobin ratio provided critical discriminative information for functional iron deficiency identification. The validated framework demonstrates translational potential for precision nephrology applications through automated phenotype classification using routine clinical monitoring parameters. To further enhance clinical applicability, a preliminary clinical scoring framework (HPCS) was developed, achieving internal validation accuracy of 85.2% (*κ* = 0.81) while requiring external validation for broader implementation (Supplementary Tables S1 and S2).

### Temporal stability assessment

Preliminary longitudinal analysis of 349 patients with 3-month follow-up revealed substantial phenotype stability (Cohen’s *κ* = 0.686, *p* < 0.001), with 79.1% maintaining their baseline classification (high retention-inflammatory: 78.0%, optimal clearance: 78.6%, intermediate-stable: 80.7%). Population-level distribution remained unchanged (*χ*^2^ = 2.99, *p* = 0.224), suggesting phenotypic equilibrium (Supplementary Figure S32D).

### Treatment confounding assessment

Prescription data analysis (*n* = 987) revealed no significant differences in dialysis parameters across phenotypes (all *p* > 0.05; Supplementary Table S3). Treatment variables explained minimal biomarker variance: *Kt*/*V* (*R*^2^ = 0.137), β_2_-microglobulin reduction (*R*^2^ = 0.112), and ferritin/hemoglobin ratio (*R*^2^ = 0.089). After adjustment for prescription parameters, phenotype-specific biomarker differences remained significant (all adjusted *p* < 0.001; Supplementary Figure S32), confirming that >80% of observed heterogeneity reflects biological rather than treatment-induced variation.

### Phenotype characterization

To characterize identified phenotypes, we employed one-way ANOVA with Bonferroni’s correction for normally distributed variables (Table 3).

**Table 3.** Comparison of clinical indicators across three patient phenotypes.

Indicators	High retention-inflammatory phenotype	Optimal clearance phenotype	Intermediate-stable phenotype	*p* Value	*η* ^2^	Pairwise comparisons* (all *p* < 0.001)
Dialysis efficiency indicators						
*Kt*/*V*	1.18 ± 0.31	2.00 ± 0.25	1.52 ± 0.17	<0.001	0.608	0–1, 0–2, 1–2
β_2_-microglobulin reduction rate (%)	54.74 ± 21.86	83.09 ± 4.32	76.38 ± 5.61	<0.001	0.454	0–1, 0–2, 1–2
Middle-Small Molecule Clearance Index	61.99 ± 24.99	166.53 ± 21.81	115.71 ± 14.55	<0.001	0.77	0–1, 0–2, 1–2
Pre-dialysis BUN (mmol/L)	26.81 ± 6.74	28.53 ± 6.31	26.83 ± 6.76	<0.01	0.012	1–2
Pre-dialysis β_2_-microglobulin (mg/L)	37.16 ± 14.96	35.81 ± 8.56	36.91 ± 10.78	0.29	0.002	–
Inflammation/nutrition indicators						
CRP (mg/L)	4.87 ± 5.68	3.36 ± 4.47	4.12 ± 5.15	<0.01	0.009	–
Albumin (g/L)	38.49 ± 3.63	38.41 ± 3.21	38.96 ± 3.38	0.029	0.006	–
Inflammation–nutrition ratio	0.13 ± 0.15	0.09 ± 0.12	0.11 ± 0.14	<0.01	0.009	–
White blood cell count (×10^9^/L)	6.21 ± 1.78	5.84 ± 1.71	6.17 ± 1.98	0.024	0.006	–
Anemia-related indicators						
Hemoglobin (g/L)	106.06 ± 15.70	110.93 ± 11.50	110.79 ± 13.88	<0.001	0.019	0–1, 0–2
Ferritin (ng/mL)	333.16 ± 315.14	203.23 ± 274.42	262.26 ± 296.23	<0.001	0.021	0–1
TSAT (%)	27.87 ± 12.81	26.57 ± 10.09	27.26 ± 12.34	0.072	0.004	–
Ferritin–hemoglobin ratio	3.22 ± 2.98	1.86 ± 2.44	2.46 ± 2.79	<0.001	0.026	0–1, 0–2
Mineral metabolism indicators						
Calcium (mmol/L)	2.23 ± 0.26	2.31 ± 0.23	2.32 ± 0.24	<0.001	0.019	0–1, 0–2
Phosphorus (mmol/L)	1.83 ± 0.57	1.80 ± 0.50	1.83 ± 0.52	0.737	0.001	–
iPTH (pg/mL)	342.26 ± 269.78	392.30 ± 310.56	360.31 ± 298.78	0.132	0.003	–
Alkaline phosphatase (U/L)	92.81 ± 36.09	93.65 ± 34.16	93.09 ± 57.71	0.978	0.000	–
Calcium–phosphorus product (mmol^2^/L^2^)	4.10 ± 1.39	4.17 ± 1.22	4.25 ± 1.31	0.313	0.002	–
Electrolyte fluctuation indicators						
HCO_3_ change (mmol/L)	5.14 ± 2.25	5.12 ± 2.35	4.87 ± 2.23	0.149	0.003	–
K change (mmol/L)	1.28 ± 0.75	1.26 ± 1.05	1.32 ± 0.86	0.617	0.001	–
Na change (mmol/L)	3.31 ± 2.68	3.45 ± 2.64	3.31 ± 2.79	0.749	0.000	–
Electrolyte Disturbance Index	9.80 ± 4.33	10.08 ± 4.33	9.64 ± 4.23	0.337	0.002	–

Data are presented as mean ± SD unless otherwise indicated. All continuous variables tested using one-way ANOVA with Bonferroni’s post hoc correction (*α* = 0.05). *η*^2^: eta-squared effect size, with values <0.06, 0.06–0.14, and >0.14 representing small, medium, and large effects, respectively.

* Pairwise comparisons: 0 = high retention-inflammatory, 1 = optimal clearance, 2 = intermediate-stable phenotype. Significant differences (*p* < 0.05) indicated by numeric pairing; (−) denotes no significant difference.

#### High retention-inflammatory phenotype

This phenotype was characterized by severe dialysis inadequacy, with a mean *Kt*/*V* of 1.18 ± 0.31, substantially below the KDOQI guideline recommendations [32]. Patients exhibited markedly impaired β_2_-microglobulin reduction rates (54.74 ± 21.86%) and the lowest Middle-Small Molecule Clearance Index (61.99 ± 24.99). The persistent inflammatory activation was evidenced by elevated CRP (median 4.87 mg/L) and inflammation–nutrition ratio (0.13 ± 0.15).

The most distinctive feature of this phenotype was severe iron metabolism dysfunction, with ferritin–hemoglobin ratio reaching 3.22 ± 2.98, substantially higher than other phenotypes. Despite elevated ferritin levels (333.16 ± 315.14 ng/mL), hemoglobin remained low at 106.06 ± 15.70 g/L, with TSAT of 27.87 ± 12.81%, representing classic functional iron deficiency. Additionally, this phenotype demonstrated the lowest serum calcium (2.23 ± 0.26 mmol/L), indicating multifaceted metabolic derangements. This phenotype can be objectively identified by inadequate dialysis clearance (*Kt*/*V* < 1.4) combined with severe functional iron deficiency (ferritin–hemoglobulin ratio >3.0), providing reproducible criteria for high-risk patient identification.

#### Optimal clearance phenotype

This phenotype represented optimal dialysis performance, with *Kt*/*V* achieving 2.00 ± 0.25 and the highest Middle-Small Molecule Clearance Index (166.53 ± 21.81). β_2_-microglobulin reduction rates (83.09 ± 4.32%) approximated normal renal function levels. Inflammatory markers remained low, with CRP at 3.36 mg/L and inflammation–nutrition ratio at 0.09 ± 0.12, approaching those of healthy individuals.

The phenotype exhibited optimal iron metabolism, with ferritin–hemoglobin ratio of only 1.86 ± 2.44, maintaining the highest TSAT (26.57 ± 10.09%) and hemoglobin levels (110.93 ± 11.50 g/L). Mineral metabolism parameters (calcium 2.31 ± 0.23 mmol/L, phosphorus 1.80 ± 0.50 mmol/L) approximated normal ranges, with well-controlled calcium-phosphorus product (4.17 ± 1.22 mmol^2^/L^2^). The phenotype can be objectively defined by superior dialysis performance (*Kt*/*V* > 1.8) with efficient iron utilization (ferritin–hemoglobulin ratio <2.0), establishing clear benchmarks for optimal management.

#### Intermediate-stable phenotype

This phenotype represented the majority of patients, with dialysis adequacy indicators (*Kt*/*V* 1.52 ± 0.17) meeting basic clinical requirements. β_2_-microglobulin reduction rates (76.38 ± 5.61%) and Middle-Small Molecule Clearance Index (115.71 ± 14.55) were moderately effective. CRP levels (4.12 ± 5.15 mg/L) and inflammation–nutrition ratio (0.11 ± 0.14) indicated mild inflammatory states. ferritin–hemoglobin ratio (2.46 ± 2.79) fell between the other phenotypes.

This phenotype demonstrated optimal electrolyte stability, exhibiting the lowest Electrolyte Disturbance Index (9.64 ± 4.23), suggesting superior dialysis process control. Mineral metabolism parameters (calcium 2.32 ± 0.24 mmol/L, phosphorus 1.83 ± 0.52 mmol/L) remained within acceptable target ranges. This phenotype is characterized by intermediate dialysis adequacy (*Kt*/*V* 1.4–1.8) and moderate iron metabolism (ferritin–hemoglobulin ratio 2.0–3.0), providing balanced criteria for stable patient monitoring.

### Key discriminatory indicators among phenotypes

Machine learning feature importance analysis quantified the hierarchical contribution of clinical parameters to phenotype differentiation. Dialysis efficiency indicators demonstrated paramount discriminatory capacity, with *Kt*/*V* (*η*^2^ = 0.608) and β_2_-microglobulin reduction rate (*η*^2^ = 0.454) serving as primary phenotypic determinants. Secondary discriminators, though individually exhibiting small effect sizes, collectively captured critical pathophysiological domains: ferritin–hemoglobin ratio (*η*^2^ = 0.026) identified functional iron deficiency states characteristic of inflammatory phenotypes, while ferritin (*η*^2^ = 0.021), calcium (*η*^2^ = 0.019), and hemoglobin (*η*^2^ = 0.019) reflected mineral metabolism and erythropoietic dysfunction patterns.

**Figure 3.**
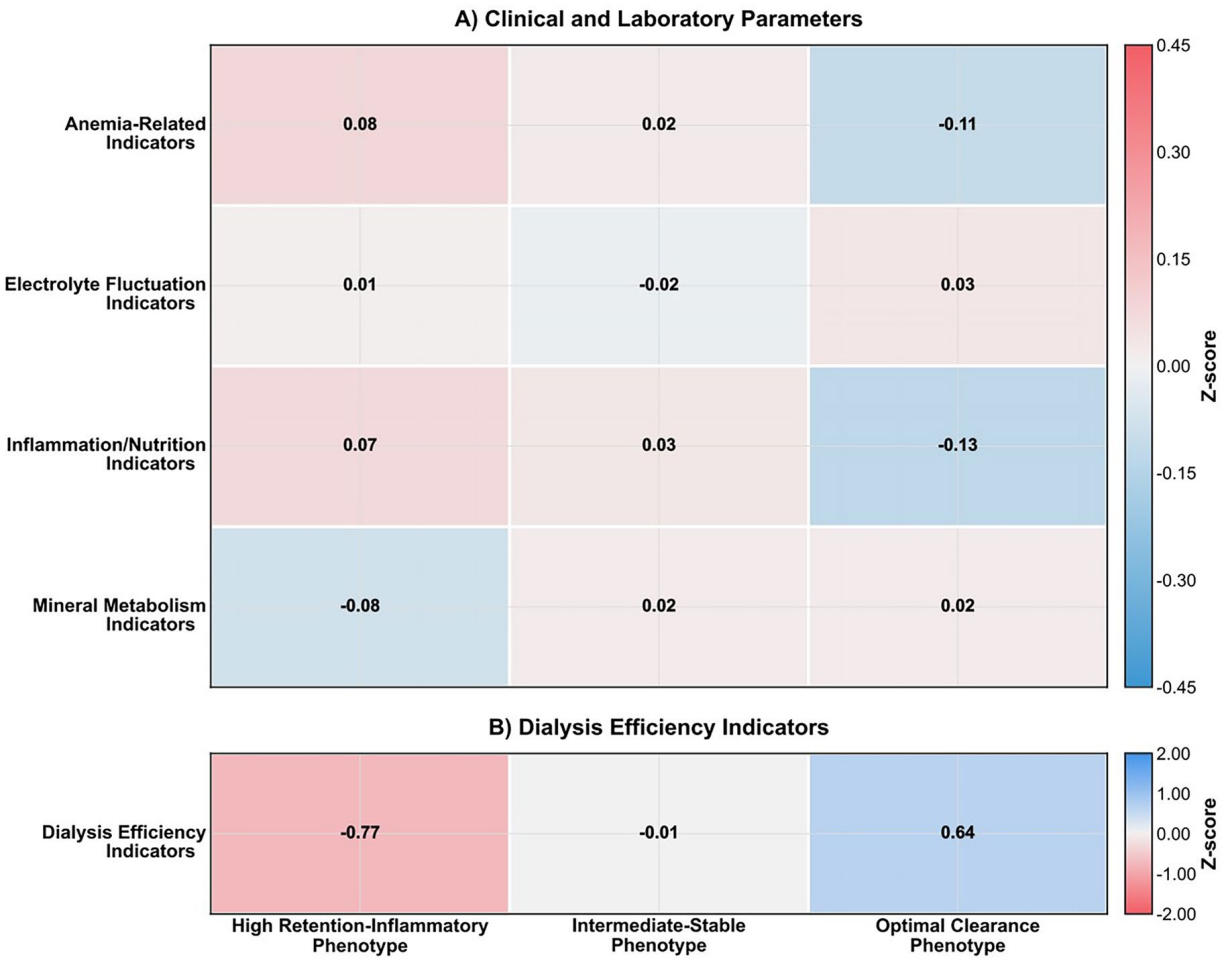
*Z*-score patterns across patient phenotypes. *Z*-score heatmap of clinical indicators across three hemodialysis patient phenotypes. (A) Clinical and laboratory parameters. (B) Dialysis efficiency indicators. Color scale: red = above average, blue = below average.

Standardized *Z*-score analysis revealed distinct phenotypic signatures consistent with underlying pathophysiology: high retention-inflammatory phenotype exhibited the ‘low clearance-high inflammation’ profile, optimal clearance demonstrated ‘high clearance-low inflammation’ characteristics, and intermediate-stable maintained population-centered values (Figure 3). These quantitative patterns establish objective criteria for precision patient stratification, transcending subjective clinical assessment through algorithmic integration of multi-dimensional biomarker interactions.

**Figure 4.**
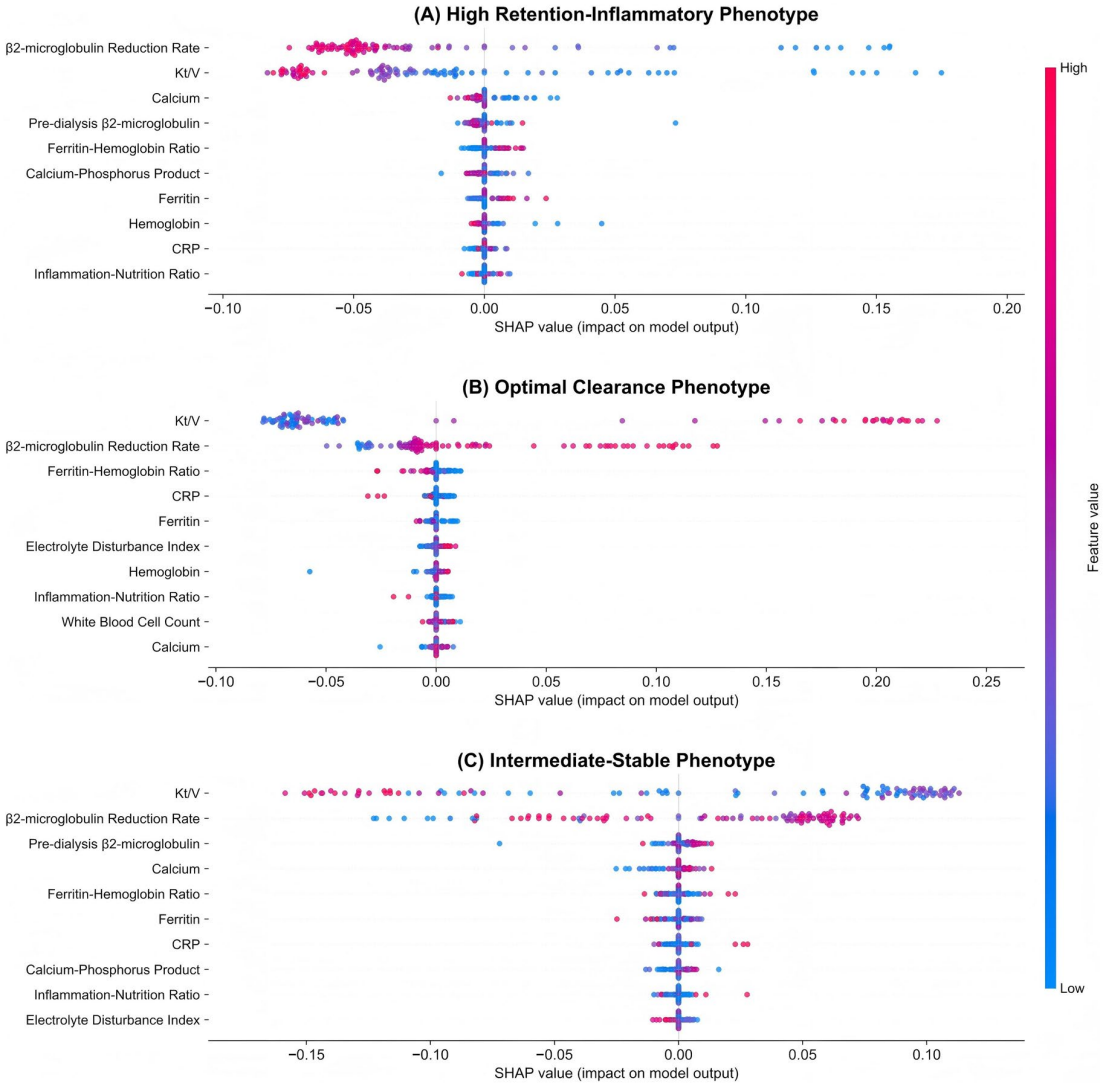
SHAP analysis of key clinical indicators across hemodialysis patient phenotypes. SHAP values for (A) high retention-inflammatory, (B) optimal clearance, and (C) intermediate-stable phenotypes show each feature’s contribution to phenotype prediction. Positive values (right) increase classification probability while negative values (left) decrease it. Features are ranked by importance; color represents feature value (red = high, blue = low). The Middle-Small Molecule Clearance Index was excluded due to its substantially higher values that would compress the scale. SHAP: SHapley Additive exPlanations.

SHAP analysis quantified feature contributions to phenotype classification through a random forest classifier, revealing distinct biomarker importance patterns across the three identified phenotypes (Figure 4).

*High retention-inflammatory phenotype* (Figure 4(A)): β_2_-microglobulin reduction rate and *Kt*/*V* demonstrated the highest discriminatory power, with negative SHAP values indicating suboptimal dialysis efficiency. Calcium exhibited negative contributions, reflecting the characteristic hypocalcemia in inflammatory states. The ferritin–hemoglobin ratio showed positive SHAP values, confirming functional iron deficiency as a phenotypic hallmark despite elevated ferritin stores.

*Optimal clearance phenotype* (Figure 4(B)): *Kt*/*V* and β_2_-microglobulin reduction rate exhibited strongly positive SHAP values, confirming superior dialysis adequacy. CRP and inflammation–nutrition ratio demonstrated negative contributions, emphasizing the role of low inflammatory burden in maintaining optimal clearance. The ferritin–hemoglobin ratio showed minimal impact, indicating efficient iron utilization.

*Intermediate-stable phenotype* (Figure 4(C)): This phenotype demonstrated balanced SHAP distributions for dialysis efficiency parameters, with *Kt*/*V* and β_2_-microglobulin reduction rate showing intermediate positive values. Pre-dialysis β_2_-microglobulin and calcium exhibited small positive contributions, while the ferritin–hemoglobin ratio remained near neutral, reflecting the transitional nature of this phenotype.

The SHAP analysis confirmed that phenotype classification extends beyond dialysis adequacy parameters to encompass iron metabolism, inflammatory status, and mineral homeostasis, providing mechanistic insights for precision therapeutic interventions.

### Value of composite indicators in phenotype identification

#### Hierarchical effect size and phenotypic discriminatory contribution of composite indicators

Composite biomarker integration demonstrated superior discriminatory capacity over univariate clinical parameters through mechanistically informed mathematical construction. The Middle-Small Molecule Clearance Index achieved large effect size (*η*^2^ = 0.77), representing optimal phenotypic discrimination through multiplicative integration of small molecule (*Kt*/*V*) and middle molecule (β_2_-microglobulin reduction rate) clearance domains. This composite metric addresses established limitations of urea kinetic modeling in quantifying cardiovascular uremic toxin accumulation.

Secondary composite indicators with small effect sizes provided clinically relevant mechanistic insights: ferritin–hemoglobin ratio (*η*^2^ = 0.026) quantified hepcidin-mediated iron sequestration during inflammatory states, while inflammation–nutrition ratio (*η*^2^ = 0.009) captured MICS through opposing acute-phase reactant dynamics.

Traditional single parameters demonstrated small individual effect sizes (ferritin *η*^2^ = 0.021, calcium *η*^2^ = 0.019, hemoglobin *η*^2^ = 0.019), collectively explaining 6% phenotypic variance. This cumulative discriminatory contribution validates multidimensional therapeutic targeting over isolated parameter optimization, supporting precision medicine approaches requiring simultaneous intervention across iron metabolism, mineral homeostasis, and inflammatory pathways.

The composite framework establishes quantitative foundations for algorithm-driven clinical decision support, enabling mechanistically informed patient stratification that transcends empirical threshold-based management through integrated pathophysiological assessment.

#### Clinical application value of the two-tier indicator framework

Our phenotyping system employs hierarchical machine learning methodology bridging clinical-research interfaces through two-tier architecture.

Tier 1 utilizes supervised classification trained on six universally available biomarkers: *Kt*/*V*, β_2_-microglobulin reduction rate (%), hemoglobin (g/L), ferritin (ng/mL), ferritin–hemoglobin ratio, and Middle Molecule Clearance Index. Parameters were selected based on pathophysiological relevance, discriminative capacity, and universal clinical accessibility per KDOQI requirements. Supervised algorithms (Random Forest, XGBoost, SVM) were trained using phenotype labels from comprehensive clustering analysis as ground truth, achieving clinically acceptable performance metrics (AUC range: 0.893–0.919, accuracy >88%) while ensuring phenotypic consistency with minimal parameter sets.

Tier 2 employs unsupervised K-means clustering utilizing comprehensive 22-parameter datasets for advanced phenotype characterization and research applications when complete biomarker panels are accessible.

The architecture operates synergistically: tier 1 enables universal clinical implementation using routine monitoring data, while tier 2 provides enhanced analytical depth with comprehensive datasets. SHAP analysis ensures feature interpretability for transparent clinical decision-making. EMR integration utilizes standardized interfaces for automated phenotype classification and therapeutic recommendation generation.

This phenotyping framework is supported by comprehensive supplementary analyses including: (1) model stability and validation assessment (Figures S1 and S2), (2) domain-specific feature discrimination analysis (Figures S3–S7), (3) multivariate feature relationships and SHAP interpretability analysis (Figures S8–S23), (4) simplified model performance evaluation and clinical scoring framework development (Figures S24–S31, Tables S1 and S2), and (5) sensitivity analysis and phenotype stability validation (Table S3, Figure S32). Complete methodological details and validation results are provided in Supplementary Materials.

## Discussion

### Machine learning identification of hemodialysis patient phenotypes

Our study employed large-scale unsupervised machine learning to quantify the heterogeneity of hemodialysis patients through comprehensive multi-dimensional indicator analysis. Our cohort analysis (*n* = 1,207) validated three phenotypic distributions – high retention-inflammatory phenotype (19.5%), optimal clearance phenotype (24.3%), and intermediate-stable phenotype (56.0%) – a classification that transcends traditional dialysis adequacy assessment. This natural stratification aligns with epidemiological observations, wherein approximately 20% of patients experience accelerated decline despite standard treatment, while approximately 25% maintain excellent stability.

The robust classification framework (Adjusted Rand Index: 0.9181) demonstrated reproducibility across demographic phenotypes, establishing a foundation for precision nephrology. Our research revealed that identical dialysis prescriptions produce significantly different metabolic responses according to patient phenotype, supporting a transition toward ‘metabolism-oriented precision medicine’ that aligns treatment with biological phenotypes rather than isolated laboratory values.

### Clinical significance of phenotype-specific biomarker patterns

The three phenotypes we identified exhibit characteristic biomarker combinations, revealing the pathophysiological heterogeneity among hemodialysis patients:

*Iron metabolism heterogeneity*: The high retention-inflammatory phenotype demonstrated elevated ferritin–hemoglobin ratios, reflecting inflammation-driven functional iron deficiency. This may be associated with hepcidin upregulation leading to impaired iron release [33], explaining the clinical paradox of ‘adequate iron stores with poor EPO response.’ This composite indicator may be superior to single iron parameters in assessing iron utilization impairment in inflammatory states [34], providing new perspectives for anemia management.

The elevated ferritin levels with concurrent hemoglobin deficiency in the high retention-inflammatory phenotype reflect hepcidin-mediated iron sequestration. Interleukin-6, elevated in uremic patients, activates the JAK2/STAT3 signaling pathway, leading to transcriptional upregulation of hepcidin through direct STAT3 binding to the hepcidin promoter [35]. Recent structural studies demonstrate that hepcidin binding induces ferroportin conformational changes, triggering degradation through both ubiquitin–proteasome and lysosomal pathways [36,37]. Contemporary clinical studies demonstrate elevated hepcidin levels in CKD patients, with levels correlating with markers of iron metabolism and systemic inflammation [37].

Uremic toxins including β_2_-microglobulin and indoxyl sulfate perpetuate this inflammatory cascade. β_2_-microglobulin stimulates proinflammatory cytokine production (IL-6, TNF-α) in macrophages [38], while indoxyl sulfate enhances macrophage response to inflammatory stimuli and increases oxidative stress [39,40]. This creates a pathological feedback loop where kidney dysfunction exacerbates iron dysregulation, contributing to erythropoietin resistance and anemia progression [21].

*Mineral metabolism abnormalities*: The lower calcium levels in the high retention-inflammatory phenotype align with mechanisms of inflammatory factor inhibition of vitamin D metabolism [41]. This mineral metabolism disorder affects both bone health and potentially increases cardiovascular risk [42], supporting the importance of the bone-cardiovascular axis in risk stratification.

*Dialysis efficiency and inflammation relationship*: The low inflammation levels in the optimal clearance phenotype positively correlate with high dialysis efficiency, echoing the theory of mutual influence between uremic toxin clearance and inflammatory status [43]. Efficient clearance may mitigate inflammatory responses, while low inflammation levels facilitate maintenance of optimal vascular access function, forming a beneficial cycle.

*Foundations for individualized treatment*: The biomarker characteristics of different phenotypes predict variations in treatment response. The high retention-inflammatory phenotype may benefit from intensified dialysis strategies and targeted anti-inflammatory interventions, while the intermediate-stable phenotype requires regular monitoring to prevent transition to high-risk phenotypes.

This molecular phenotype stratification provides a theoretical foundation for precision dialysis therapy, advancing hemodialysis from standardized management toward biomarker-guided individualized intervention.

### Comparisons with previous research

This investigation represents a paradigmatic advancement in hemodialysis phenotyping methodology, addressing critical limitations of prior clustering studies through scale, innovation, and clinical translation frameworks. Our cohort (*n* = 1,207) provides order-of-magnitude enhancement in statistical power over Yu et al. (*n* = 167) [9] and Komaru et al. (*n* = 101) [12], enabling robust detection of clinically relevant phenotypic distinctions previously obscured by inadequate sample sizes.

Methodological innovations distinguish this work from existing literature. Our novel two-tier phenotyping architecture – integrating comprehensive 22-parameter unsupervised clustering with streamlined six-parameter supervised classification – addresses the fundamental clinical translation gap between research-grade analytics and routine clinical implementation. This hierarchical approach achieves exceptional performance (AUC 0.893–0.919, accuracy >88%) while maintaining phenotypic consistency across parameter sets, representing unprecedented advancement over single-tier clustering methodologies employed in prior studies.

Composite biomarker development constitutes another significant innovation. The Middle-Small Molecule Clearance Index and ferritin–hemoglobin ratio demonstrate mechanistically informed integration of pathophysiological domains, transcending univariate approaches utilized in previous investigations. These indices capture complex interactions between clearance efficiency, inflammation, and iron metabolism inadequately assessed by traditional single-parameter classifications.

Acknowledged limitations include cross-sectional design precluding temporal phenotype stability assessment and absence of hard endpoint validation. However, our rigorous internal validation methodology (ARI = 0.9181) and clinical implementation framework establish robust foundations for prospective validation studies required for precision nephrology translation.

### Clinical implementation pathway for precision dialysis management

#### Clinical value proposition for algorithmic patient stratification

While experienced hemodialysis clinicians routinely evaluate the clinical parameters examined in this investigation, our algorithmic classification framework provides distinct quantitative advantages over conventional subjective assessment methodologies, establishing a paradigm shift from experience-based clinical assessment toward data-driven precision medicine approaches. The machine learning approach addresses fundamental clinical practice challenges through: (1) objective standardization that eliminates inter-provider assessment variability inherent in subjective clinical evaluation; (2) systematic multidimensional integration of 22-parameter interactions that exceed typical human cognitive bandwidth for complex pattern recognition; (3) reproducible classification criteria ensuring consistent patient stratification across diverse clinical settings and temporal assessments; (4) enhanced sensitivity for detecting subtle metabolic transitions preceding clinically apparent deterioration through mathematical optimization of discriminatory thresholds.

#### Evidence-based clinical interventions for phenotype classification

The following phenotype-guided management strategies represent evidence-based hypotheses derived from established pathophysiological principles and KDOQI guidelines, requiring randomized controlled trial validation before clinical implementation.

Based on the identified phenotypic characteristics, we propose a phenotype-guided clinical decision framework. First, management of high retention-inflammatory phenotype patients should focus on optimizing dialysis adequacy, including consideration of extended dialysis time or increased frequency to achieve the recommended *Kt*/*V* target (≥1.4). Due to potential functional iron deficiency, a graded iron supplementation strategy is recommended, accompanied by comprehensive assessment of CRP and iron metabolism indicators, avoiding decisions based solely on single ferritin thresholds. For patients exhibiting high inflammation levels, ESA responsiveness may be reduced, necessitating adjusted dosage expectations and close monitoring of hemoglobin change rates.

In contrast, patients with the optimal clearance phenotype can maintain current dialysis parameters, while remaining vigilant for potential risks of excessive clearance and monitoring iron overload, particularly in low-weight patients. The intermediate-stable phenotype should follow standard dialysis protocols, while establishing regular monitoring mechanisms to identify early signals of transition to high-risk phenotypes, focusing on trends in inflammatory markers and clearance efficiency indicators.

Clinical implementation requires stratified monitoring with quarterly comprehensive phenotype assessments, monthly evaluation of key indicators (dialysis adequacy, inflammatory markers, anemia parameters), and enhanced protocols for unstable phenotypes. By establishing an early warning threshold system, clinicians can intervene before significant changes in biomarker patterns occur, proactively adjusting treatment regimens.

#### Clinical feasibility and automated EMR integration framework

The classification system demonstrates exceptional feasibility for automated EMR implementation through exclusive utilization of standard hemodialysis monitoring parameters universally mandated by KDOQI guidelines and CMS reporting requirements. Implementation requires only routine laboratory data already collected during standard-of-care dialysis monitoring (complete blood count, comprehensive metabolic panel, dialysis adequacy measurements, inflammatory markers) without additional testing burden or workflow modifications. The computational efficiency of K-means clustering algorithms enables real-time patient classification through existing EMR infrastructures, providing immediate automated phenotype identification that enhances clinical decision-making through objective, evidence-based patient stratification protocols. The preliminary HPCS scoring framework complements these automated approaches by providing transparent, calculable decision boundaries, though multicenter validation across diverse healthcare settings remains essential before routine clinical adoption.

The automated classification capability addresses practical clinical challenges including objective documentation for quality improvement initiatives, standardized communication between healthcare providers, and consistent patient stratification for clinical research protocols. This systematic approach complements clinical intuition with quantitative assessment tools that enhance traditional patient care paradigms.

The proposed stratified management system requires no additional specialized testing. Implementation strategies can follow a hierarchical medical institution approach: tertiary hospitals first establish phenotype identification systems and treatment protocols, subsequently promoting standardized processes to secondary hospitals and dialysis centers. Information system support is crucial; we recommend developing automated phenotype identification algorithms and decision support tools to assist clinicians in individualized treatment decisions.

This implementation framework, through precise phenotyping-guided interventions, can reduce inefficient use of iron supplements and ESAs, lower complication risks due to inappropriate treatment, and facilitate the translation of precision dialysis management from research to routine clinical practice.

#### Therapeutic validation requirements

The proposed phenotype-specific interventions require rigorous randomized controlled trial validation before clinical implementation. Our study establishes the fundamental prerequisite for precision medicine trials – identification of clinically distinct patient subgroups with characteristic biomarker signatures. Randomized controlled trials of phenotype-directed versus standard care are essential for future studies, focusing on standardized intervention protocols and clinical efficacy assessment.

### Study limitations

Our study has several limitations requiring consideration. While our cross-sectional design precludes definitive assessment of longitudinal dynamics and causal relationships with clinical outcomes, preliminary analysis of 349 patients with 3-month follow-up demonstrated substantial phenotype stability (*κ* = 0.686, 79.1% retention), providing initial support for temporal consistency. The robust phenotyping framework (ARI = 0.9181) provides essential methodological infrastructure for future outcome validation. The identified phenotypes incorporate biomarkers with established prognostic significance (β_2_-microglobulin, CRP/albumin ratio), suggesting biological plausibility for clinical relevance, though prospective studies with extended follow-up remain necessary to establish transition patterns and hard endpoint associations.

Single-center recruitment from a Shanghai-based tertiary hospital introduces potential regional biases regarding dialysis prescription practices, demographic characteristics, and clinical management protocols, potentially limiting phenotype generalizability. While this approach enabled standardized laboratory methodologies and uniform protocols essential for robust phenotype discovery, validation across diverse healthcare systems and populations remains necessary. Our phenotype distributions (19.5%, 24.3%, 56.0%) align with international epidemiological patterns, and the classification framework reflects universal pathophysiological principles, supporting broader applicability.

Implementation across different healthcare systems will require addressing specific technical and operational challenges. Laboratory standardization represents the primary challenge, as β_2_-microglobulin measurement protocols and composite indicator calculations must maintain consistency across institutions. Population-specific adaptations may be necessary, as different ethnic compositions, age distributions, and comorbidity patterns could influence phenotype prevalence and biomarker thresholds. Technical infrastructure variations, including EMR data formats and extraction capabilities, will require flexible implementation protocols. Systematic adaptation strategies should include: standardized laboratory protocols for key biomarkers, population-specific threshold validation, and modular EMR integration frameworks that accommodate diverse health information systems while preserving core phenotypic discrimination capabilities.

The association between identified phenotypes and hard clinical endpoints remains unvalidated, representing a fundamental limitation of cross-sectional phenotype discovery research. While our phenotypes demonstrate robust statistical stability (Adjusted Rand Index = 0.9181) and pathophysiological coherence, their prognostic significance for mortality, cardiovascular events, and hospitalization rates requires prospective validation. This limitation is inherent to phenotype discovery methodology, which constitutes the essential first step toward precision medicine development. Future studies should establish prospective cohorts to validate phenotype associations with 2-year mortality and cardiovascular complications, as demonstrated in established dialysis outcome research. Similarly, while the preliminary HPCS demonstrates promising internal validation performance, it requires substantial external validation across diverse healthcare settings and patient populations to establish generalizability and clinical utility before implementation in routine practice.

Dynamic biomarker fluctuation represents a fundamental methodological limitation. Single-timepoint assessment provides discrete snapshots of evolving metabolic states, potentially affected by circadian variations, weekly inflammatory periodicity, and interdialytic behavioral factors. While composite indicator construction and standardized protocols minimize temporal bias, this limitation may introduce transient classification uncertainty. The framework architecture enables repeated application for dynamic phenotype reassessment and longitudinal monitoring.

Our sensitivity analyses demonstrated that dialysis prescriptions were homogeneous across phenotypes and explained <15% of biomarker variance. However, medication data (ESA dosing, iron supplementation, active vitamin D) were unavailable for adjustment. This reflects both a limitation and our study’s real-world applicability – the phenotypes capture metabolic states as they exist under standard care, integrating biological and treatment responses.

The sensitivity of K-means algorithms to outliers poses a potential limitation; despite our application of Isolation Forest for preprocessing, boundary cases with classification uncertainty may still exist.

### Clinical translation pathway and research prospects

#### Multi-center research framework for phenotype validation

This phenotype classification represents foundational work requiring systematic prognostic validation through multicenter prospective research. Initial validation cohorts (*n* ≈ 3,000) should prioritize hard endpoint analysis including 2-year all-cause mortality, cardiovascular events, and hospitalization rates, establishing the prognostic significance of our phenotyping framework and cardiovascular risk stratification protocols before advancing to therapeutic intervention trials.

These cohorts should be established in five regional dialysis centers to assess phenotype distribution and biomarker pattern stability. Subsequently, parallel-group randomized controlled trials should compare phenotype-guided management with standard care protocols, establishing 12-month composite primary endpoints (all-cause hospitalization, dialysis-related complications, and dialysis adequacy achievement rates) and key secondary endpoints (anemia control, symptom burden, and health-related quality of life); the third phase should develop phenotype transition early warning models through routine clinical data and selective biomarkers to support clinical decision-making. Biospecimen repositories can be established in capable centers to facilitate in-depth research on phenotype molecular characteristics.

#### Multi-dimensional integration prospects for dialysis precision medicine

Technological infrastructure should prioritize the construction of interoperable clinical decision support platforms, enabling real-time biomarker analysis and individualized treatment recommendations. AI-driven systems can integrate natural language processing for clinical record analysis, while federated learning facilitates cross-institutional data utilization and model optimization. Multi-omics integration represents future developmental directions, including microbiome analysis identifying gut–kidney axis interactions, metabolomics characterizing phenotype-specific uremic toxin profiles, and pharmacogenomics optimizing treatment response prediction. Noninvasive sensing technologies combined with machine learning may enable transition from point biomarker detection to continuous metabolic monitoring. Interdisciplinary collaboration networks will promote translation of phenotype research findings into clinical practice, establishing evidence-based phenotype-guided treatment protocols. This progressive development pathway aims to transform hemodialysis from standardized procedures to individualized treatment strategies. The immediate research priority remains establishing phenotype–mortality associations through prospective cohort studies, followed by development of automated clinical classification tools that translate our validated phenotyping framework into actionable precision medicine interventions, ultimately optimizing both clinical outcomes and healthcare resource utilization.

## Conclusions

This investigation represents the largest and most methodologically rigorous machine learning-based phenotyping study in maintenance hemodialysis populations, successfully identifying three clinically distinct patient phenotypes through unsupervised clustering analysis of 1,207 subjects using 22 multidimensional clinical indicators. The identified phenotypes – high retention-inflammatory (19.5%), optimal clearance (24.3%), and intermediate-stable (56.0%) – demonstrate exceptional classification robustness (Adjusted Rand Index = 0.9181) and pathophysiologically coherent biomarker signatures.

The study establishes significant methodological advances through development of a novel two-tier phenotyping architecture that bridges the critical gap between comprehensive research analytics and clinical implementation feasibility. The validated six-parameter supervised learning framework achieves clinically acceptable performance metrics (AUC range: 0.893–0.919) while maintaining phenotypic consistency with the comprehensive clustering approach, enabling immediate integration into existing electronic health record systems using standard KDOQI monitoring parameters.

Novel composite biomarkers, particularly the Middle-Small Molecule Clearance Index and ferritin–hemoglobin ratio, demonstrate superior discriminatory capacity compared to conventional univariate clinical parameters, capturing complex pathophysiological interactions between uremic toxin clearance, inflammatory status, and iron metabolism dynamics. These mechanistically informed indices provide objective, quantitative foundations for precision therapeutic decision-making that transcend traditional empirical threshold-based management approaches.

The phenotype-specific biomarker patterns support evidence-based stratified management protocols, offering a systematic framework for individualized therapeutic interventions targeting distinct pathophysiological mechanisms. This machine learning-driven classification methodology establishes robust scientific foundations for transitioning hemodialysis care from standardized protocols toward precision nephrology applications, with potential to enhance clinical outcomes through objective patient stratification and targeted therapeutic optimization. Future prospective validation studies are essential to establish prognostic significance and therapeutic efficacy of phenotype-directed interventions.

## Supplementary Material

Supplementary Material_20.jpg

Supplementary Material_25.jpg

Supplementary Material_30.jpg

Supplementary Material_15.jpg

Supplementary Material_08.jpg

Supplementary Material_31.jpg

Supplementary Material_24.jpg

Supplementary Material_10.jpg

Supplementary Material_22.jpg

Supplementary Material_06.jpg

Supplementary Material_28.jpg

Supplementary Material_34.jpg

Supplementary Material_03.jpg

Supplementary Material_09.jpg

Supplementary Material_02.jpg

Supplementary Material_04.jpg

Supplementary Material_05.jpg

Supplementary Material_11.jpg

Supplementary Material_26.jpg

Supplementary Material_01.jpg

Supplementary Material_12.jpg

Supplementary Material_14.jpg

Supplementary Material_33.jpg

Supplementary Material_16.jpg

Supplementary Material_37.jpg

Supplementary Material_32.jpg

Supplementary Material_35.jpg

Supplementary Material_21.jpg

Supplementary Material_23.jpg

Supplementary Material Table of Contents.docx

Supplementary Material_29.jpg

Supplementary Material_36.jpg

Supplementary Material_17.jpg

Supplementary Material_18.jpg

Supplementary Material_19.jpg

Supplementary Material_07.jpg

Supplementary Material_27.jpg

Supplementary Material_13.jpg
