# Supplementary figures and images for: Data-driven cluster analysis identifies three clinical phenotypes in hemodialysis patients
Source: Ren Fail. 2025 Nov 20;47(1):2588961. doi: 10.1080/0886022X.2025.2588961 (PMC12636659; doi:10.1080/0886022X.2025.2588961)

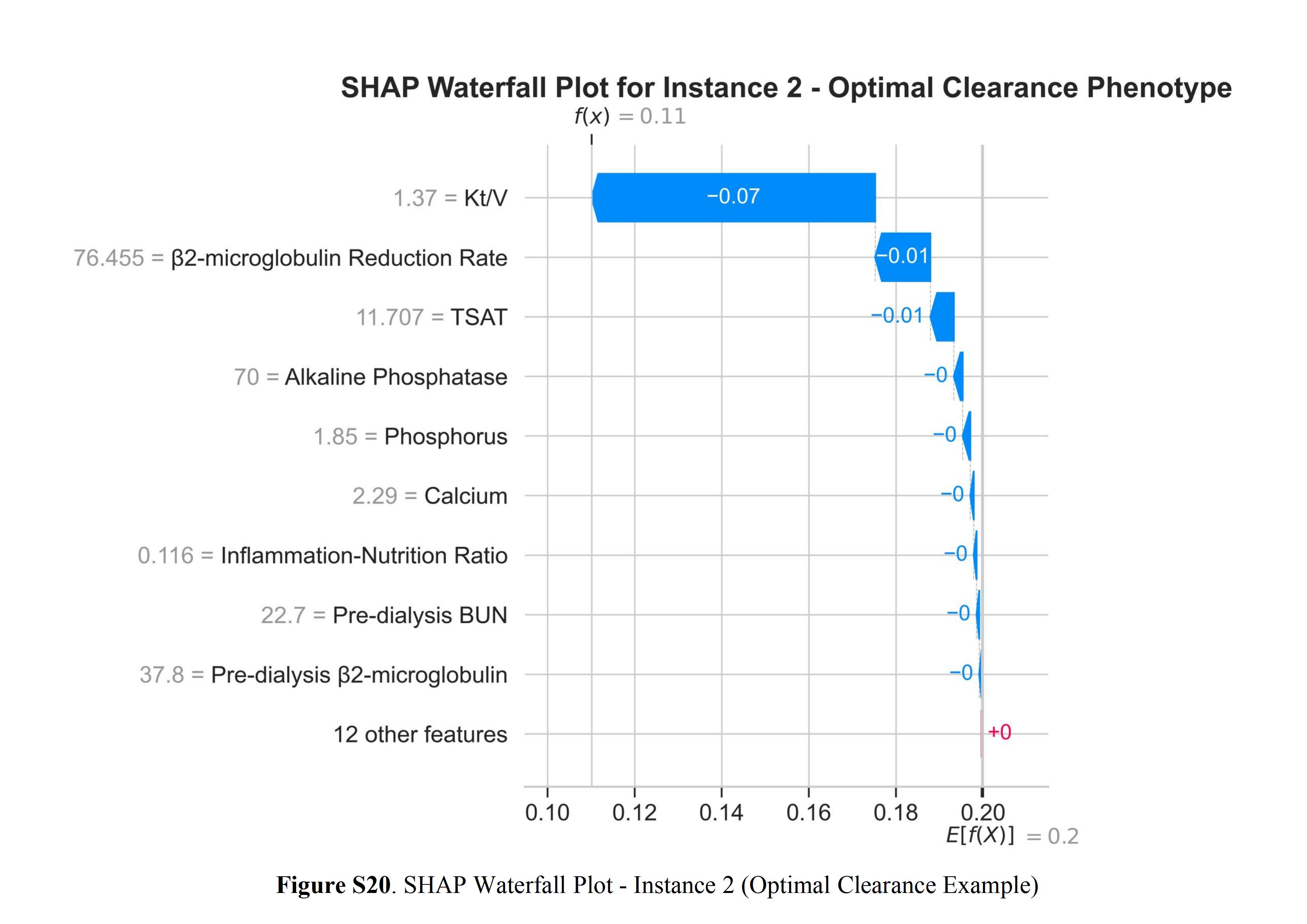

Supplement: Supplementary Material_20.jpg [file IRNF_A_2588961_SM0967.jpg]

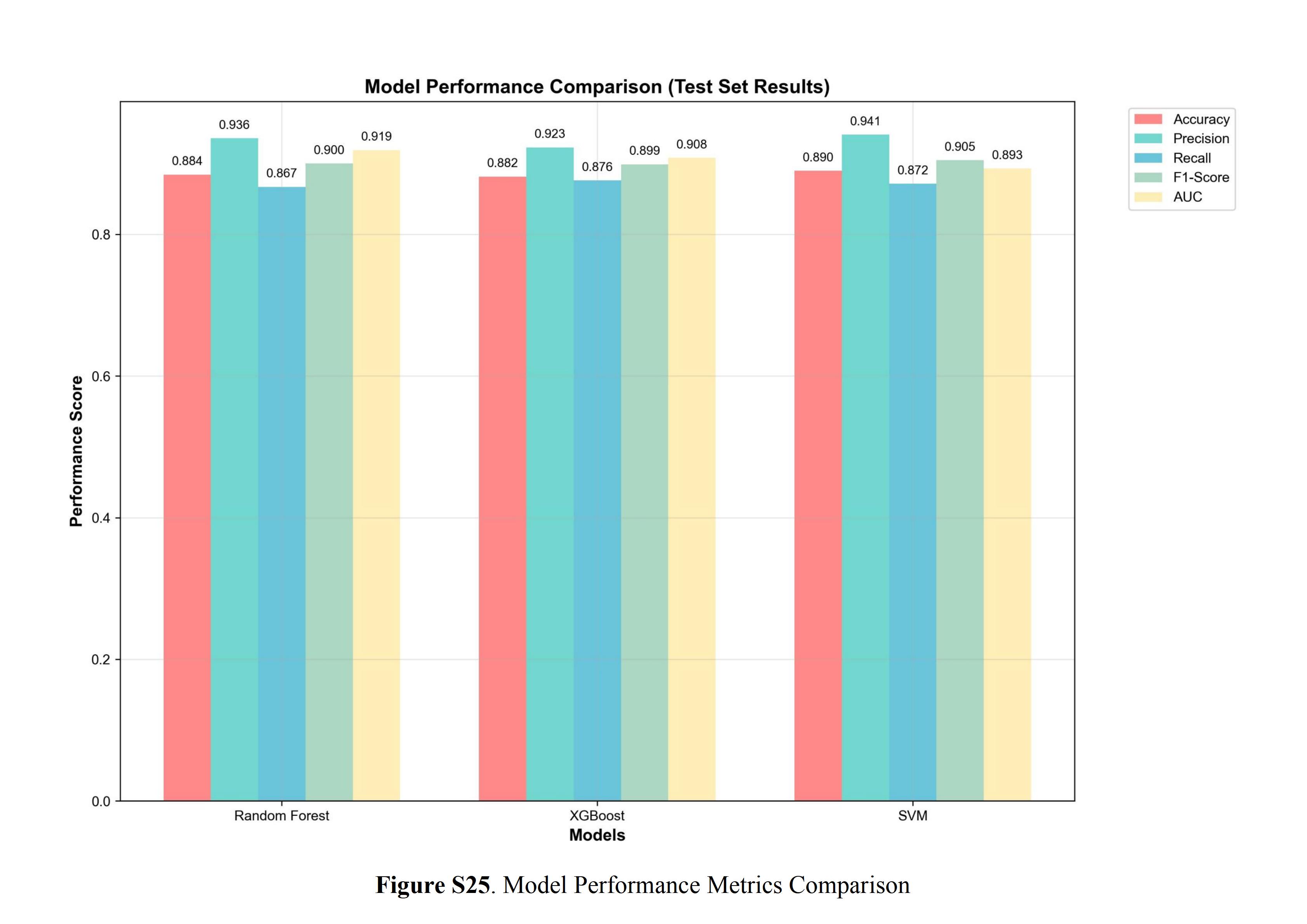

Supplement: Supplementary Material_25.jpg [file IRNF_A_2588961_SM0966.jpg]

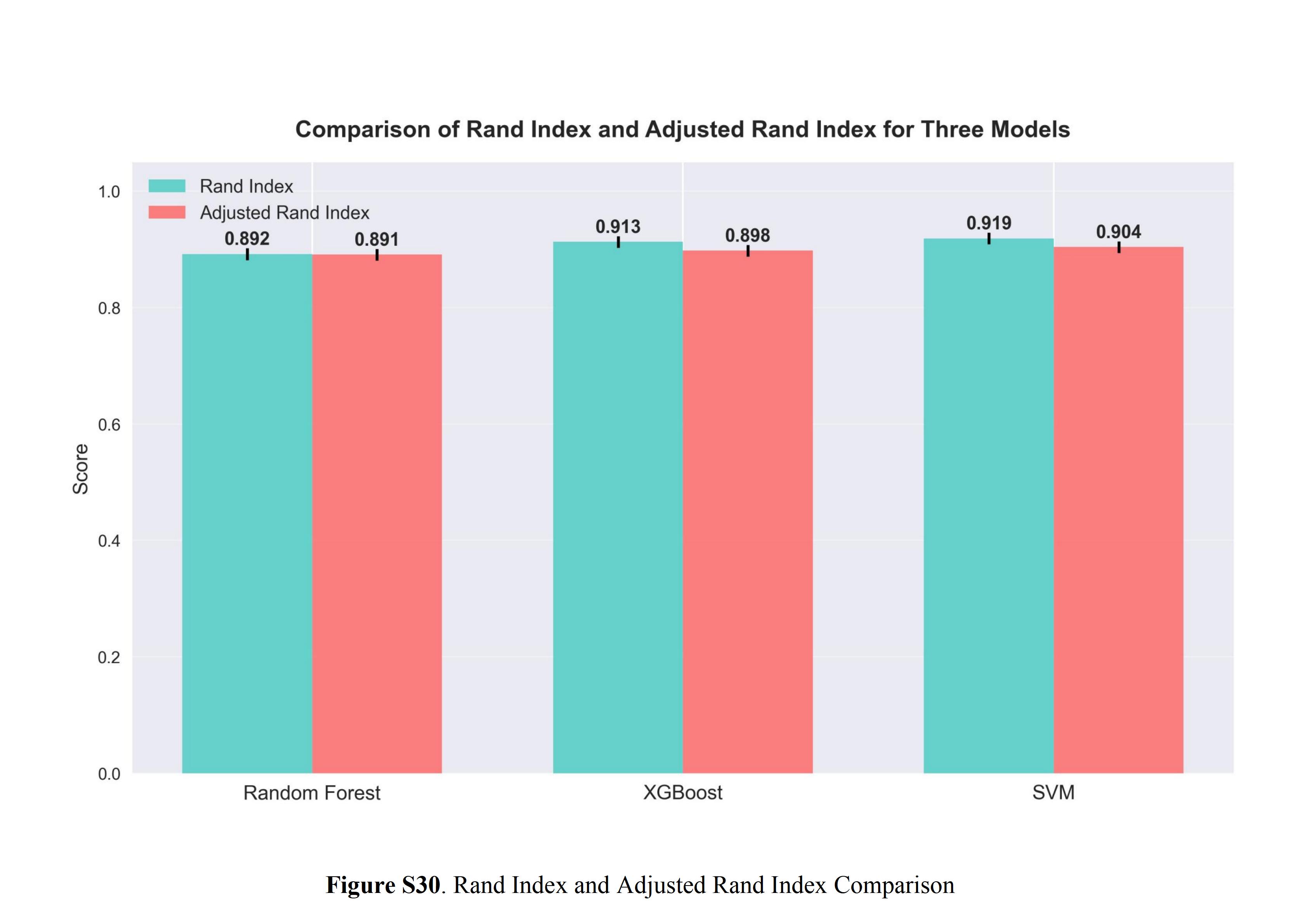

Supplement: Supplementary Material_30.jpg [file IRNF_A_2588961_SM0965.jpg]

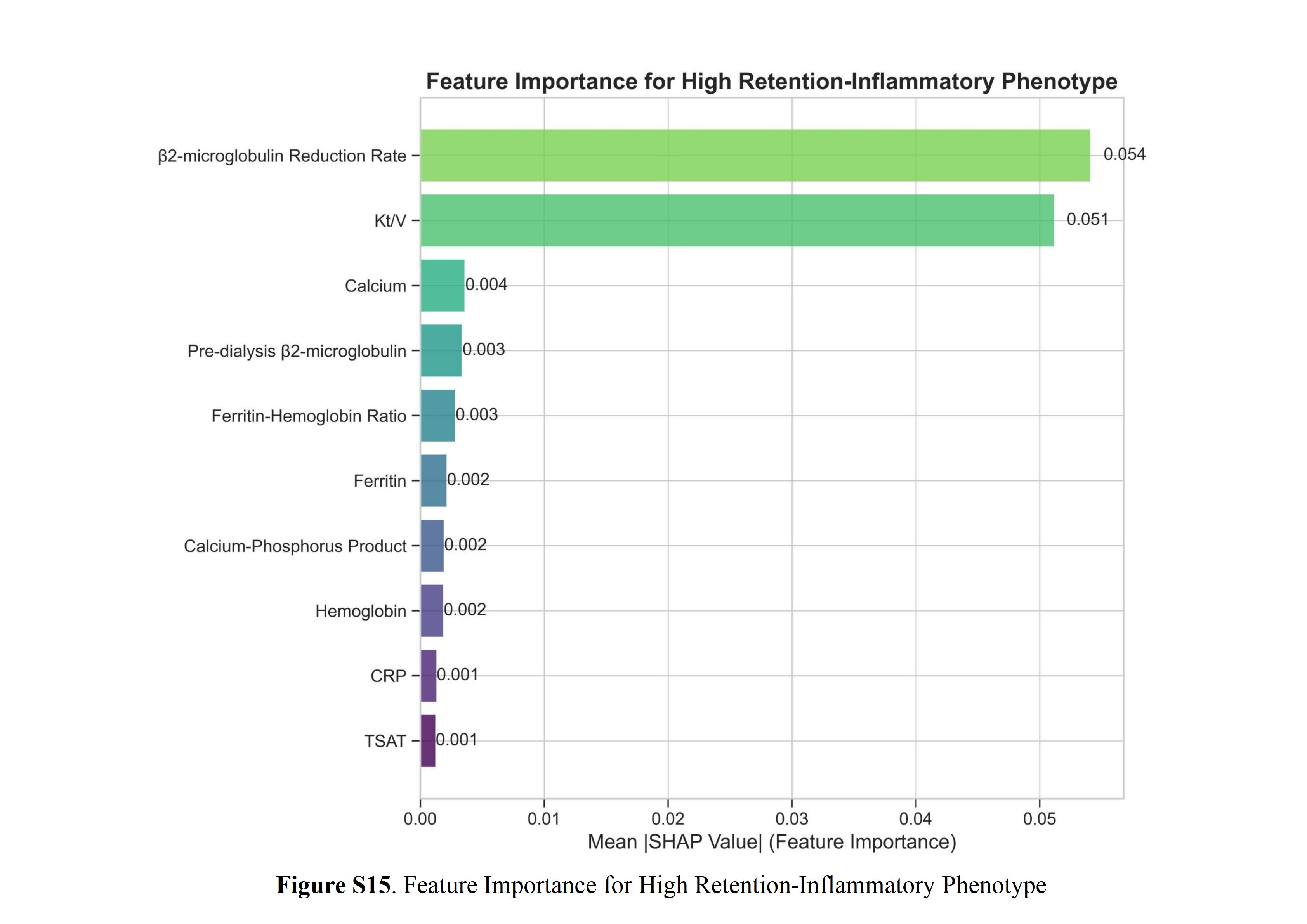

Supplement: Supplementary Material_15.jpg [file IRNF_A_2588961_SM0964.jpg]

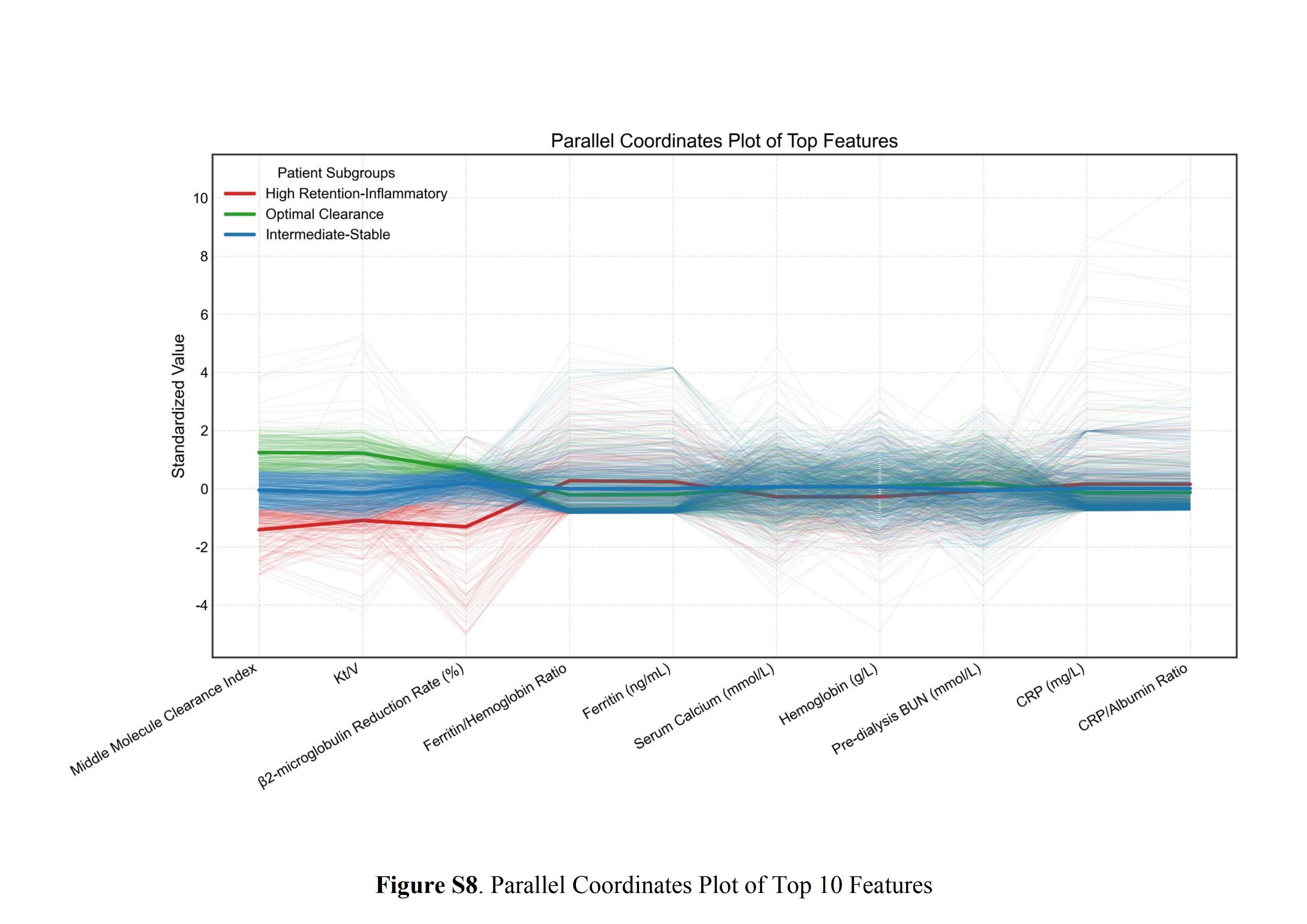

Supplement: Supplementary Material_08.jpg [file IRNF_A_2588961_SM0963.jpg]

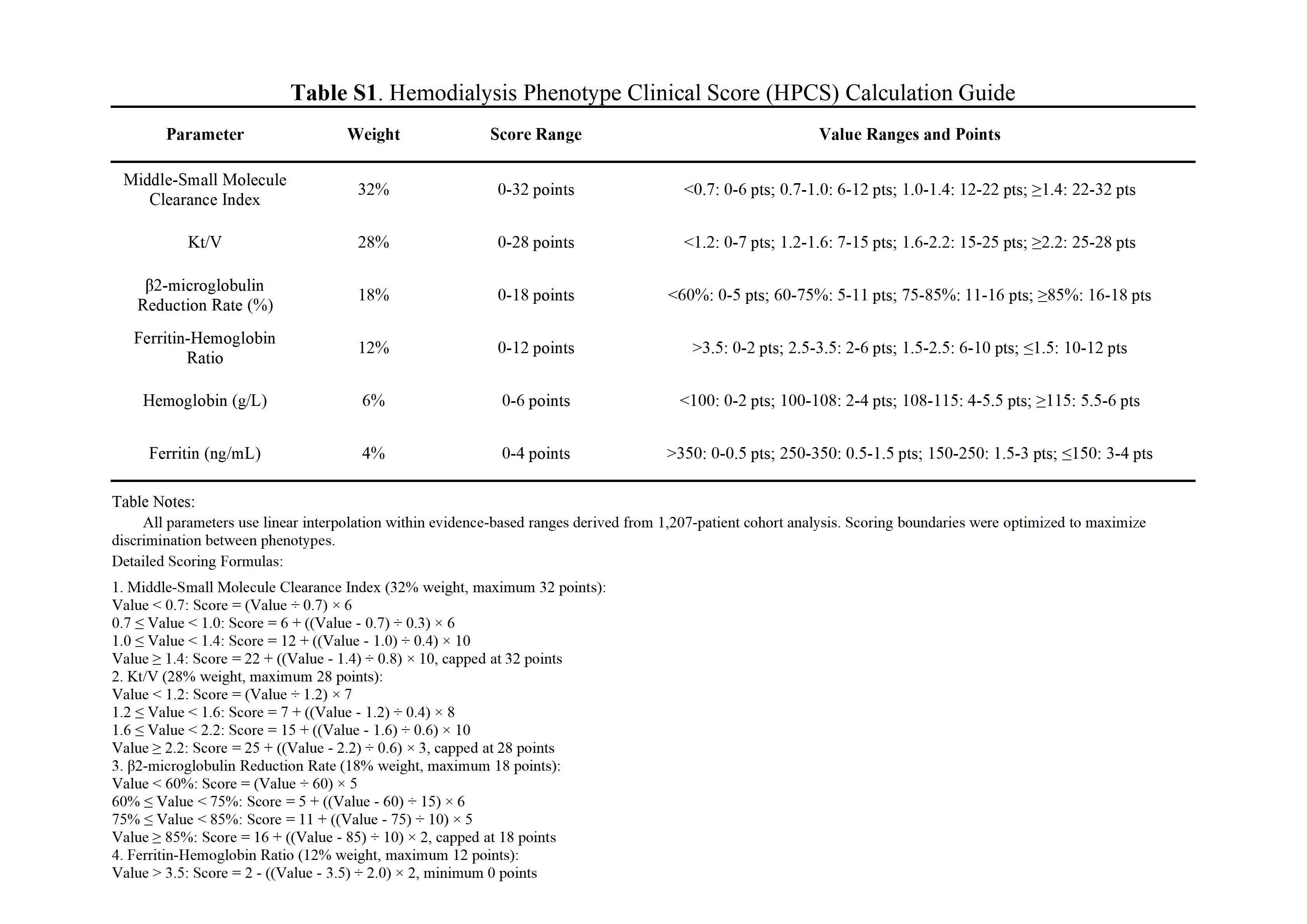

Supplement: Supplementary Material_31.jpg [file IRNF_A_2588961_SM0962.jpg]

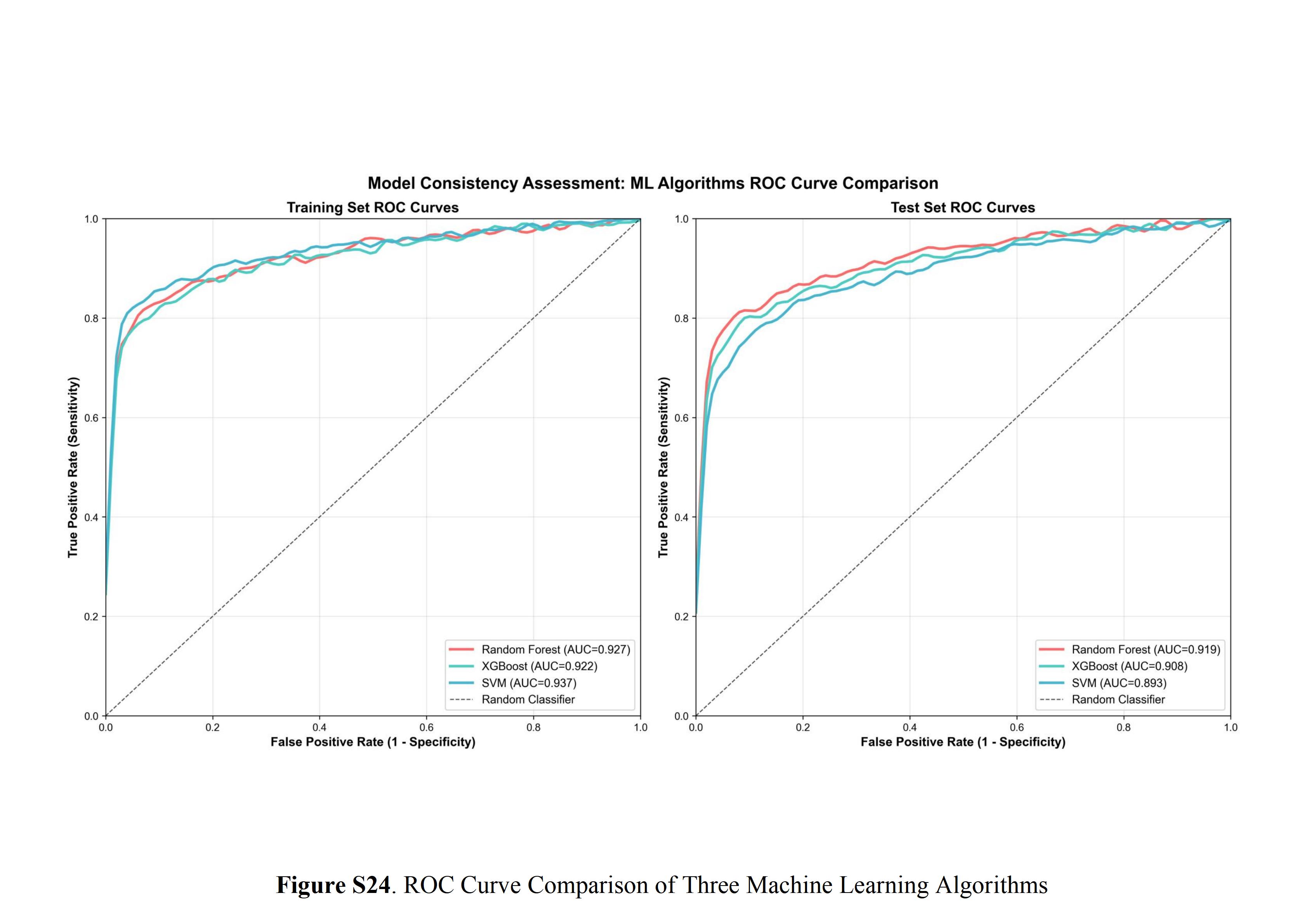

Supplement: Supplementary Material_24.jpg [file IRNF_A_2588961_SM0961.jpg]

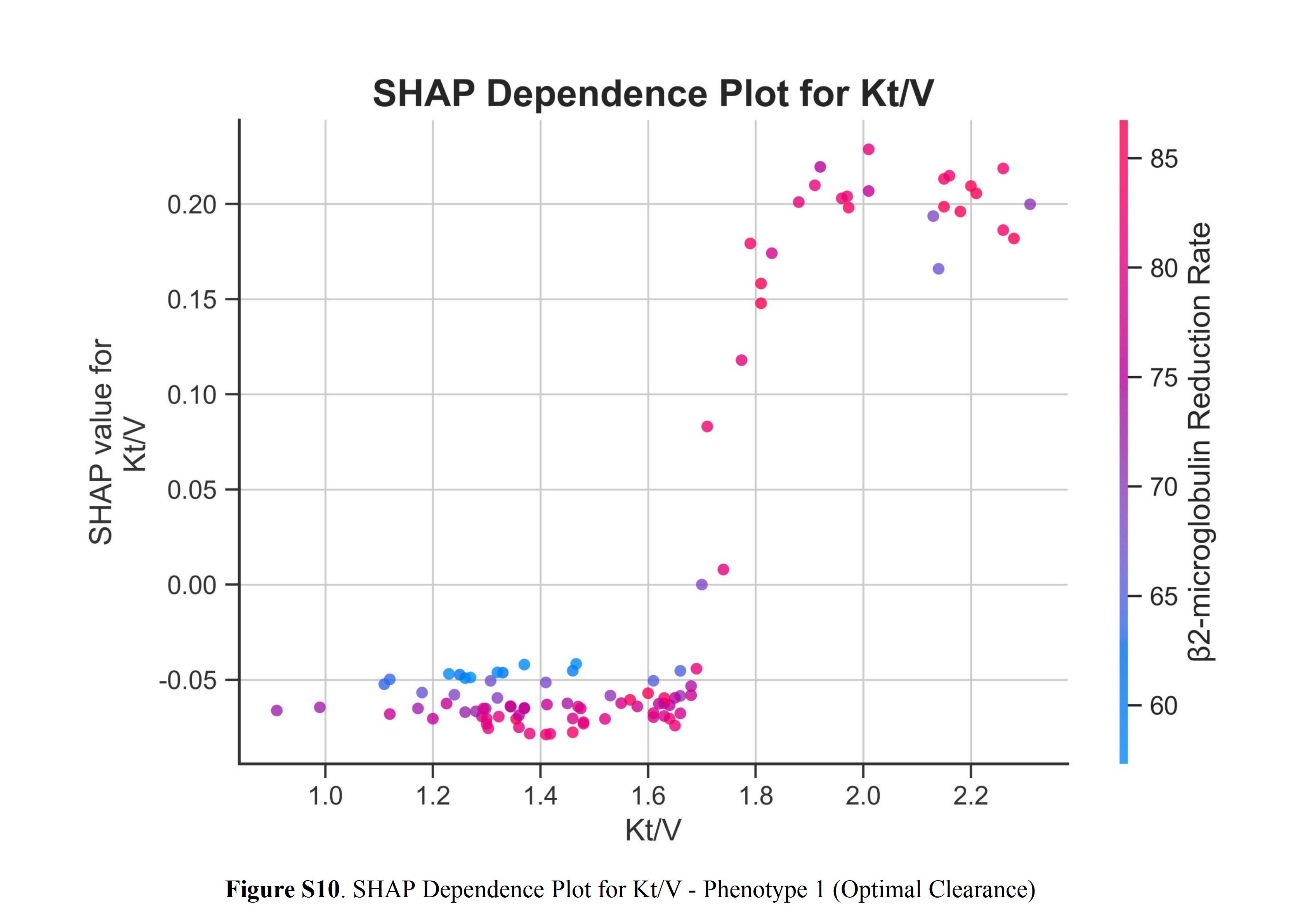

Supplement: Supplementary Material_10.jpg [file IRNF_A_2588961_SM0960.jpg]

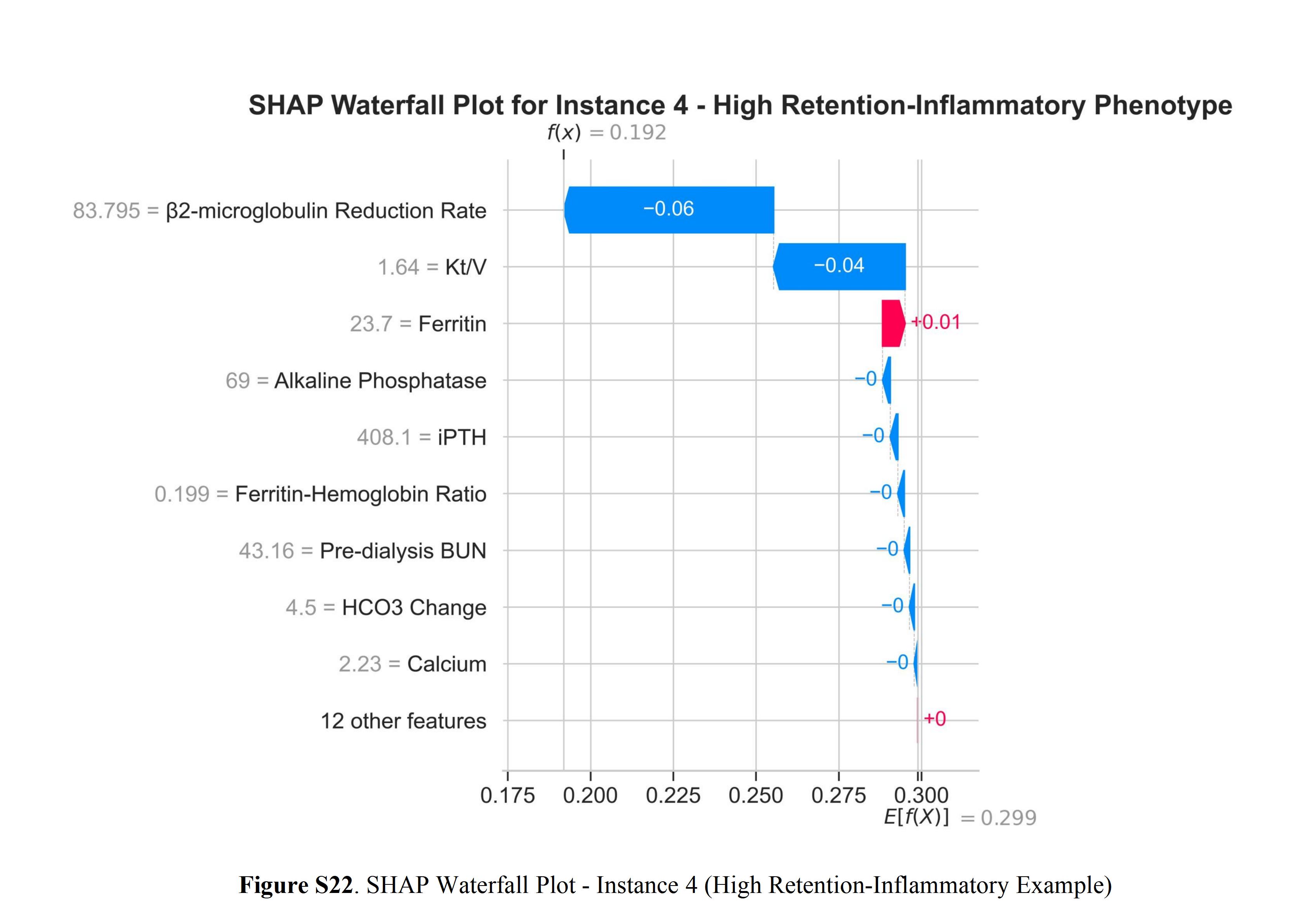

Supplement: Supplementary Material_22.jpg [file IRNF_A_2588961_SM0959.jpg]

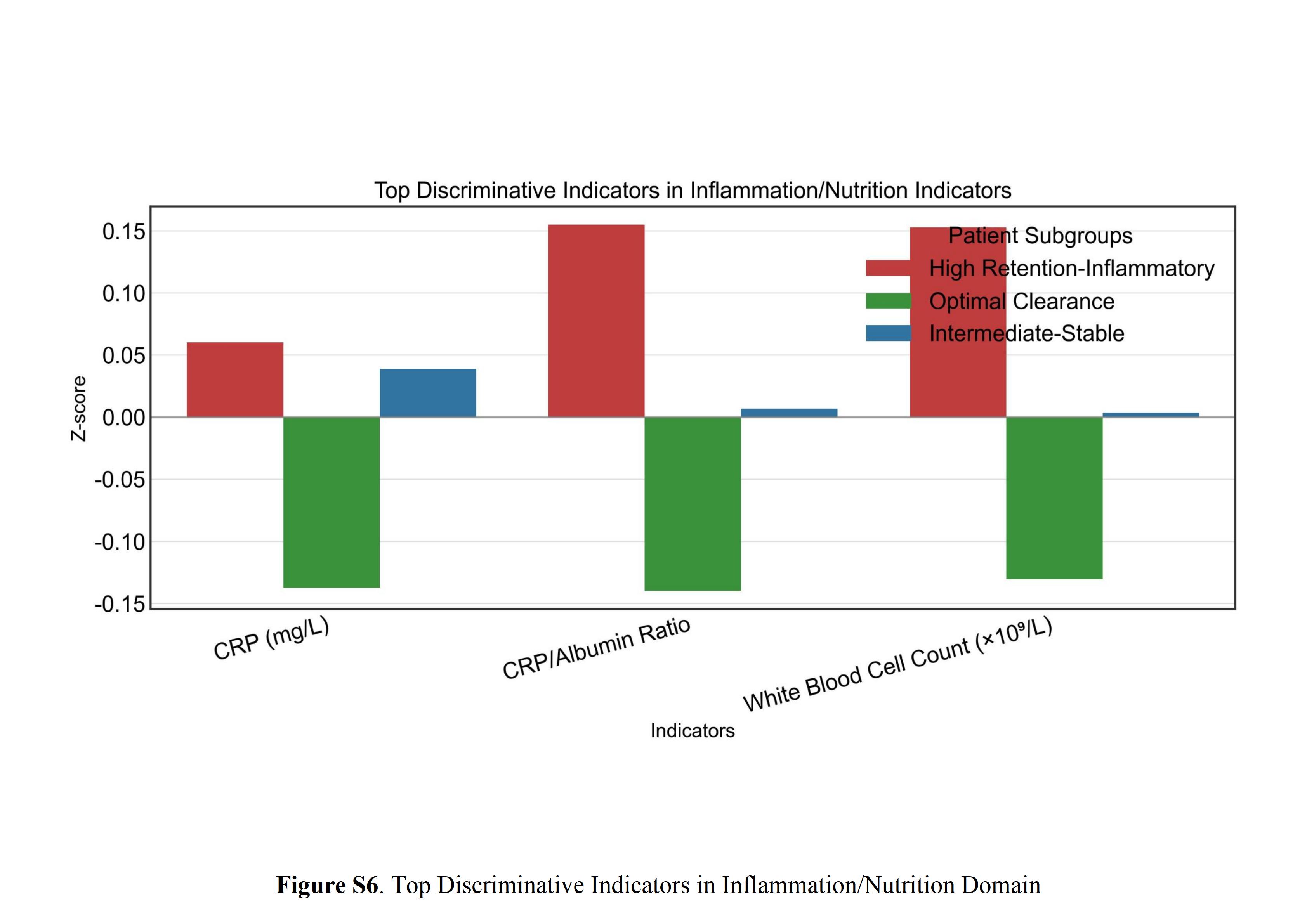

Supplement: Supplementary Material_06.jpg [file IRNF_A_2588961_SM0958.jpg]

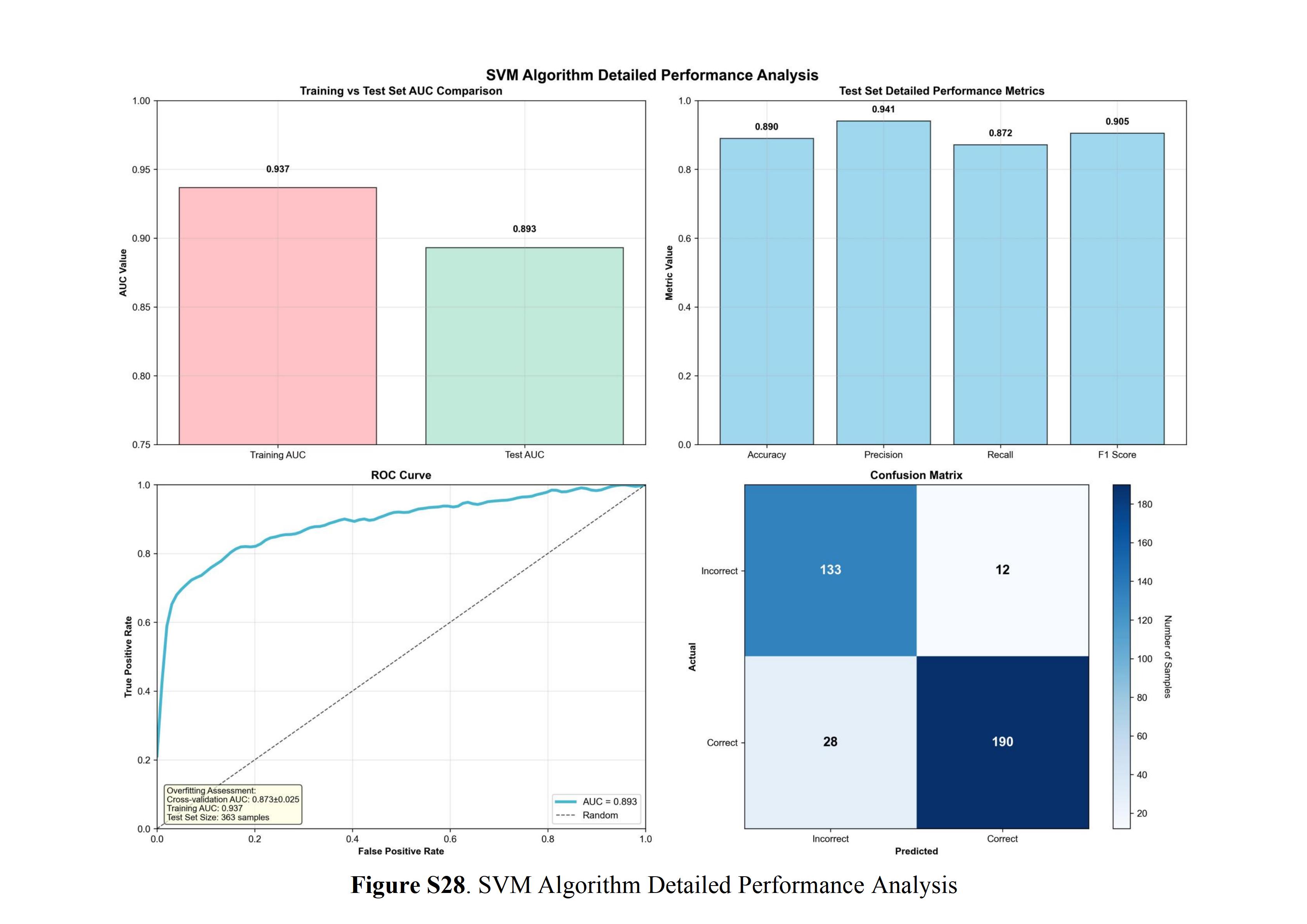

Supplement: Supplementary Material_28.jpg [file IRNF_A_2588961_SM0957.jpg]

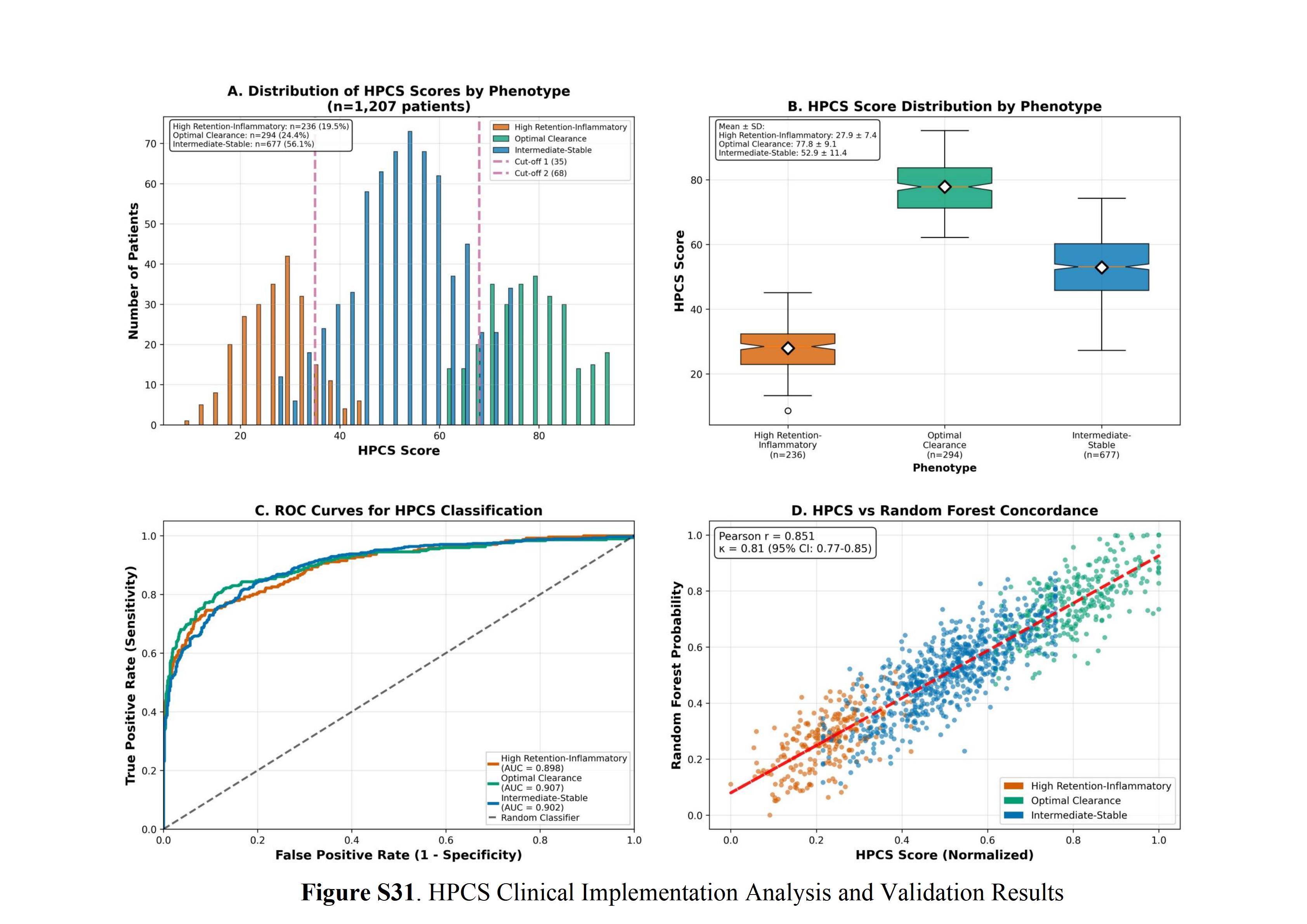

Supplement: Supplementary Material_34.jpg [file IRNF_A_2588961_SM0956.jpg]

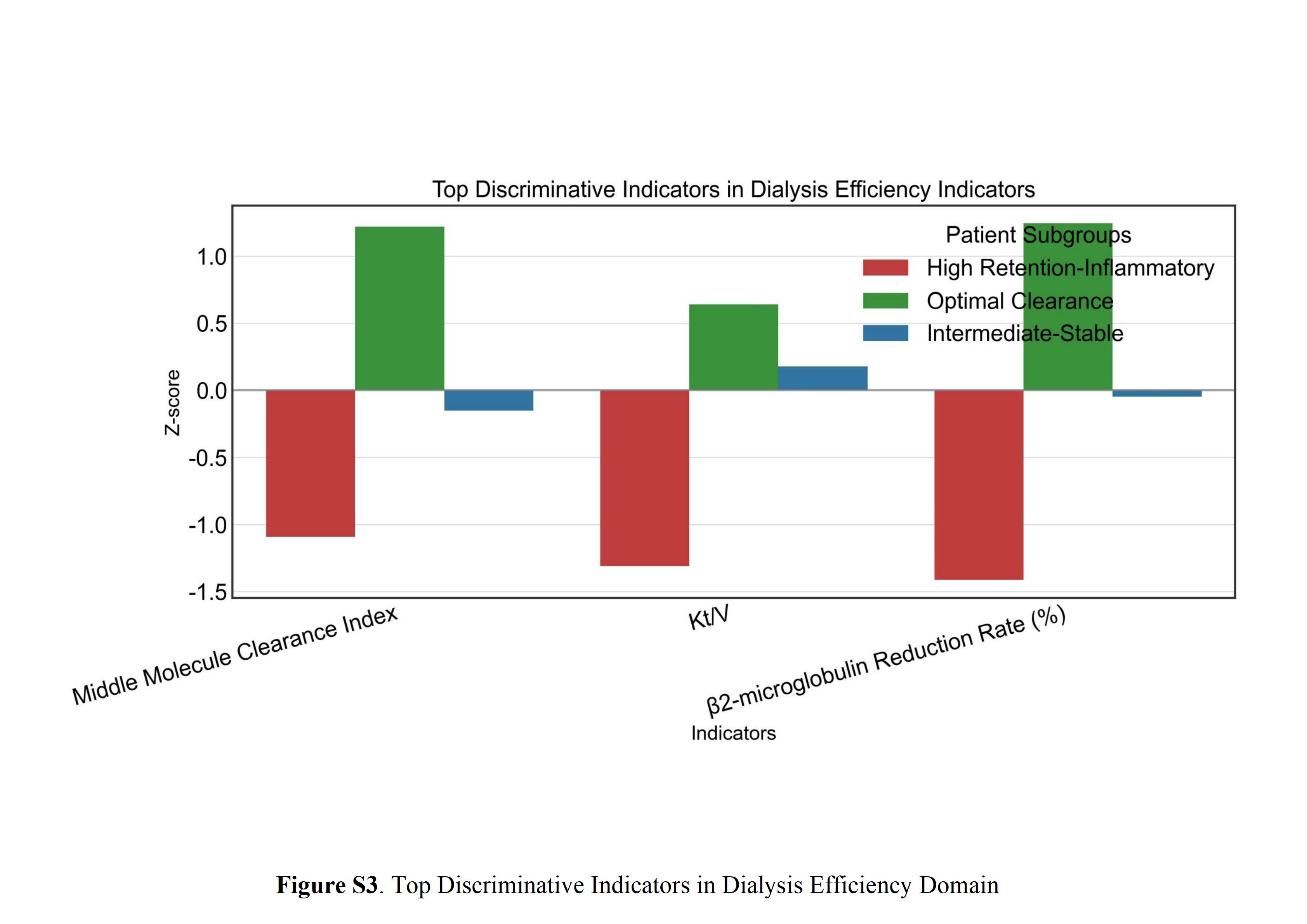

Supplement: Supplementary Material_03.jpg [file IRNF_A_2588961_SM0955.jpg]

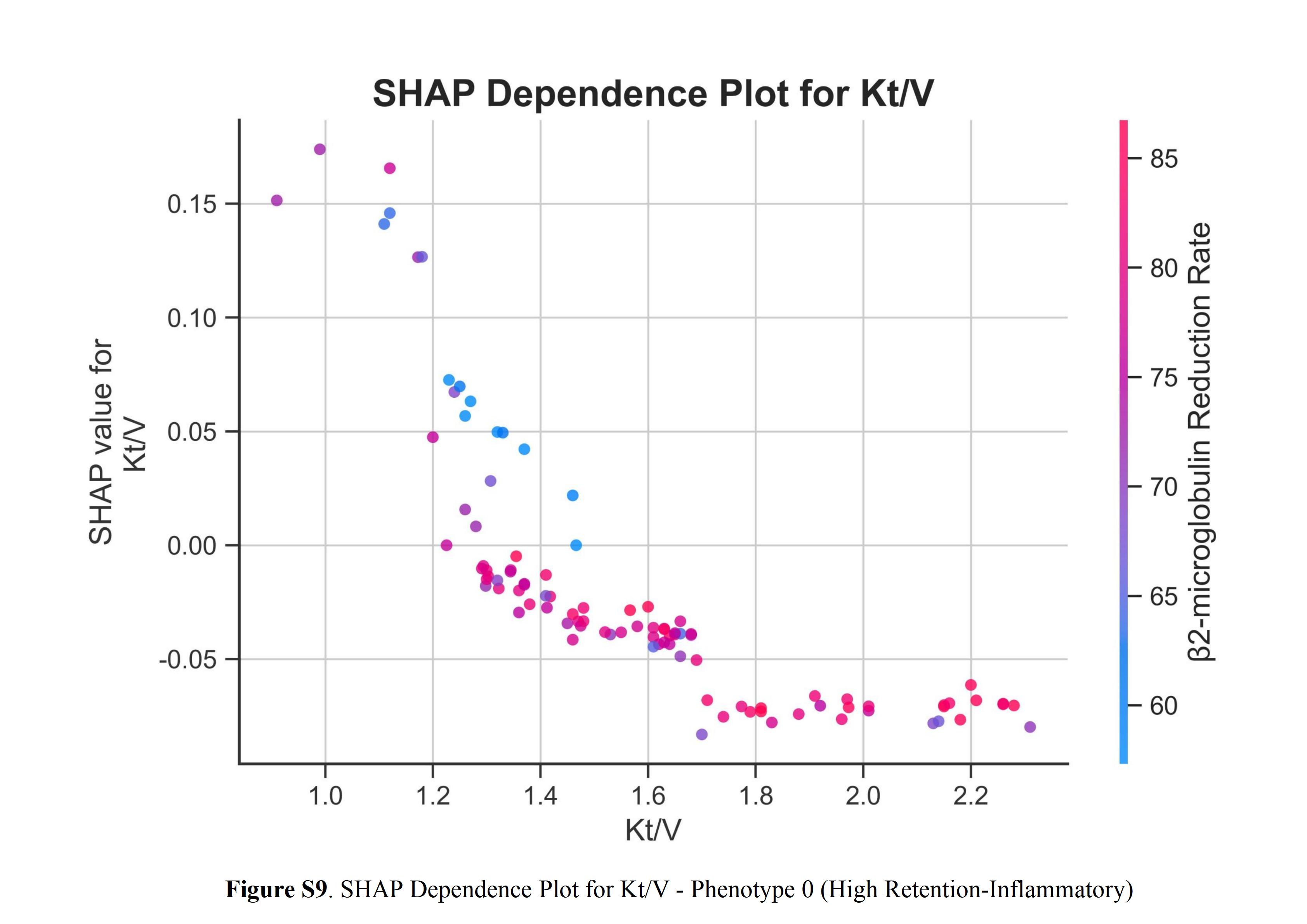

Supplement: Supplementary Material_09.jpg [file IRNF_A_2588961_SM0954.jpg]

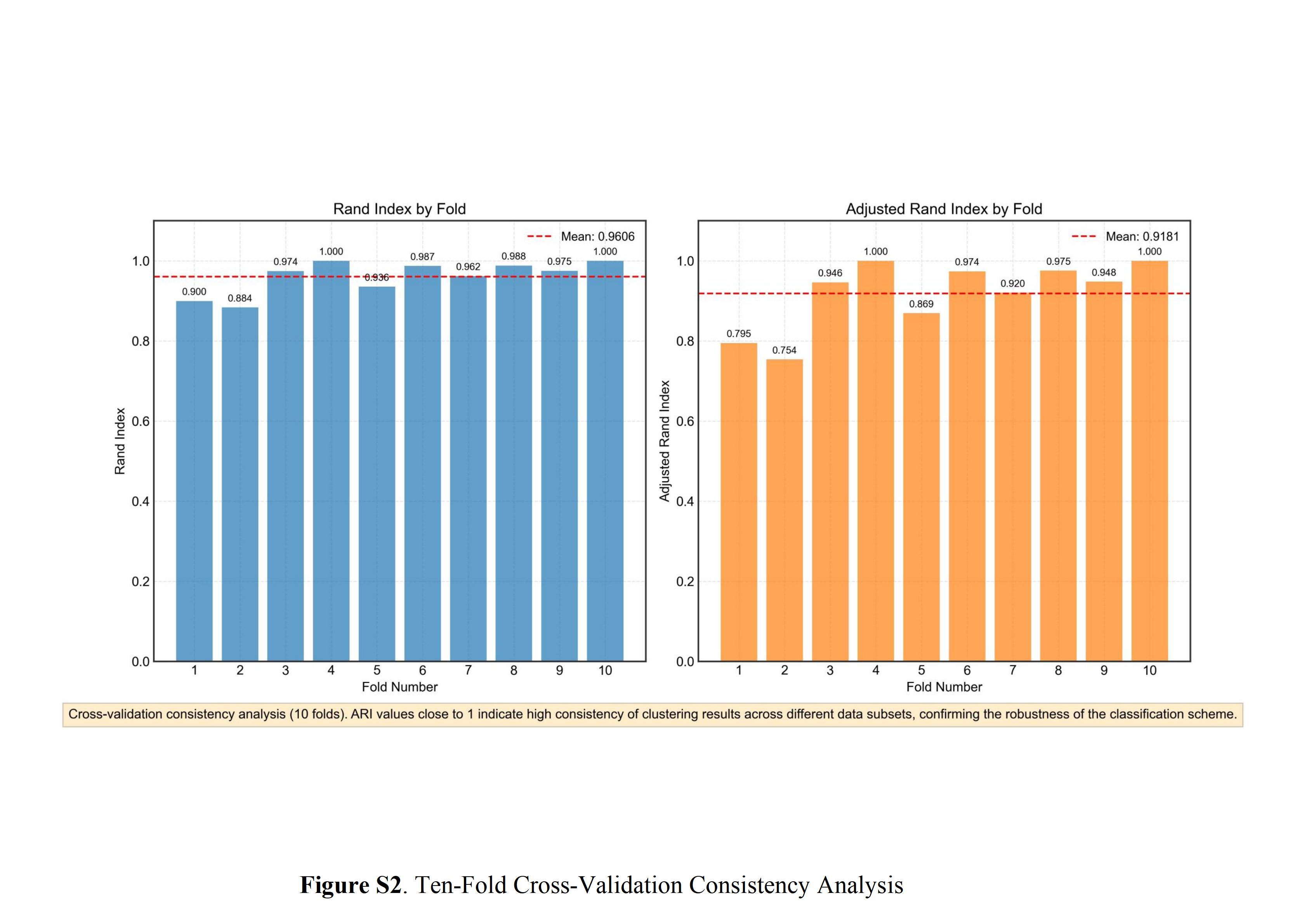

Supplement: Supplementary Material_02.jpg [file IRNF_A_2588961_SM0953.jpg]

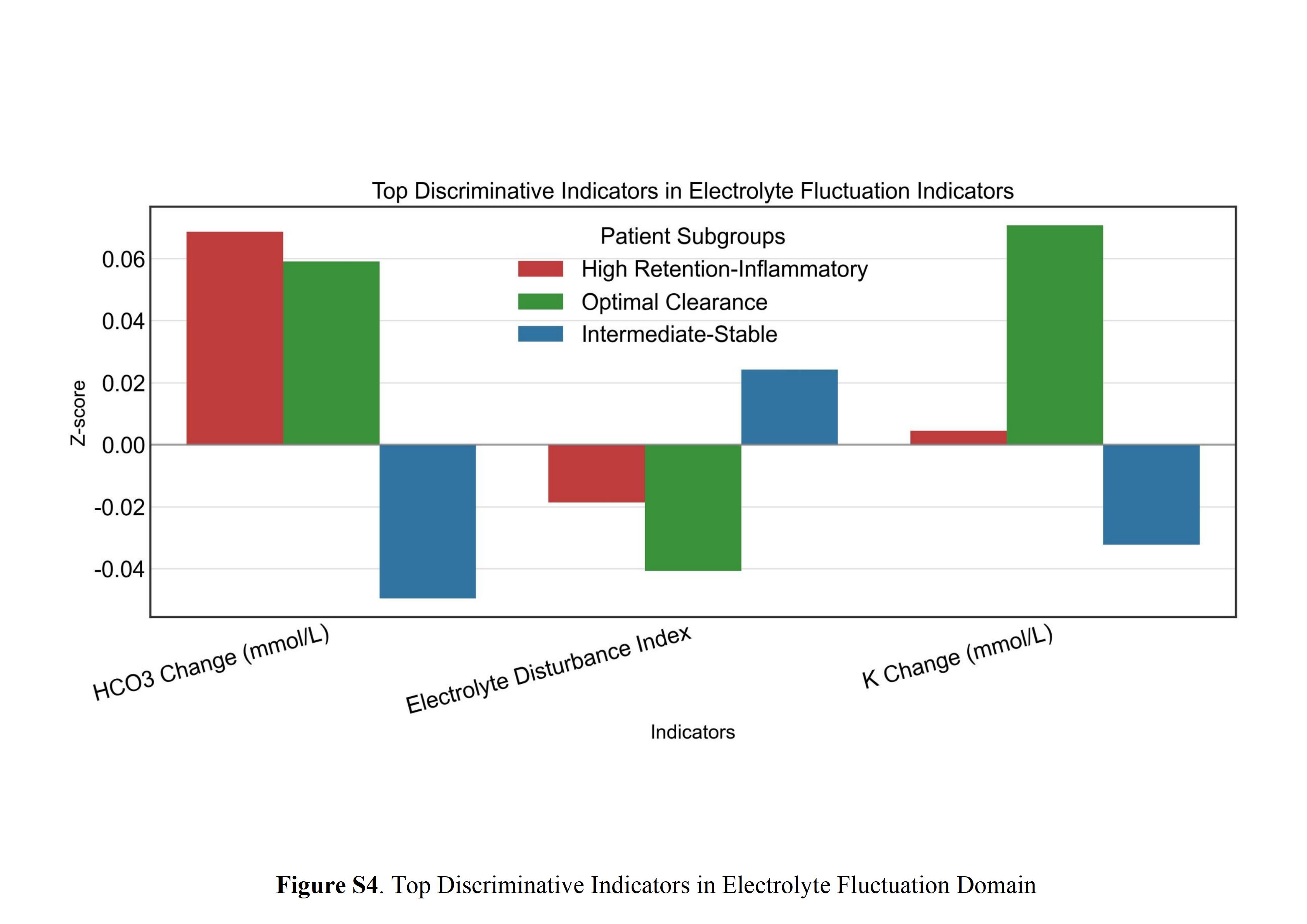

Supplement: Supplementary Material_04.jpg [file IRNF_A_2588961_SM0952.jpg]

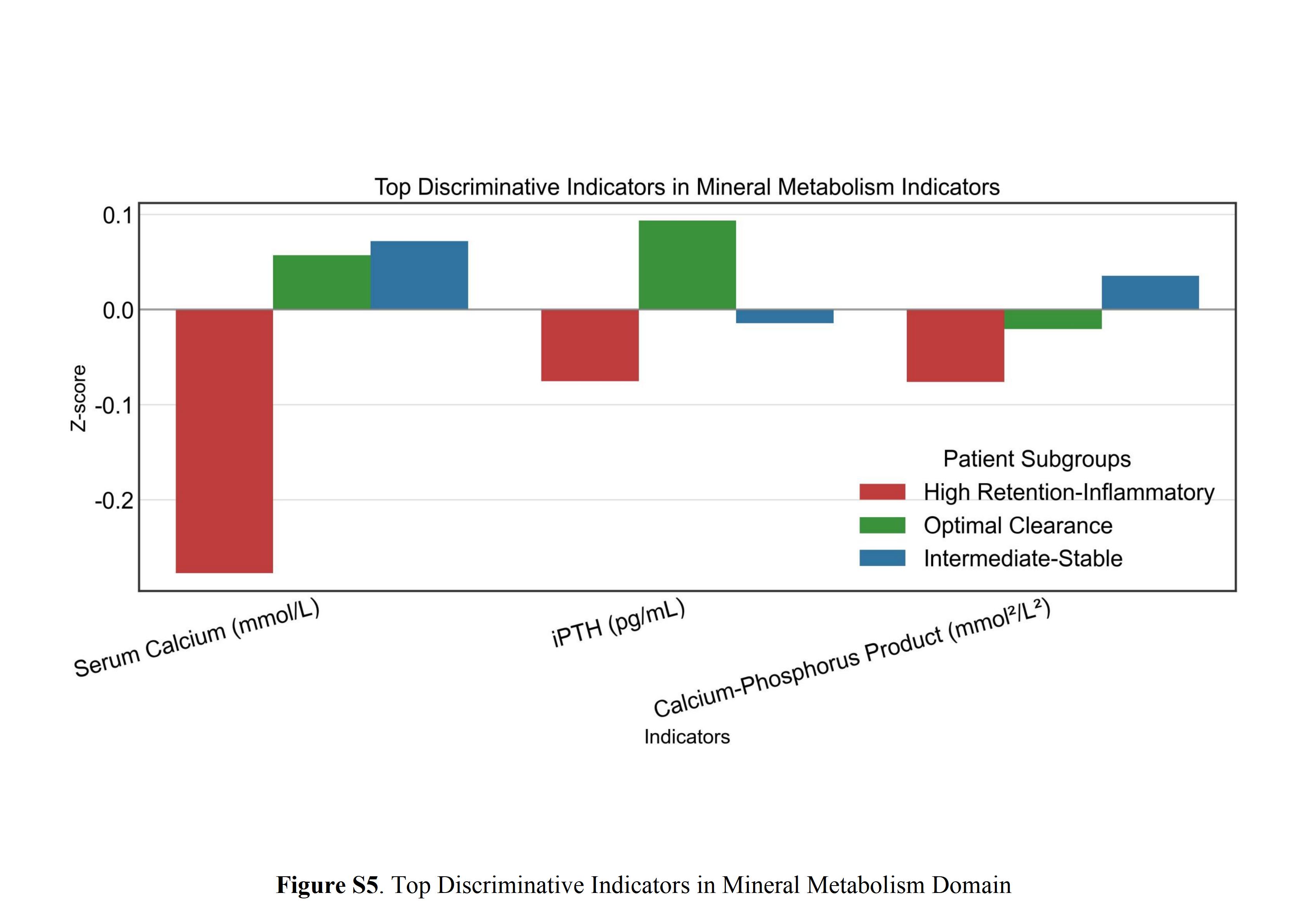

Supplement: Supplementary Material_05.jpg [file IRNF_A_2588961_SM0951.jpg]

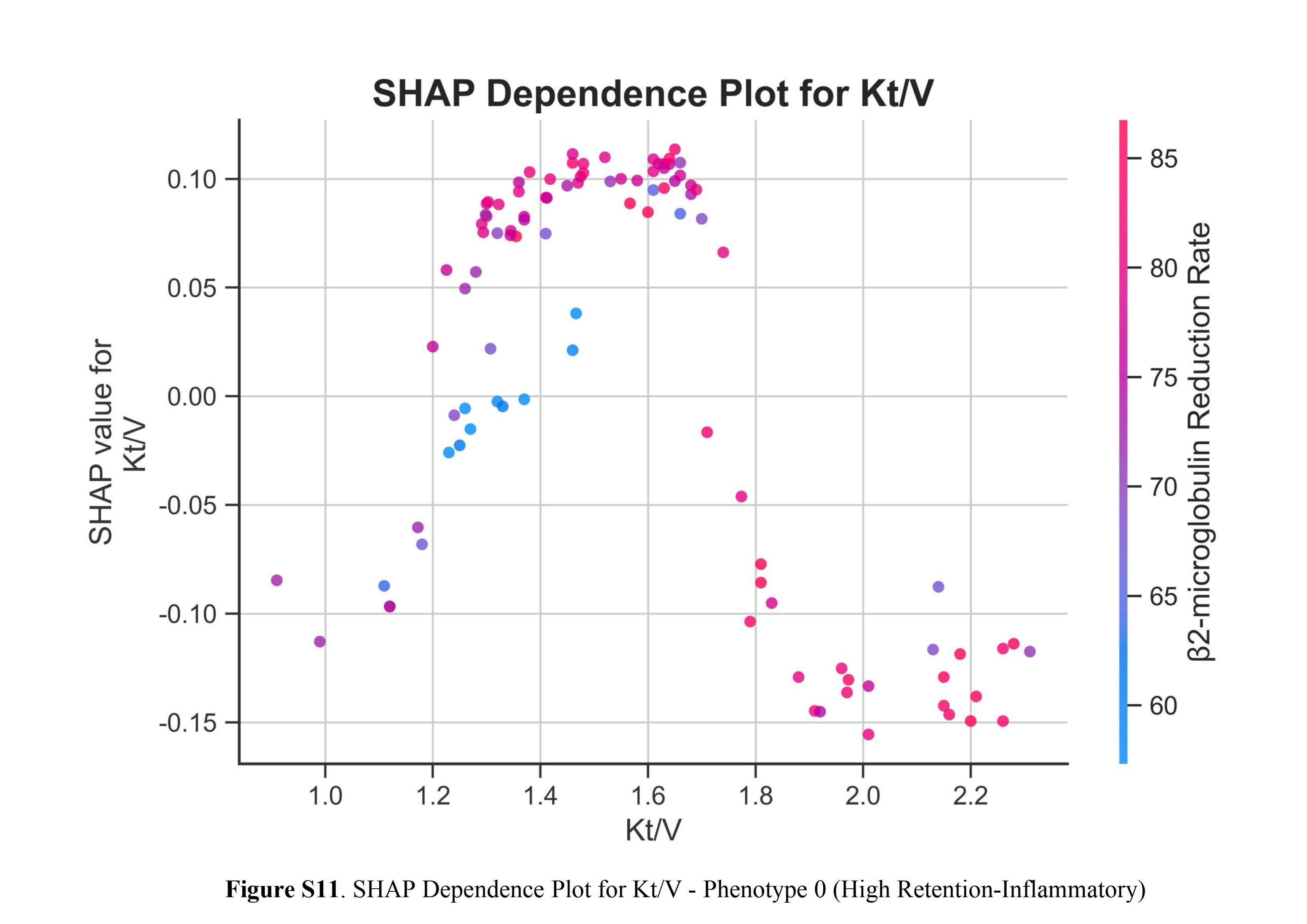

Supplement: Supplementary Material_11.jpg [file IRNF_A_2588961_SM0950.jpg]

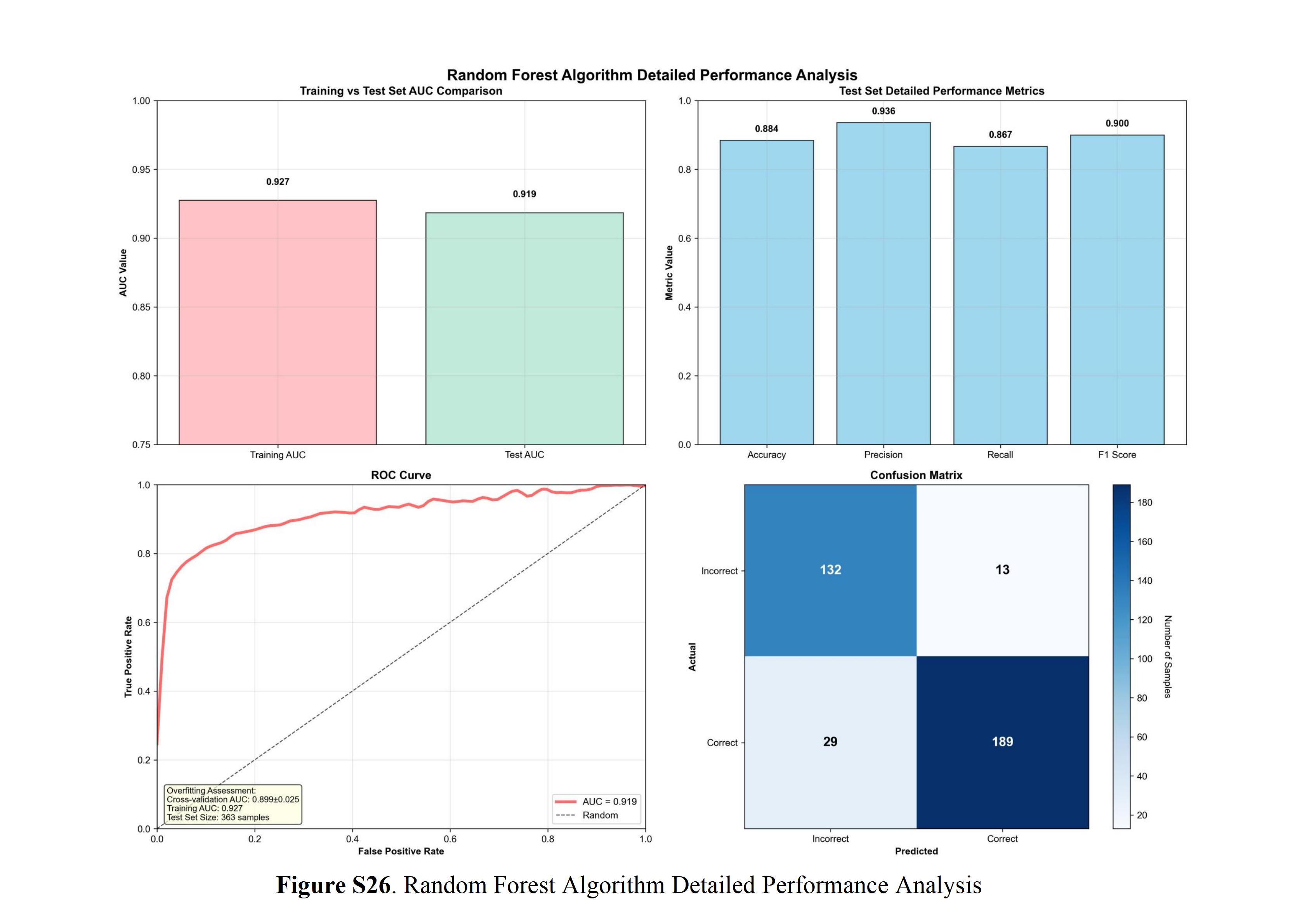

Supplement: Supplementary Material_26.jpg [file IRNF_A_2588961_SM0949.jpg]

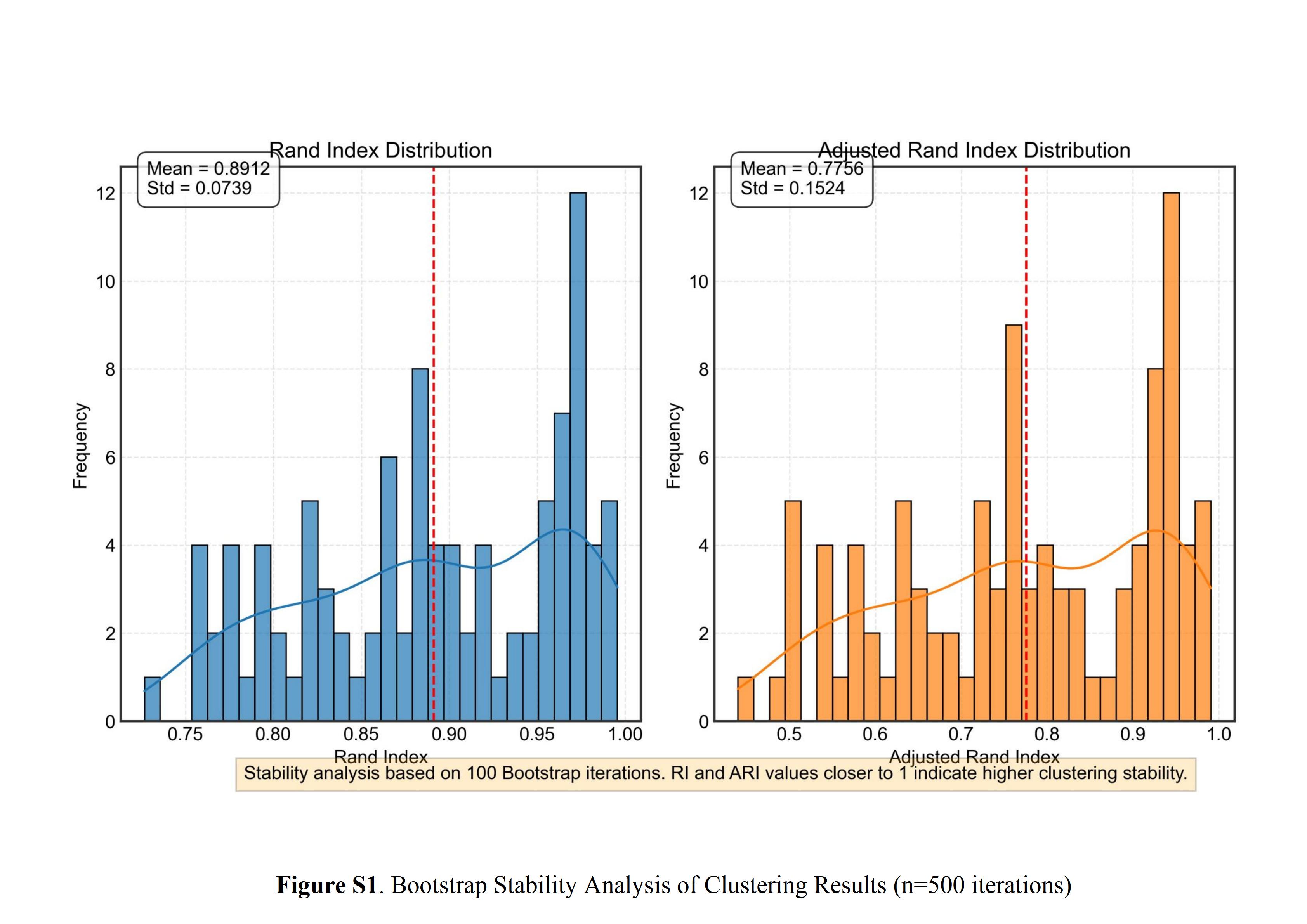

Supplement: Supplementary Material_01.jpg [file IRNF_A_2588961_SM0947.jpg]

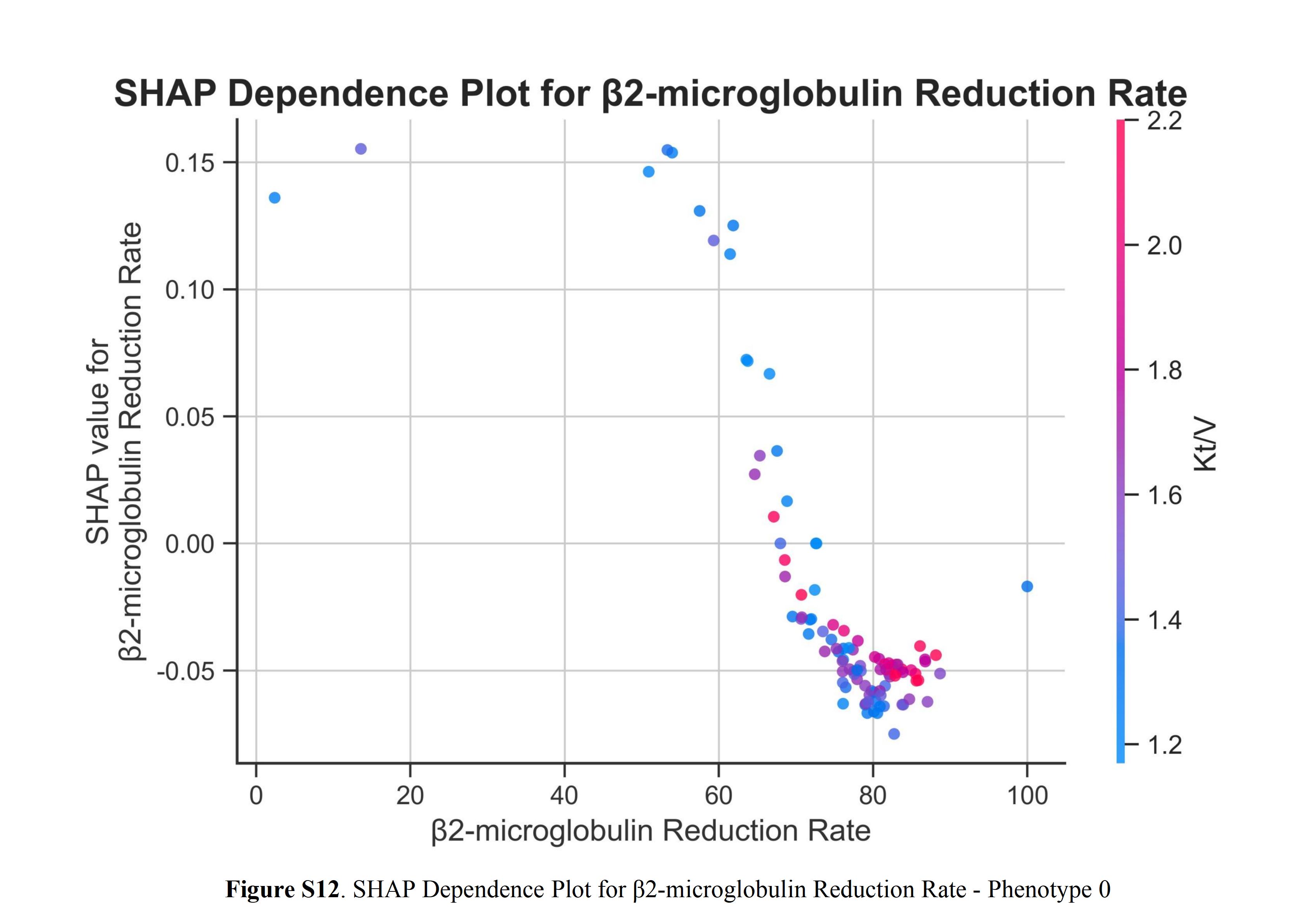

Supplement: Supplementary Material_12.jpg [file IRNF_A_2588961_SM0946.jpg]

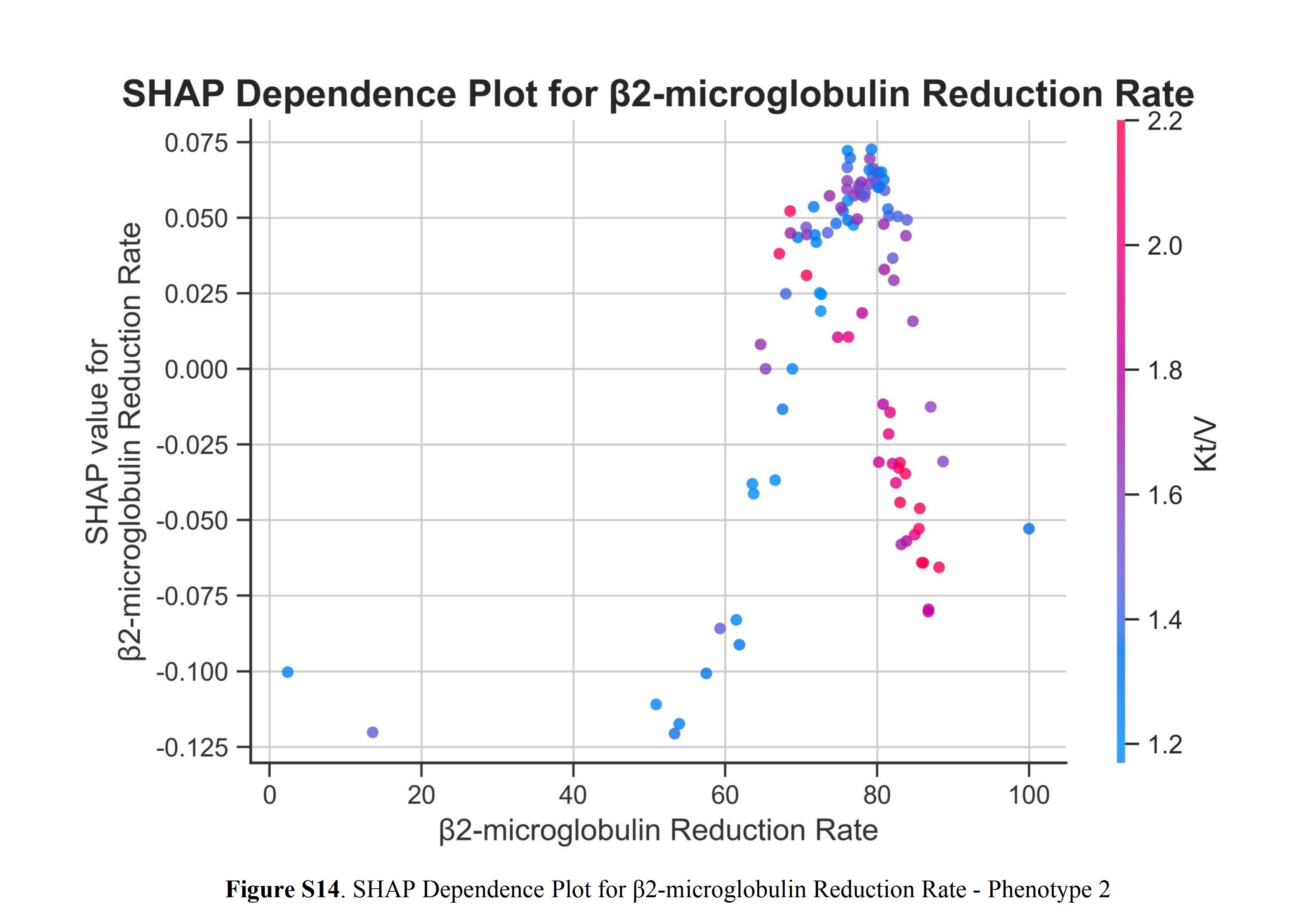

Supplement: Supplementary Material_14.jpg [file IRNF_A_2588961_SM0945.jpg]

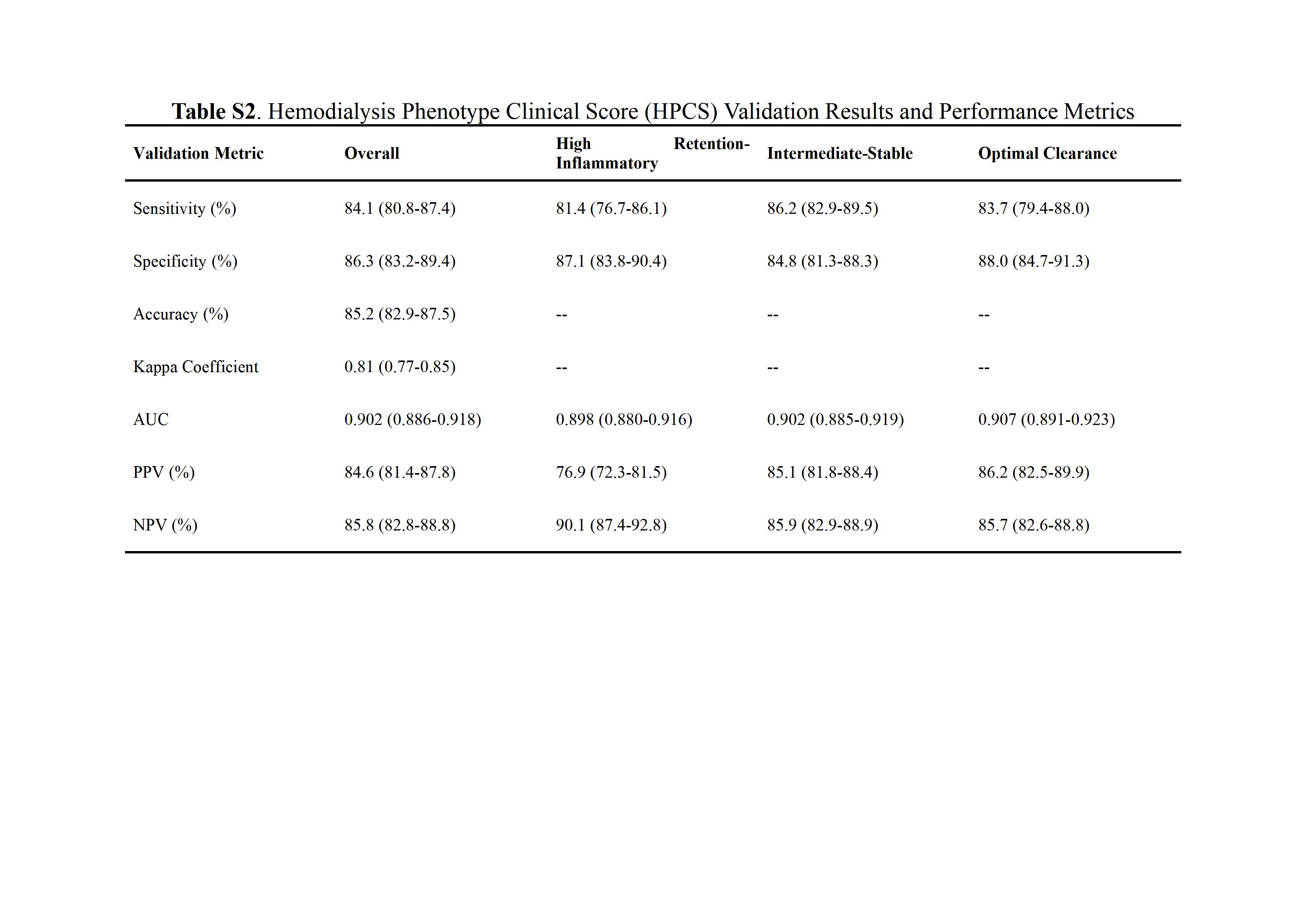

Supplement: Supplementary Material_33.jpg [file IRNF_A_2588961_SM0944.jpg]

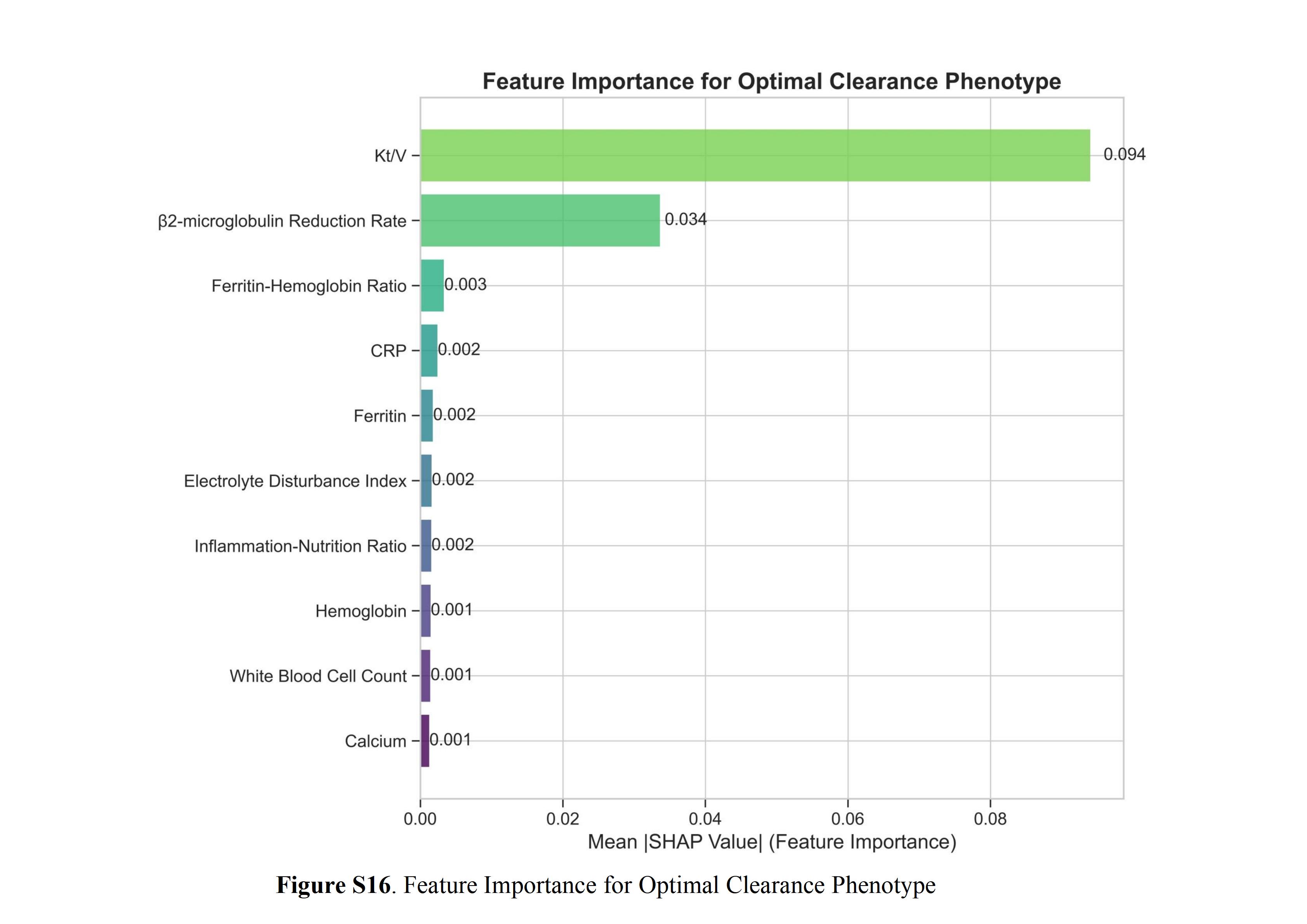

Supplement: Supplementary Material_16.jpg [file IRNF_A_2588961_SM0943.jpg]

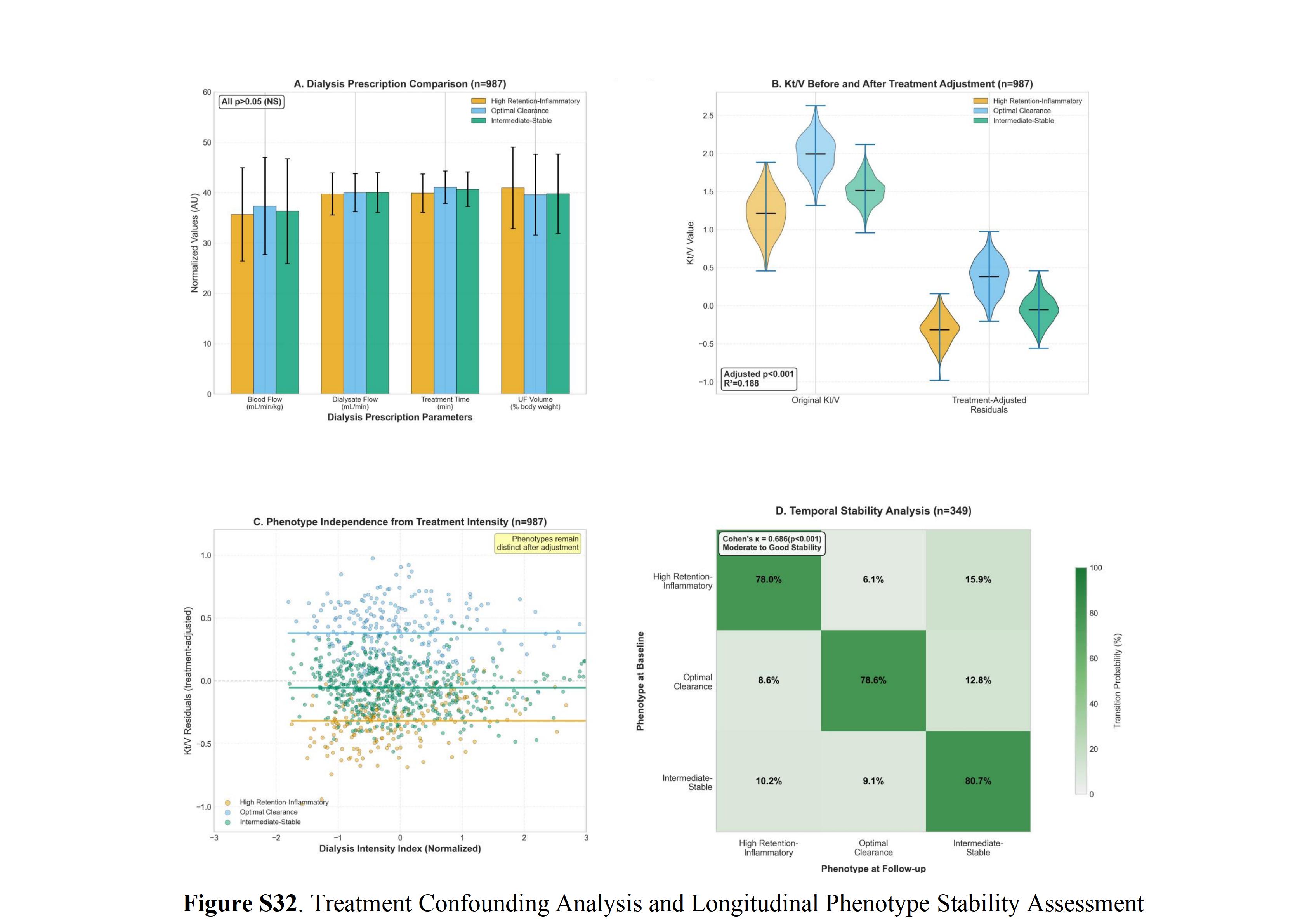

Supplement: Supplementary Material_37.jpg [file IRNF_A_2588961_SM0942.jpg]

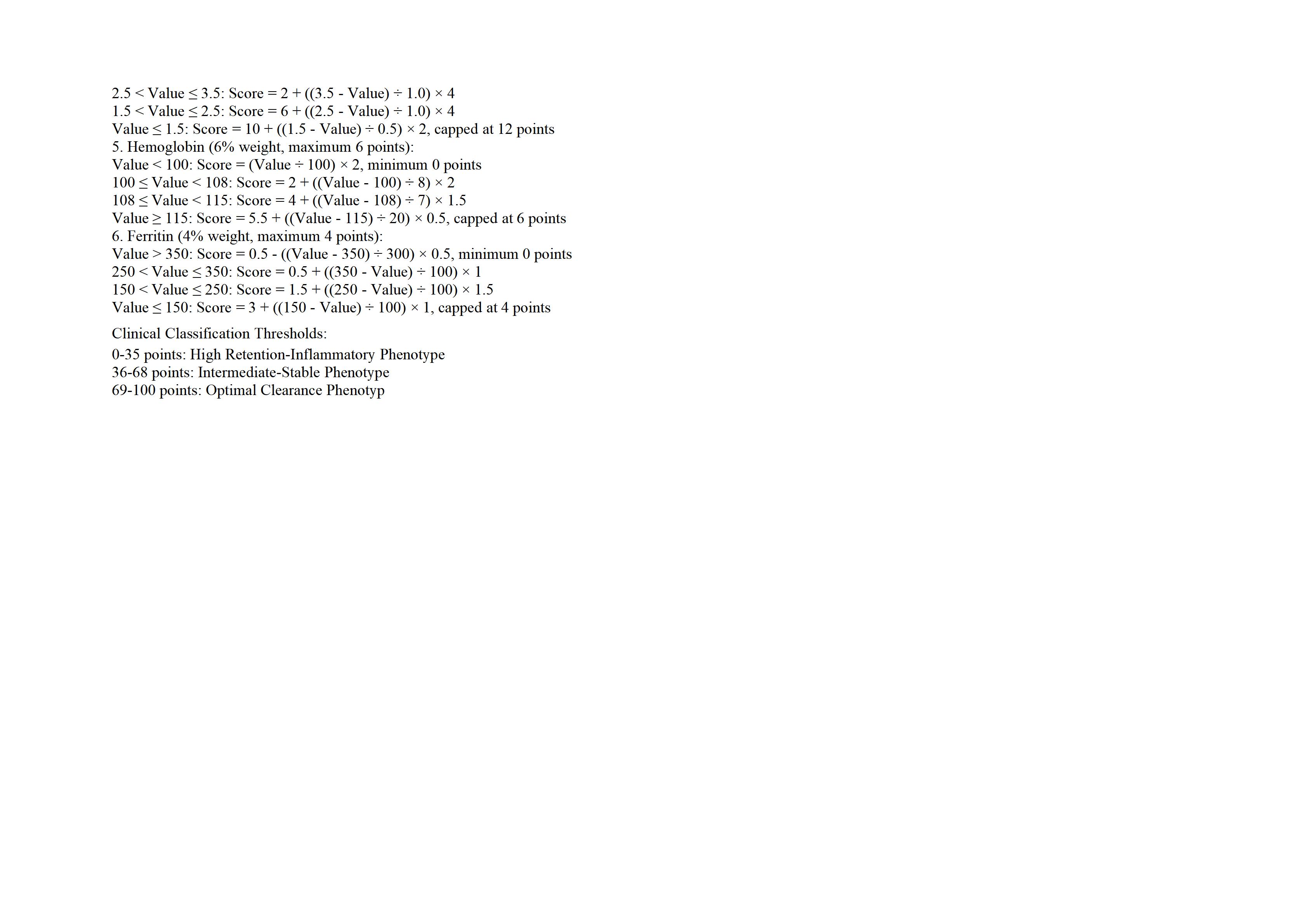

Supplement: Supplementary Material_32.jpg [file IRNF_A_2588961_SM0941.jpg]

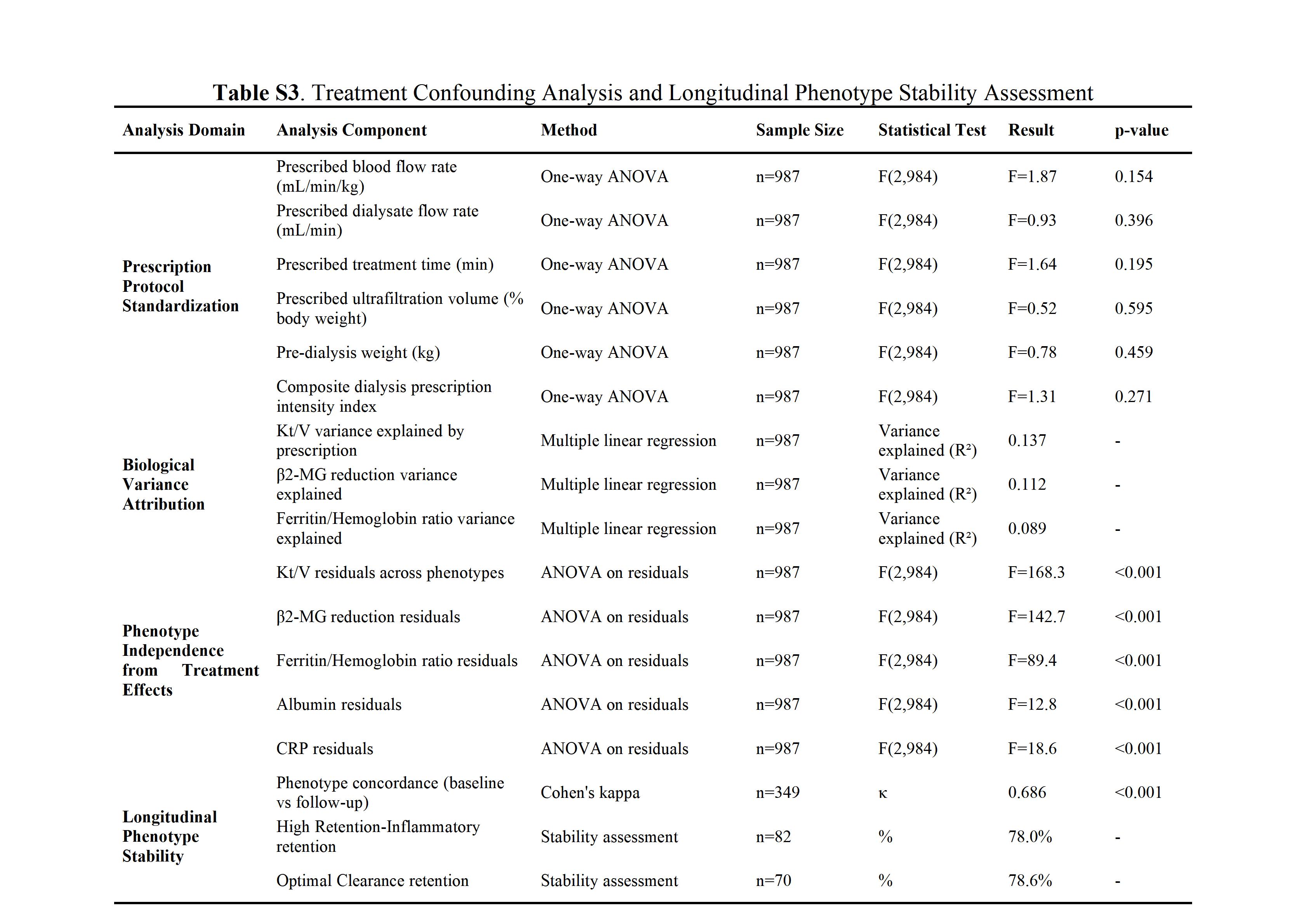

Supplement: Supplementary Material_35.jpg [file IRNF_A_2588961_SM0940.jpg]

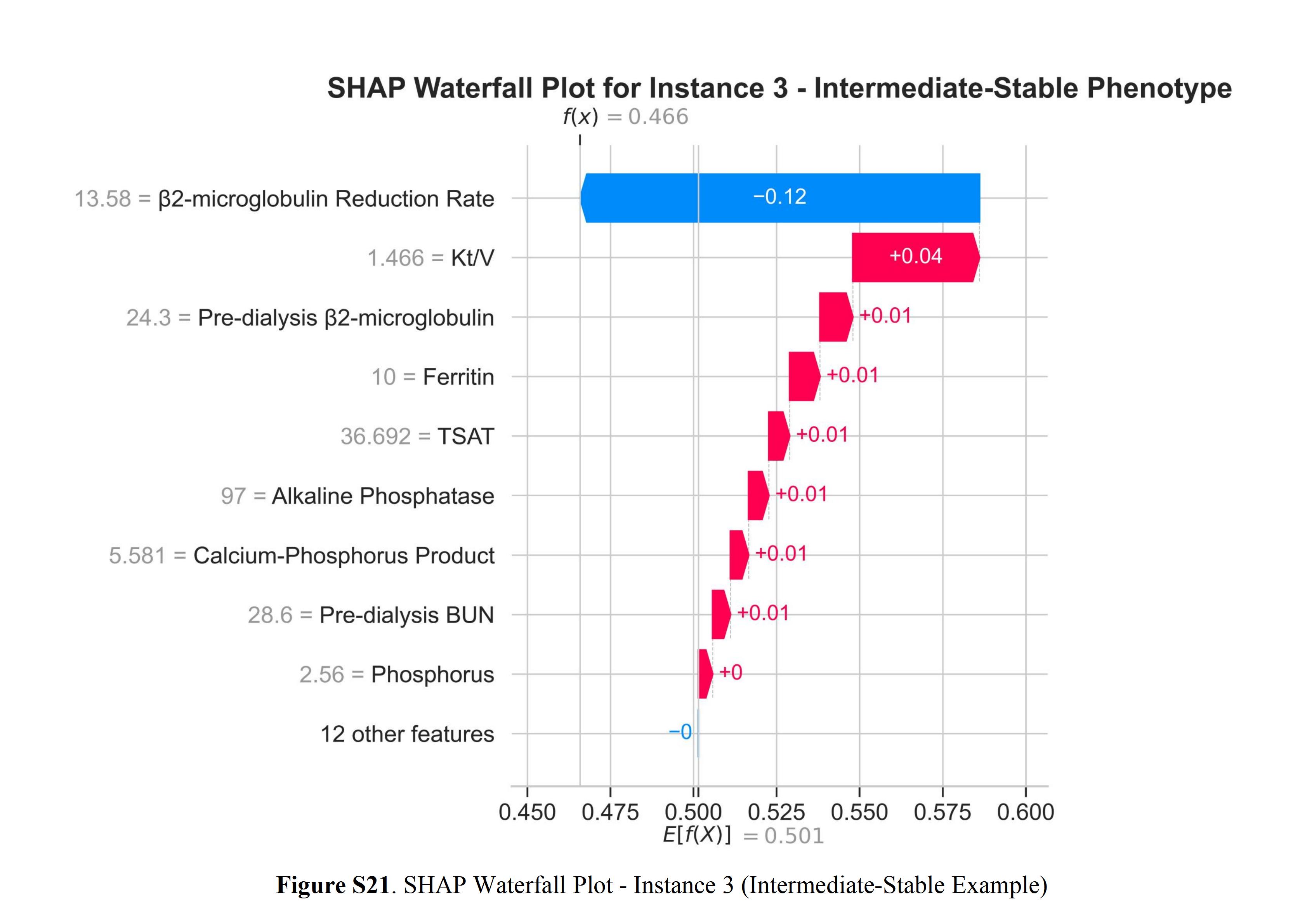

Supplement: Supplementary Material_21.jpg [file IRNF_A_2588961_SM0939.jpg]

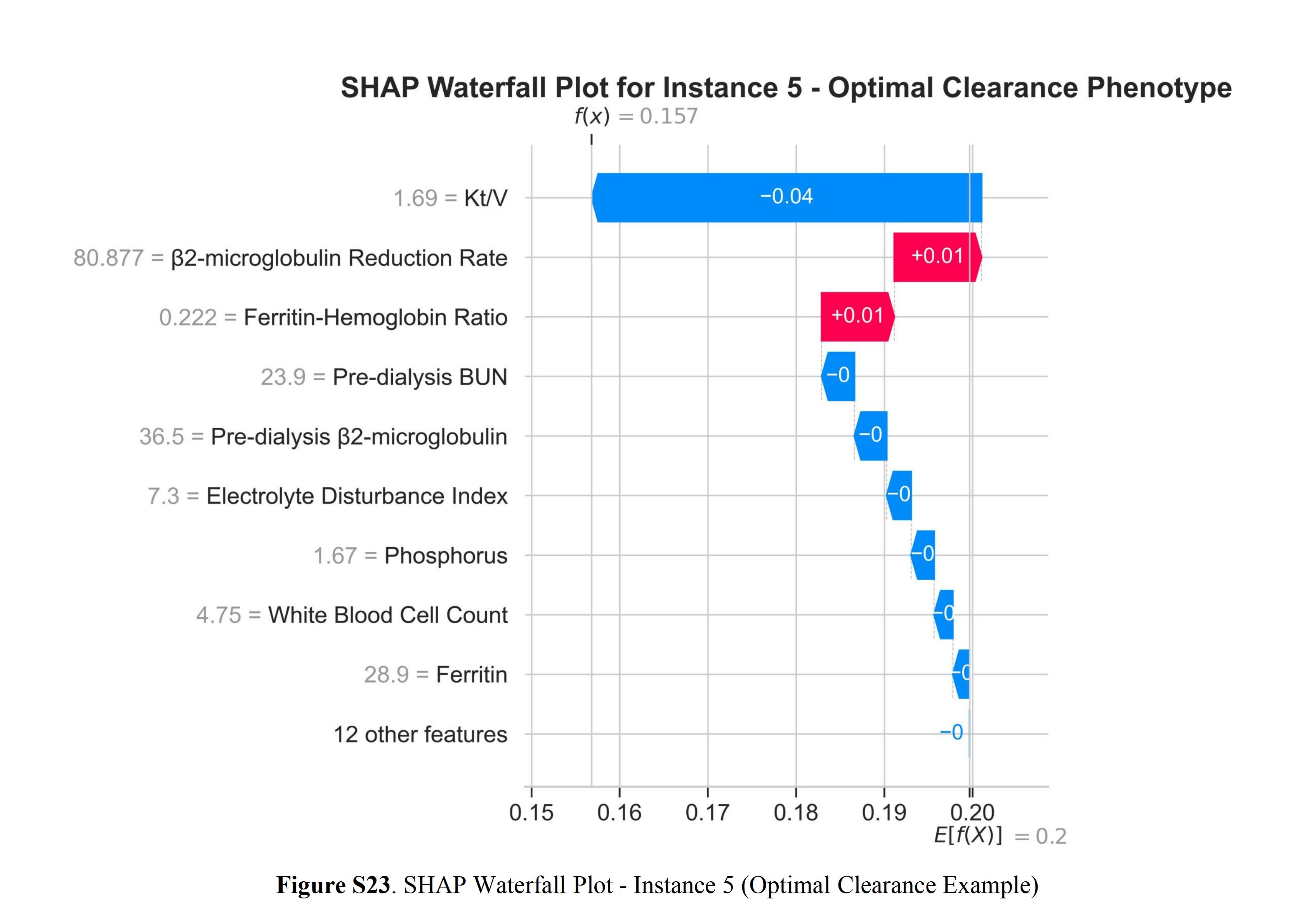

Supplement: Supplementary Material_23.jpg [file IRNF_A_2588961_SM0938.jpg]

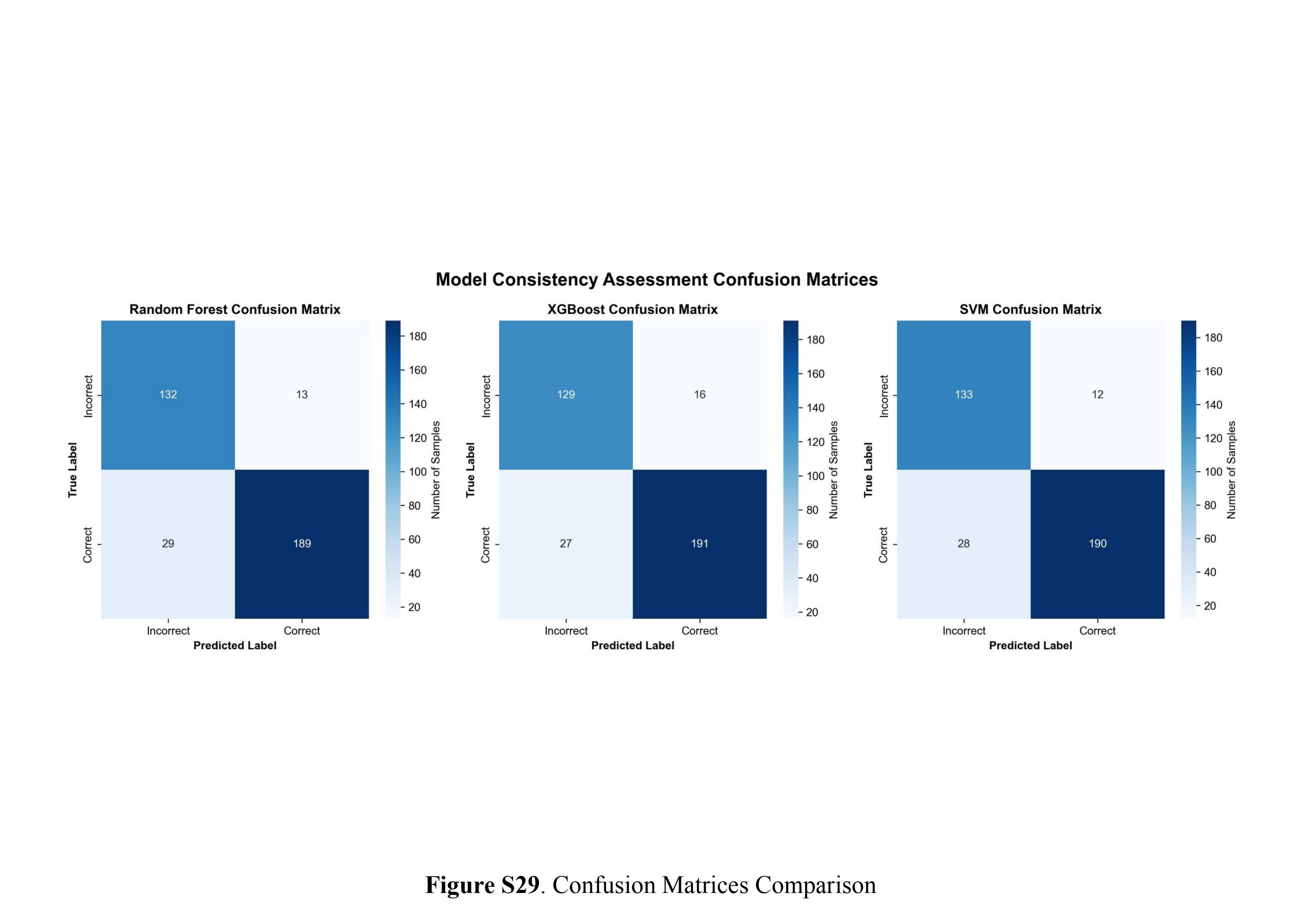

Supplement: Supplementary Material_29.jpg [file IRNF_A_2588961_SM0935.jpg]

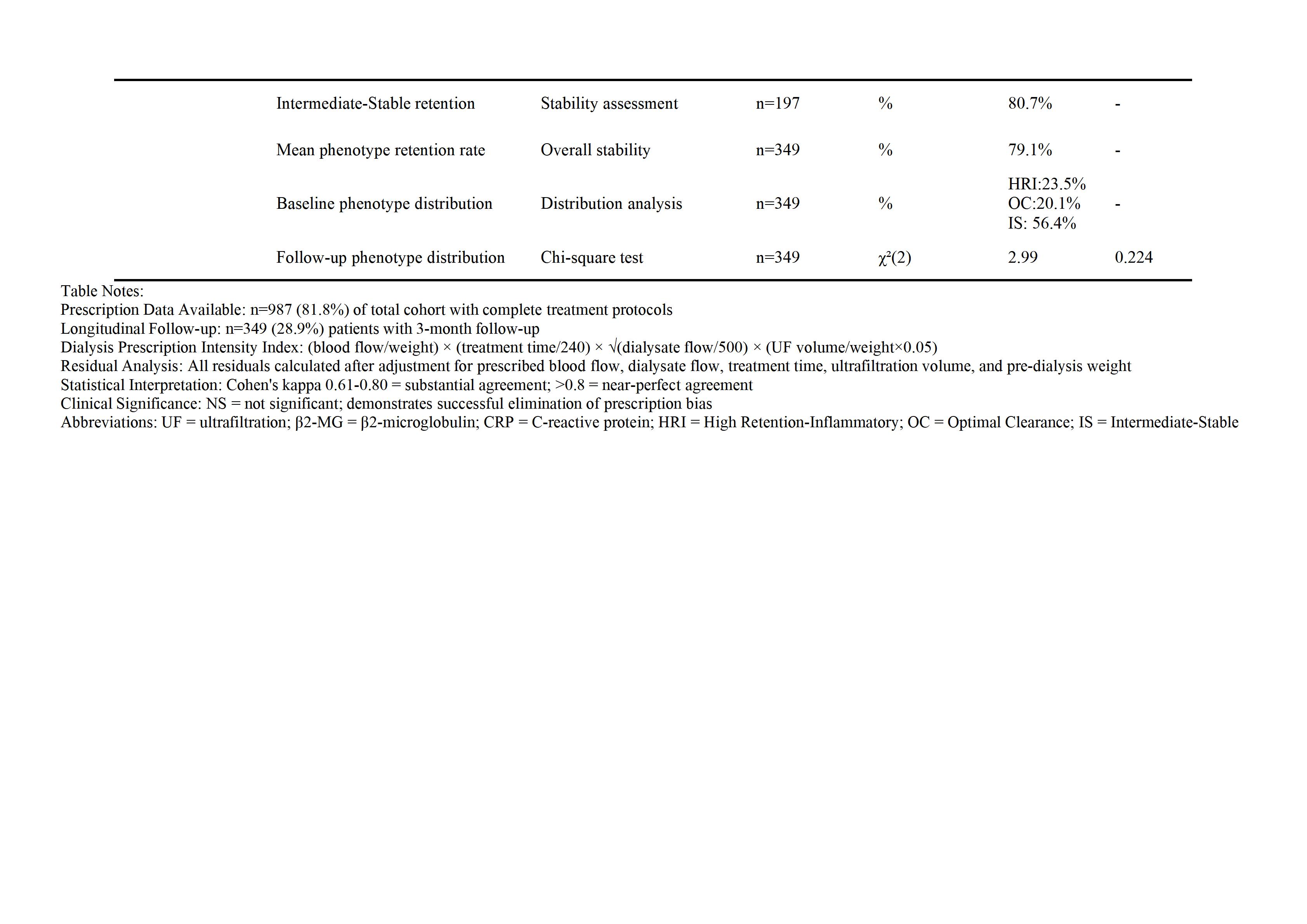

Supplement: Supplementary Material_36.jpg [file IRNF_A_2588961_SM0934.jpg]

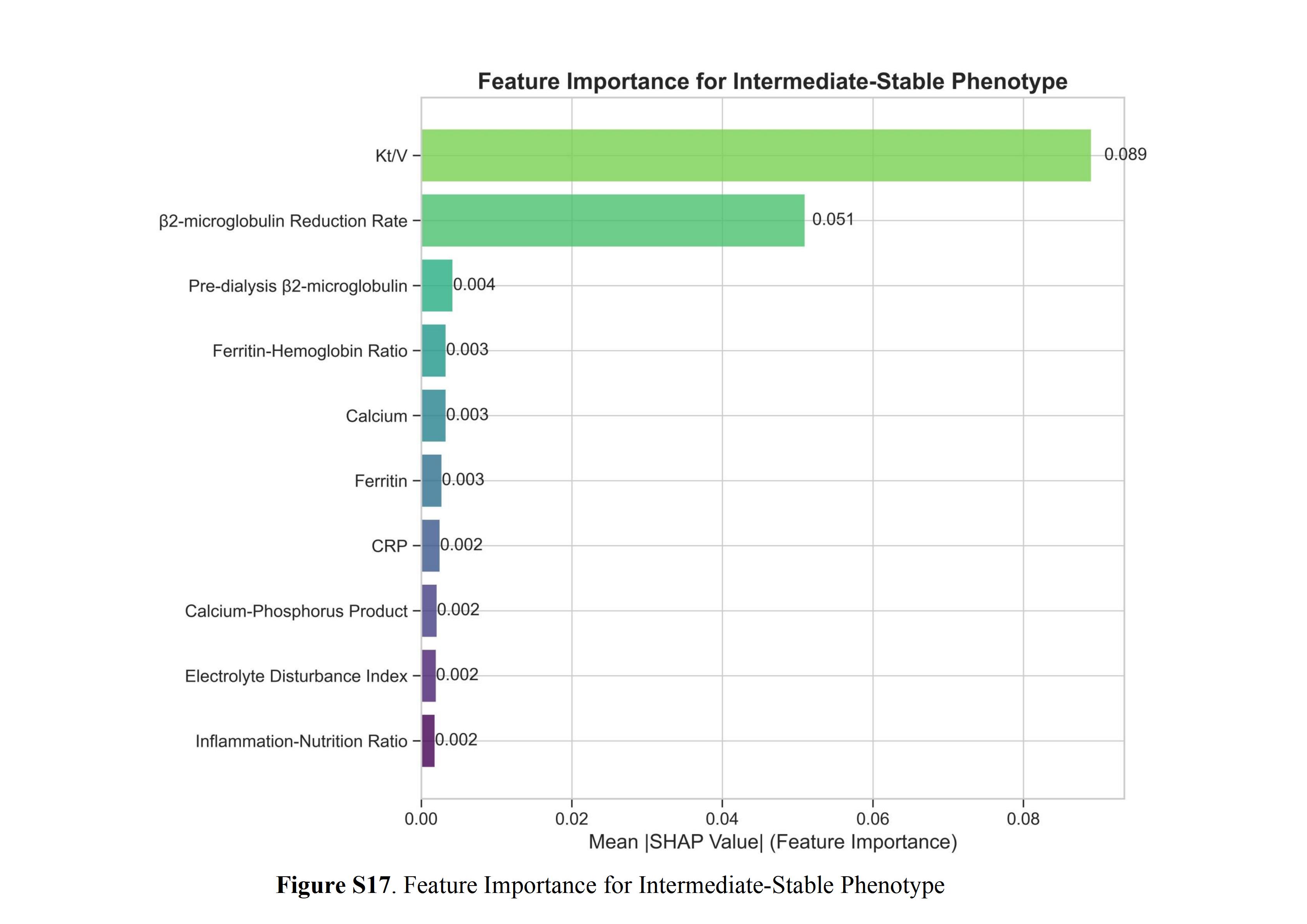

Supplement: Supplementary Material_17.jpg [file IRNF_A_2588961_SM0933.jpg]

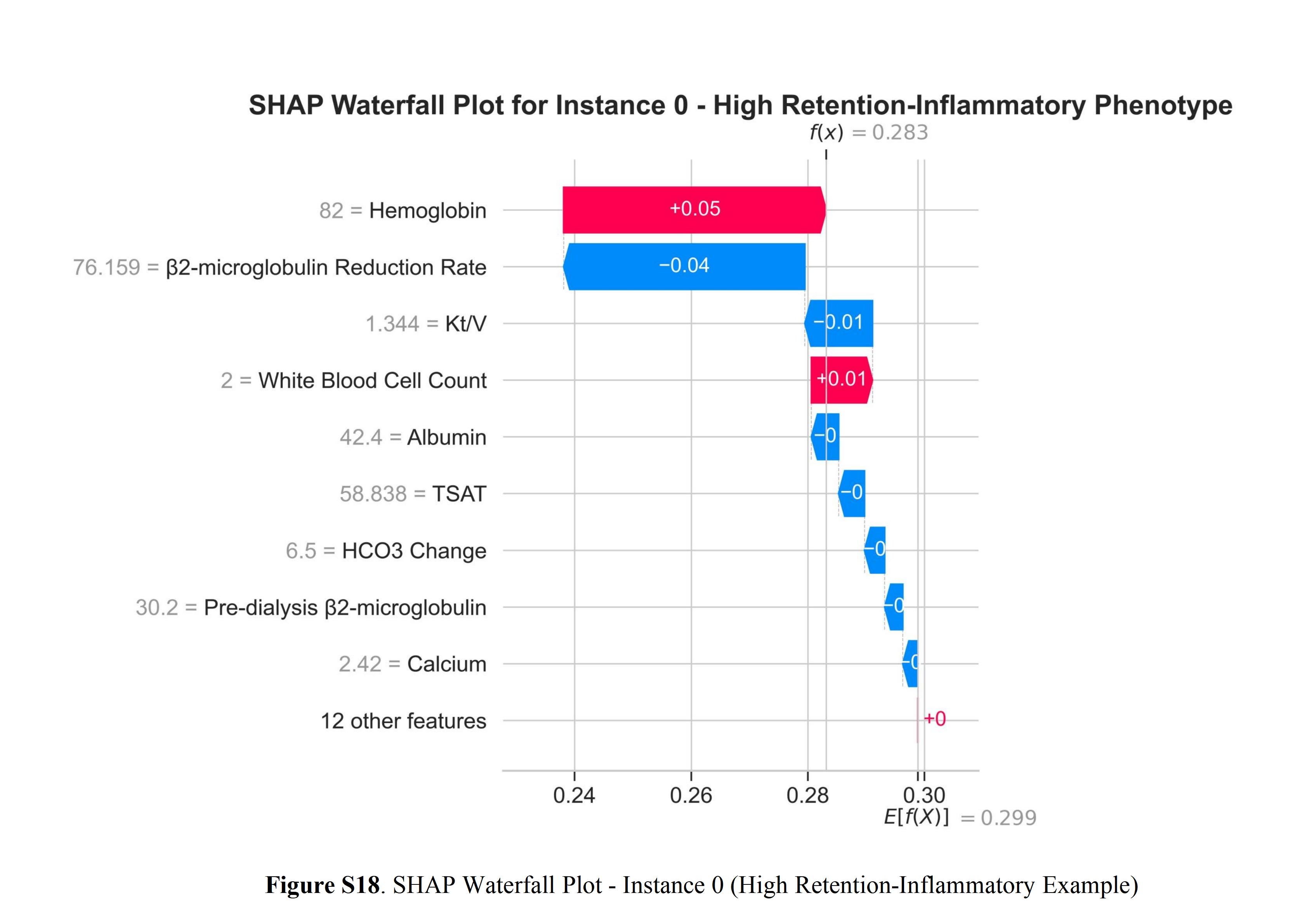

Supplement: Supplementary Material_18.jpg [file IRNF_A_2588961_SM0932.jpg]

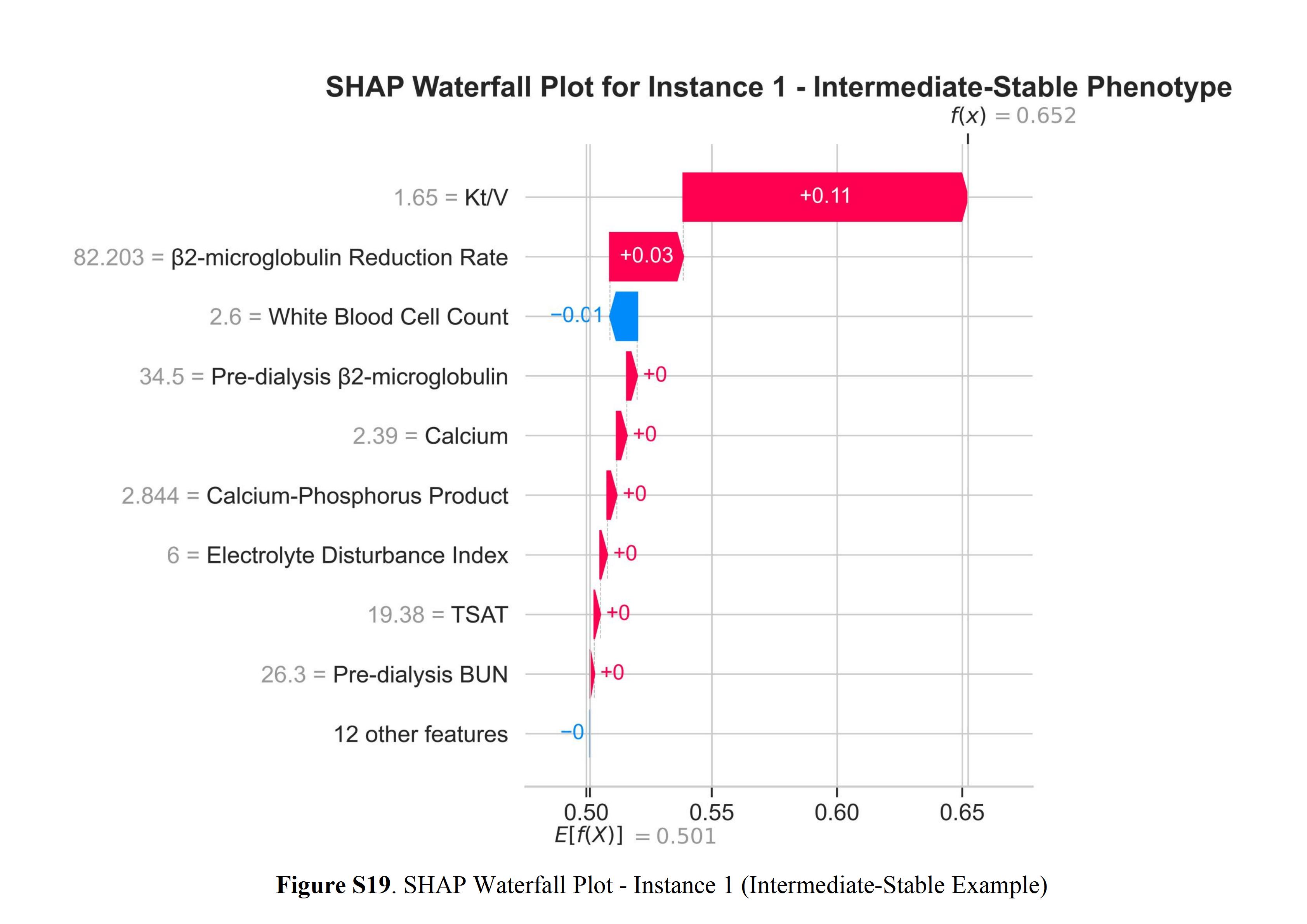

Supplement: Supplementary Material_19.jpg [file IRNF_A_2588961_SM0931.jpg]

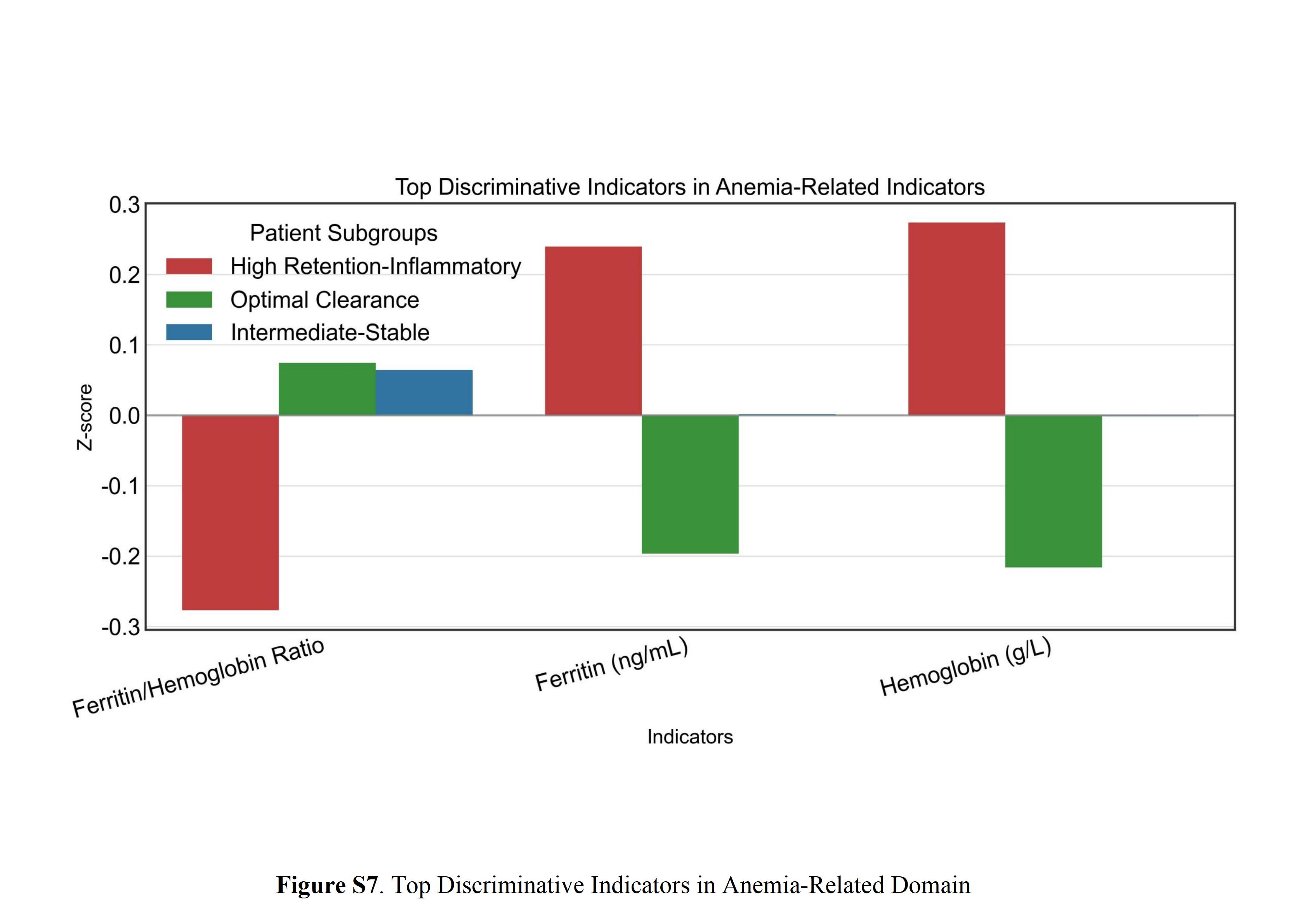

Supplement: Supplementary Material_07.jpg [file IRNF_A_2588961_SM0930.jpg]

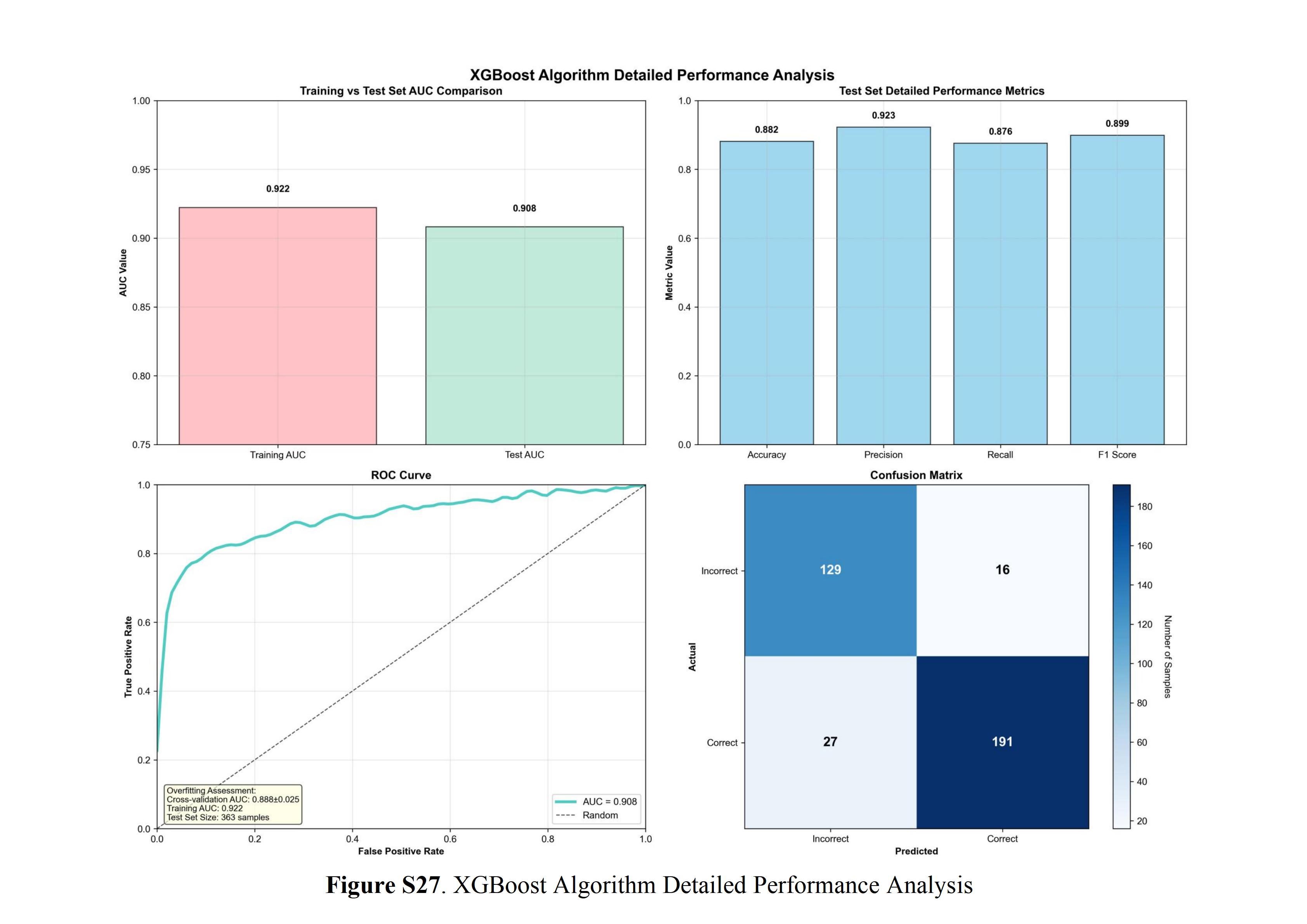

Supplement: Supplementary Material_27.jpg [file IRNF_A_2588961_SM0929.jpg]

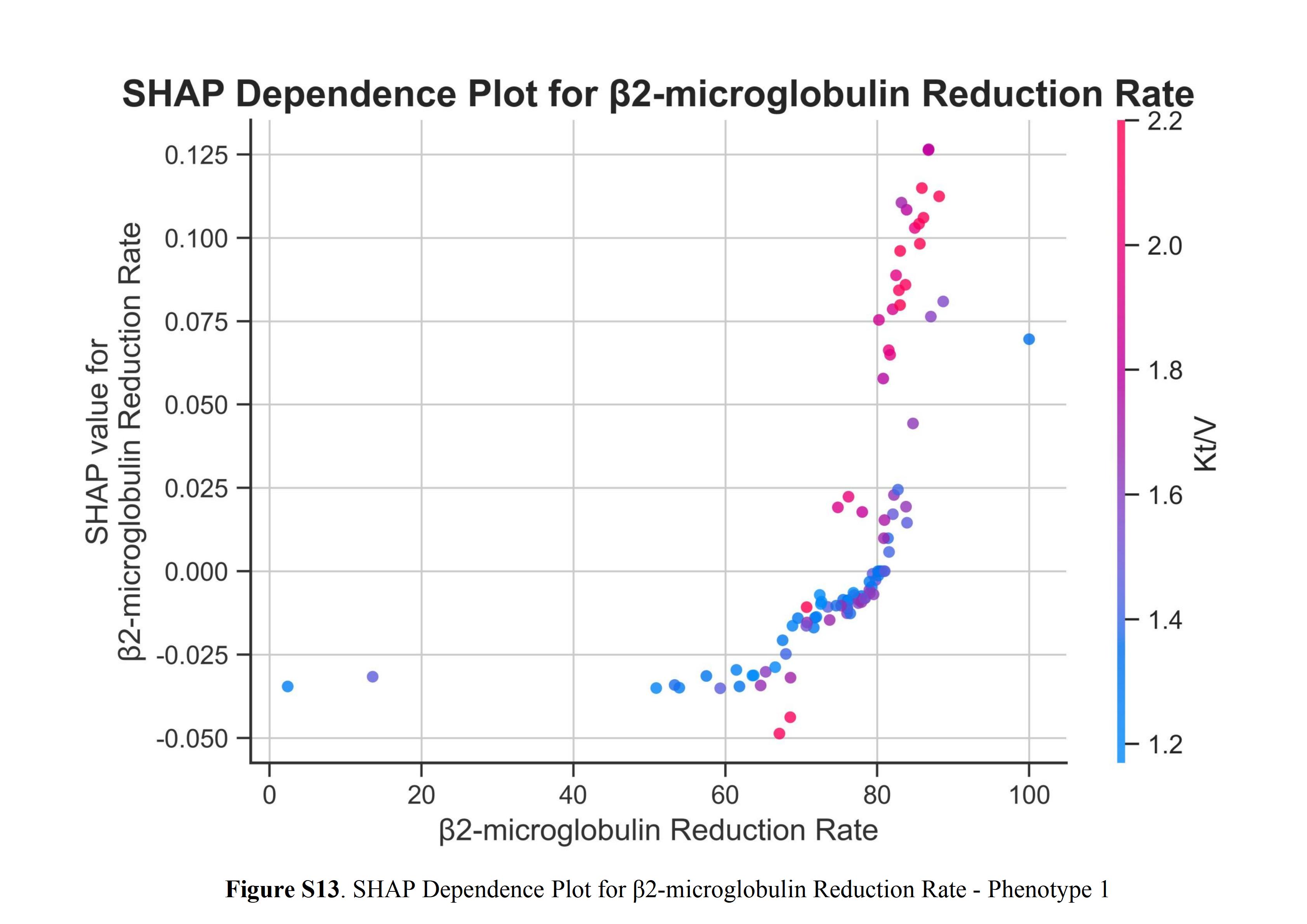

Supplement: Supplementary Material_13.jpg [file IRNF_A_2588961_SM0928.jpg]
