# Supplementary material for: Data-driven cluster analysis identifies three clinical phenotypes in hemodialysis patients
Source: Ren Fail. 2025 Nov 20;47(1):2588961. doi: 10.1080/0886022X.2025.2588961 (PMC12636659; doi:10.1080/0886022X.2025.2588961)
Supplement: Supplementary Material Table of Contents.docx [file IRNF_A_2588961_SM0937.docx]

**Supplementary Materials - Table of Contents**

**Section S1: Model Stability and Validation Analysis**

**Figure S1**. Bootstrap Stability Analysis of Clustering Results (n=500 iterations)

**Figure S2**. Ten-Fold Cross-Validation Consistency Analysis

**Section S2: Feature Discrimination Analysis by Domain**

**Figure S3**. Top Discriminative Indicators in Dialysis Efficiency Domain

**Figure S4**. Top Discriminative Indicators in Electrolyte Fluctuation Domain

**Figure S5**. Top Discriminative Indicators in Mineral Metabolism Domain

**Figure S6**. Top Discriminative Indicators in Inflammation/Nutrition Domain

**Figure S7**. Top Discriminative Indicators in Anemia-Related Domain

**Section S3: Multivariate Feature Relationships**

**Figure S8**. Parallel Coordinates Plot of Top 10 Features

**Section S4: SHAP (SHapley Additive exPlanations) Analysis**

**S4.1 Dependence Plots**

**Figure S9**. SHAP Dependence Plot for Kt/V - Phenotype 0 (High Retention-Inflammatory)

**Figure S10**. SHAP Dependence Plot for Kt/V - Phenotype 1 (Optimal Clearance)

**Figure S11**. SHAP Dependence Plot for Kt/V - Phenotype 2 (Intermediate-Stable)

**Figure S12**. SHAP Dependence Plot for β2-microglobulin Reduction Rate - Phenotype 0

**Figure S13**. SHAP Dependence Plot for β2-microglobulin Reduction Rate - Phenotype 1

**Figure S14**. SHAP Dependence Plot for β2-microglobulin Reduction Rate - Phenotype 2

**S4.2 Feature Importance Analysis**

**Figure S15**. Feature Importance for High Retention-Inflammatory Phenotype

**Figure S16**. Feature Importance for Optimal Clearance Phenotype

**Figure S17**. Feature Importance for Intermediate-Stable Phenotype

**S4.3 Individual Patient Classification Explanations**

**Figure S18**. SHAP Waterfall Plot - Instance 0 (High Retention-Inflammatory Example)

**Figure S19**. SHAP Waterfall Plot - Instance 1 (Intermediate-Stable Example)

**Figure S20**. SHAP Waterfall Plot - Instance 2 (Optimal Clearance Example)

**Figure S21**. SHAP Waterfall Plot - Instance 3 (Intermediate-Stable Example)

**Figure S22**. SHAP Waterfall Plot - Instance 4 (High Retention-Inflammatory Example)

**Figure S23**. SHAP Waterfall Plot - Instance 5 (Optimal Clearance Example)

**Section S5: Simplified 6-Parameter Model Performance**

**S5.1 Model Performance Overview**

**Figure S24**. ROC Curve Comparison of Three Machine Learning Algorithms

**Figure S25**. Model Performance Metrics Comparison

**S5.2 Individual Algorithm Analysis**

**Figure S26**. Random Forest Algorithm Detailed Performance Analysis

**Figure S27**. XGBoost Algorithm Detailed Performance Analysis

**Figure S28**. SVM Algorithm Detailed Performance Analysis

**S5.3 Model Consistency Assessment**

**Figure S29**. Confusion Matrices Comparison

**Figure S30**. Rand Index and Adjusted Rand Index Comparison

**S5.4 Clinical Scoring Framework Development**

**Table S1**. Hemodialysis Phenotype Clinical Score (HPCS) Calculation Guide

**Table S2**. Hemodialysis Phenotype Clinical Score (HPCS) Validation Results and Performance Metrics

**Figure S31**. HPCS Clinical Implementation Analysis and Validation Results

**Section S6: Sensitivity Analysis and Phenotype Stability Validation**

**Table S3**. Treatment Confounding Analysis and Longitudinal Phenotype Stability Assessment

**Figure S32**. Treatment Confounding Analysis and Longitudinal Phenotype Stability Assessment
